# Systematic exploration of accessible topologies of cage molecules *via* minimalistic models†

**DOI:** 10.1039/d3sc03991a

**Published:** 2023-10-11

**Authors:** Andrew Tarzia, Emma H. Wolpert, Kim E. Jelfs, Giovanni M. Pavan

**Affiliations:** a Department of Applied Science and Technology, Politecnico di Torino Corso Duca degli Abruzzi 24 10129 Torino Italy andrew.tarzia@polito.it giovanni.pavan@polito.it; b Department of Chemistry, Molecular Sciences Research Hub, Imperial College London, White City Campus Wood Lane London W12 0BZ UK; c Department of Innovative Technologies, University of Applied Sciences and Arts of Southern Switzerland, Polo Universitario Lugano Campus Est, Via la Santa 1 6962 Lugano-Viganello Switzerland

## Abstract

Cages are macrocyclic structures with an intrinsic internal cavity that support applications in separations, sensing and catalysis. These materials can be synthesised *via* self-assembly of organic or metal–organic building blocks. Their bottom-up synthesis and the diversity in building block chemistry allows for fine-tuning of their shape and properties towards a target property. However, it is not straightforward to predict the outcome of self-assembly, and, thus, the structures that are practically accessible during synthesis. Indeed, such a prediction becomes more difficult as problems related to the flexibility of the building blocks or increased combinatorics lead to a higher level of complexity and increased computational costs. Molecular models, and their coarse-graining into simplified representations, may be very useful to this end. Here, we develop a minimalistic toy model of cage-like molecules to explore the stable space of different cage topologies based on a few fundamental geometric building block parameters. Our results capture, despite the simplifications of the model, known geometrical design rules in synthetic cage molecules and uncover the role of building block coordination number and flexibility on the stability of cage topologies. This leads to a large-scale and systematic exploration of design principles, generating data that we expect could be analysed through expandable approaches towards the rational design of self-assembled porous architectures.

## Abstract 2

Minimal models of cages are used to map the accessible cage topologies based on building block properties.

## Graphical abstract

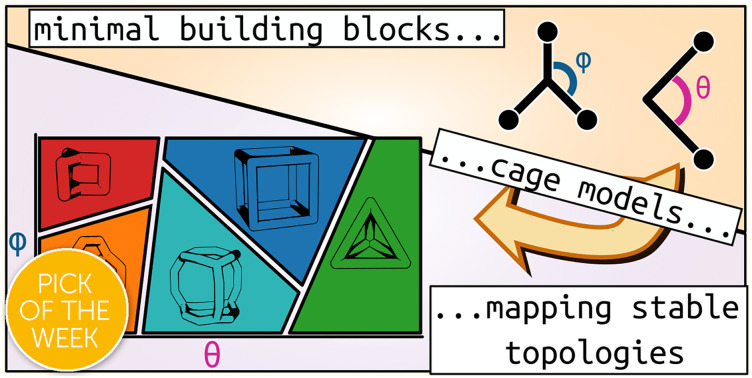

## 1 Introduction

Porous cage-like molecules are often formed through the bottom-up self-assembly of building blocks with varying connectivities and geometries, which transfers into the properties of the cage. The broad term “cage” applies to macrocyclic structures containing an intrinsic void^1^ widely studied for various types of applications, *e.g.* catalysis,^2^ sensing,^3^ separations,^4,5^ and as porous liquids.^6^ Their bottom-up self-assembly affords a vast pool of building blocks, allowing for the tunability of their properties. Cages can be entirely organic (porous organic cages, POCs)^7^ or contain metals (metal–organic cages, MOCs).^8^ Through rational selection from this vast chemical space and control of the self-assembly outcome and cage structure, researchers can achieve fine control over the spatial arrangement of atoms inside and outside cage compounds.

The successful synthesis of a specific cage is determined by whether the building blocks self-sort into the chemist's desired product (generally this is a single species, but could be a targeted mixture). The eventual cage structure has two related but distinct structural characteristics: topology and geometry. Cage topology strictly includes information about the connectivity of building blocks in the cage; throughout, we use the definition introduced by Santolini *et al.*^9^ This terminology is commonly used in the POC literature but is translatable to any cage-like molecule. The cage geometry describes the coordinates of atoms or building blocks in the cage structure, which has been closely linked to regular shapes or polyhedra while rigid building blocks are used.^10^ Indeed, under these conditions, building block geometry and stoichiometry can predict topological outcomes, which infer geometrical properties. But researchers are targeting more complex structures^11,12^ (towards improved tunability) through flexible, unsymmetrical, or mixed components.^13–16^ Therefore, the degrees of freedom to be considered will only grow, making the use of existing design rules for cages less viable.

Computational chemistry can help to simplify the relationship between building blocks and self-sorting outcomes by predicting or rationalising the structure and energetics of different potential topologies, showing, for example, whether a particular combination of building blocks and topology are accessible.^17–21^ Structure generation and evaluation of the relative stability of different topologies can help drive or rationalise experimental efforts.^16,22–26^ However, these calculations are costly or too approximate (often neglecting solvent/ion effects and flexibility), and the thermodynamics of each step is not always enough of the picture (*e.g.*, when kinetic traps are present). When introducing asymmetry, for example, combinatorial explosion quickly leads to an intractable number of isomers, topologies or configurations that must be studied to consider self-sorting outcomes.^23,27,28^ Therefore, new approaches to tackling these prediction and rationalisation problems are needed.

For more efficient modelling, coarse-grained (CG) or minimalistic models simplify and approximate molecular representations to low-resolution models. By nature, their low resolution provides access to low-cost simulations, allowing larger systems to be modelled for longer times, or in this case, allowing for vast numbers of systems to be modelled. In particular, minimalistic models (or “toy models”) allow us to zoom out to very low resolutions and systematically generate large amounts of qualitative data to learn from, eventually translating that into atomistic models or experimental designs.^29,30^ With complete control over the model parameters, toy models allow for extrapolation beyond known chemical space. For example, Martin *et al.* show in a series of papers how minimal models that capture the structural features of metal–organic frameworks can aid in interpreting the limits of materials optimisation.^31–33^ Similarly, Wolpert *et al.* used CG models of cages, treating them as hard-polyhedra, to map the phase space of their packing behaviour as a function of simple interactions.^34^ Evans *et al.* use a minimal model to explore placement and configuration effects on molecular motors in metal–organic frameworks.^35^ Using high-resolution models, Pesce *et al.* studied crowding-reactivity relationships in host-guest binding by artificially modifying model parameters.^36^

Here, we apply a bottom-up computational approach to build 1000s of minimal cage models using *stk*^37^ and *OpenMM*.^38^ This work does not represent a rigorous coarse-graining based on some experimental or higher resolution data but focuses on the role of a few building block parameters on the geometric stability and eventual cage structure of different topologies. We outline how this work could feed into future computational and experimental cage screening and decision-making. In particular, we use this model to efficiently explore parameter space and study the effect of distinct features, such as flexibility, which are difficult to isolate in atomistic models. We design the model and associated software towards automated rational design and we have made the outcomes of these predictions freely available online in an easily accessible manner for use by experimental researchers.

## 2 Computational methods

**Fig. 1:**
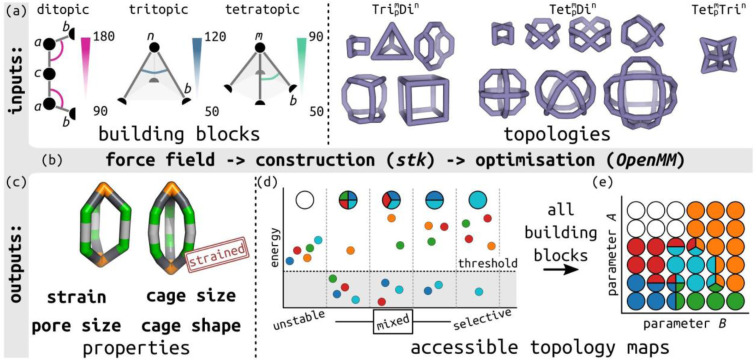
Outline of the minimalistic cage model. (a) Input to the model, including defining bond lengths and internal angles in ditopic, tritopic and tetratopic building blocks, and topology graphs. Bead types are provided in the figure and described in Table S2;† semicircles represent connections between building blocks. The main parameters of interest are highlighted: the internal ditopic angle (*bac*) and the two angles defining the tritopic (*bnb*) and tetratopic (*bmb*) building blocks. (b) Model construction and optimisation workflow built on *stk*, *OpenMM*, resulting in (c) the lowest energy conformer for a given input with a structure and properties. The two structures highlight the visual difference between low and high-energy structures. (d) Schematic of different self-sorting outcomes (unstable, mixed and selective) approximated in this work, where small circles correspond to different topologies, and (e) the mapping of those outcomes to a discrete, accessible topology map based on two general model parameters, *A* and *B*. The topology map shows points in phase space as circles, which are coloured by the topologies that are stable in those regions.

In this work, we define a minimalistic model to evaluate the accessible topologies in cage-like molecules, towards an understanding of their self-sorting behaviour, as a function of the key features of their constituent building blocks. This approach is informed by the design rules used by experimental chemists, which tend to be based on placing rigid building blocks on high-symmetry geometries.^39^ Our model evaluates the role of different building block angles in cage stability (of a series of defined topologies; Table S1†). We then use the relative cage stability of different topologies to approximate self-sorting outcomes. We focus on the role of internal building block angles, in particular, we evaluate the effect of changing the ditopic ligand bite-angle, which has been a key variable in designing MOC topologies,^40^ and pyramid angles of tritopic or tetratopic building blocks (including the planar case). The model's design is modular and general (not parameterised to any specific chemical system), so it can be applied to unknown systems. Fig. 1 summarises the minimalistic model and Fig. 1(a) shows the ligand model schematics, where the labels match the symbols in Table S2.† For comparison to previous cage design methods, we define the target bite angle of a ditopic ligand based on the two internal *bac* angles, as *θ*_bite_ = 2(*θ*_0_ − 90), where *θ*_0_ is the target *bac* angle on both sides. However, this only applies when the *baab* torsion is near 0°.

Using *stk* (https://github.com/lukasturcani/stk),^37^ we built linear, tritopic and tetratopic building blocks made up of three, four and five beads, respectively (Fig. 1(a)), where the beads have varying force field parameters (see below). We then place those building blocks on cage topologies^9^ in *stk* and perform a series of optimisation steps to attempt to get a single “lowest energy” conformer for analysis. The beads in a given system define the input parameters (target bond lengths and angles; bead property ranges are defined in Section S2†). By building and optimising a cage, which is defined by a topology and set of building blocks with specific beads (hence, specific input parameters), we are effectively testing the matching of those parameters to a topological constraint. High energy structures imply that some bond, angle or torsion term is far from their input targets.

The force field used for each model is based on bonding, angular, torsional and excluded volume terms, and has a potential energy form

1 *E* = *E*_bond_ + *E*_angle_ + *E*_torsion_ + *E*_excl.vol._,

where the functional forms of each term are2
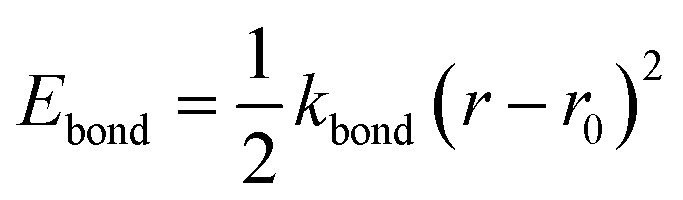
3
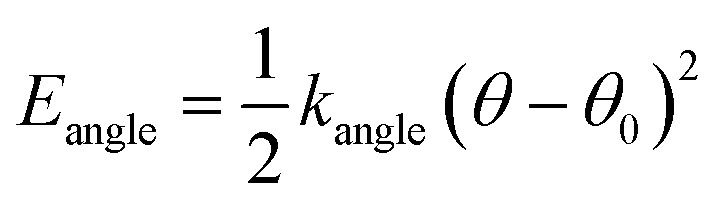

4 *E*_torsion_ = *k*_torsion_(1 + cos(*pφ* − *φ*_0_))

5
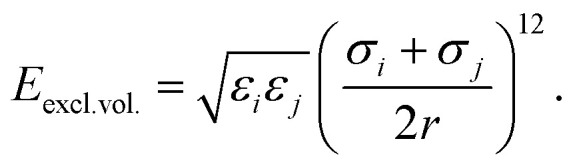
*r* is the bond length (or distance between two beads), *r*_0_ is the equilibrium bond length, *θ* is the angle, *θ*_0_ is the equilibrium angle, *φ* is the torsion, *φ*_0_ is the equilibrium phase offset, *p* = 1, *ε*_*i*_ is the strength of nonbonded interaction for bead *i* (in kJ mol^−1^) and *σ*_*i*_ is the size of bead *i* (in Å). *E*_excl.vol._ is a force term defined such that there is a penalty for bead overlap. When beads are connected by two or less bonds, the *E*_excl.vol._ term is turned off using the “bondCutoff” parameter. Table S2† defines the bead classes used and the ranges of their parameters. For bonds, *r*_0_ is determined through Lorentz–Berthelot mixing rules, *e.g.*, 
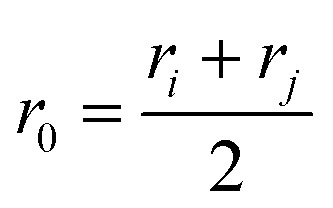
, where *r*_*i*_ and *r*_*j*_ are the equilibrium bond lengths assigned to bead types *i* and *j*, respectively. We have selected parameters such that the bond, angle and torsion terms are on the same scale as all-atom molecular force fields (when present) with *k*_bond_ = 1 × 10^5^ kJ molnm^−2^, *k*_angle_ = 1 × 10^2^ kJ molrad^−2^, *k*_torsion_ = 50 kJ mol^−1^. The rigidity in our system derives from the limited internal degrees of freedom in the cage building blocks. In this initial version of our model, we have used the same *ε*_*i*_ = 10 kJ mol^−1^ and *σ*_*i*_ = 1 Å for all beads. This model mimics a “good solvent” case, where solute–solvent interactions are energetically favourable. Therefore, this model assumes that cage collapse is a function of the geometric constraints of the building blocks, not inter-building block interactions or the hydrophobic effect. Section S2† contains details about handling the angles in tetratopic building blocks and how we define alchemical torsions.

We have applied a multi-step optimisation sequence to attempt to find the lowest energy conformer of each cage model. The sequence is made up of seven steps including a constrained geometry optimisation (constraints applied to bonds not formed during cage construction), a molecular dynamics simulation with softened bond and angle potential terms and optimisations of cage models after beads have been shifted away from the cage centroid (full details in Section S3†). These processes are designed to help exploration of conformational space. Each step uses *OpenMM* (inspired by other applications of *OpenMM* with CG models^41,42^) to perform local energy minimisation or molecular dynamics.^38^ This approach is similar to those we have applied on all-atomistic cage models.^9,25^ The optimisation sequence neglects the role of temperature in the accessibility of different conformers and searches for the lowest internal energy model. We expect the geometrical stability we compute in this paper and self-assembly outcome to depend on temperature, which we aim to consider in future models. Throughout, we directly compare the energy per number of building blocks in a cage (*E*_b_) as effective formation energies. The energy scale defined by the force field terms above is alchemical, and so we determine cage stability based on the data our model produces such that we can extract useful distinctions. The force field is modular, which we take advantage of herein, *e.g.*, to explore the role of a particular type of flexibility by modifying the torsional term in the ligand backbone (by design, this is the only torsional term present). By default, we explore the most constrained case with the torsion restriction “on” (*i.e.*, preorganised building blocks), where the two ditopic binding sites face the same direction, but in Section S3.3,† we explore how topology accessibility changes without this constraint.

## 3 Results and discussion

We generated 2890 cages in our cage space from a pool of 287 distinct building block combinations and 13 topologies, with and without torsion restriction. We explored the effect of cage topology, building block coordination number, building block flexibility and target internal angles of the two building blocks on topology accessibility relationships and approximations to self-sorting behaviour (Fig. 1). Note that the cage space is discretised over the target internal angle values, which are shown in all plots. We implemented an optimisation sequence, described in Section S3,† that is reasonably robust, but does result in some instabilities in the phase space (Section S5†). However, we show that the optimised cage models have structural parameters that match the input force field parameters and that most of the strain exists in the angle terms (Section S4†).

### 3.1 Effect of building block angles on topology stability

**Fig. 2:**
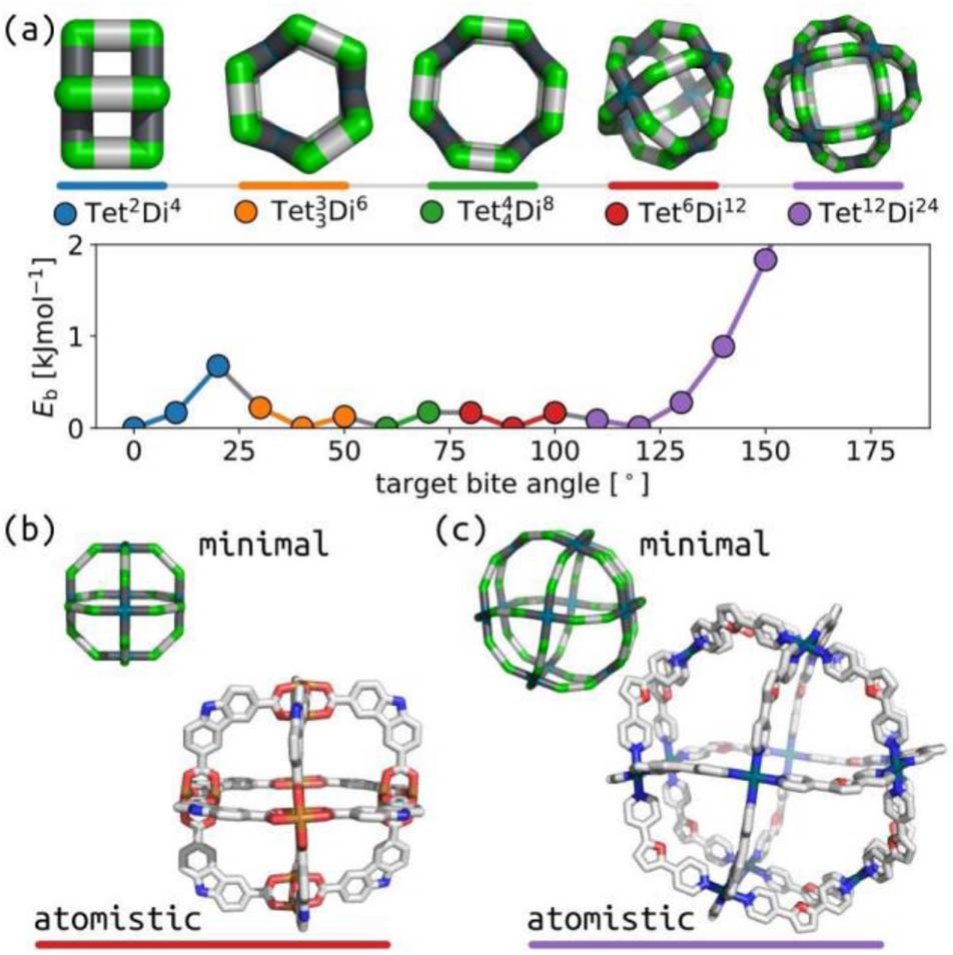
(a) Relationship between ditopic bite-angle and topology preference for square-planar tetratopic building blocks with torsion restrictions. The lowest energy structure for each selected topology is shown; horizontal bars are colour-coordinated to the points in the plot. Green are the *a* beads, cyan are the *m* beads, black are the *b* beads, and grey are the *c* beads. (b) Comparison of minimal model of **Tet**^**6**^**Di**^**12**^ with target bite angle of 90° with a crystal structure (CCDC: SUPPID).^45^ (c) Comparison of minimal model of **Tet**^**12**^**Di**^**24**^ with target bite angle of 120° with a crystal structure (CCDC: BIMXIF).^46^ The minimal models are minimum energy points in (a) with the same colour as the line at the bottom. In atomistic models, grey are carbon, blue are nitrogen, red are oxygen, cyan are palladium, hydrogens and solvent are not shown for clarity.

Over the last twenty years, the relationship between self-sorting outcome and ligand internal angles in square-planar Pd(ii)-based systems has driven the design of impressive cage systems, including heteroleptic cages (through shape complementarity)^43,44^ and huge Pd_*x*_L_2*x*_ cages.^40^Fig. 2(a) shows that our model produces this behaviour, with the expected topology being stable at the appropriate bite-angle. Fig. 2(b) and (c) show geometrical agreement between stable structures with square-planar tetratopic building blocks in topologies **Tet**^**6**^**Di**^**12**^ (target bite angle of 90°) and **Tet**^**12**^**Di**^**24**^ (target bite angle of 120°) with experimental crystal structures.^45,46^ This also highlights the chemical generality of our model, where agreement is achieved for square-planar Pd(ii) systems and the geometrically equivalent Cu paddle-wheel in (b).

**Fig. 3:**
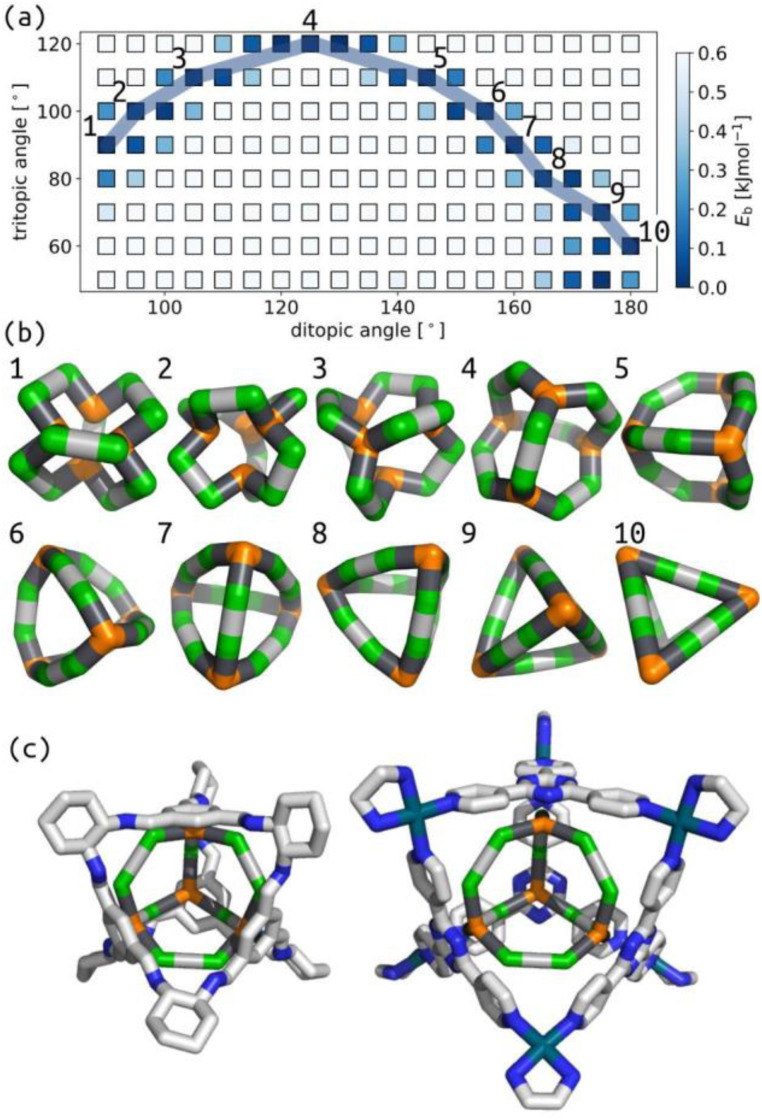
(a) Relationship between target tritopic angle, target ditopic internal angle and energy for **Tri**^**4**^**Di**^**6**^ topology; the colour map is *E*_b_. White squares show high-energy points. (b) Example low energy structures along the blue line in (b) with varying ditopic and tritopic angles. Green are the *a* beads, orange are the *n* beads, black are the *b* beads, and grey are the *c* beads. (c) Overlap of a porous organic cage (CCDC code: FOXLAG^47^) and a metal–organic cage (CCDC code: TIXFIP^50^) with two **Tri**^**4**^**Di**^**6**^ models from our phase space with tritopic angles of 120° and ditopic angles of 125° and 135°, respectively. In atomistic models, grey are carbon, blue are nitrogen, cyan are palladium, hydrogens and solvent are not shown for clarity.

Similarly, we show that our model produces structures akin to those expected for the **Tri**^**4**^**Di**^**6**^ topology based on tetrahedral MOCs and POCs.^47–49^Fig. 3(a) summarises the relationship between tritopic and ditopic building block angles and cage stability for the **Tri**^**4**^**Di**^**6**^ topology. The data shows that the stable regions shift towards 180° in internal ditopic angle as the tritopic building block becomes less planar, exemplified by structures in Fig. 3(b). This outcome, again, is consistent with the geometry of a tetrahedron. We show in Fig. 3(c) the structural similarity between two models from our phase space (differing by input ditopic angle) to known experimental structures. The two low-energy models have internal ditopic angles approximately matched to the atomistic building blocks of the experimental structures. What is less obvious about the data in Fig. 3 is why there are stable points at low internal angles for smaller tritopic angles (structures 1–3 in Fig. 3(b)). We posit that these structures are geometrically stable in the framework of this model, but may be difficult to realise using real chemical structures. However, this highlights new geometries that we could target in future design studies based on our models.

We have explored the relationship between internal building block angles and cage internal energy for all topologies in this work (Section S6 and S7†). The number of stable regions (or wells) and the smoothness in these angle maps tends to increase as the degrees of freedom increase (*i.e.*, for larger topologies). Although, some of the larger topologies do not have this effect. Also, some topologies show two stable wells for a given pyramid angle, for example, at tritopic angle 110° in Fig. 3(a), that deviate from the single well for the planar tritopic or tetratopic case. Visualising structures in these types of surfaces, we see structures with inverted tetratopic or tritopic building blocks as in Fig. 3(b) 1–3. For other topologies, we see that there are low energy structures up until some critical internal ditopic angle that leads to instability (Fig. S16 and S20†). This shows that there are topological effects determining the energy surface associated with these two types of internal angles. The observed topological dependence is non-trivial and may be related to some inherent flexibility. For example, studies have shown that the **Tri**^**4**^_**2**_**Di**^**6**^ topology results in flexible cages^36^ and here the topology shows a flat energy surface, while **Tri**^**4**^**Di**^**6**^ has the double-well behaviour and tends to result in more rigid cage structures.^51^ However, studies on POCs show that there is flexibility in the window apertures of **Tri**^**4**^**Di**^**6**^ cages.^52^

Given the geometrical nature of our approach, it is promising that our models agree with literature examples, *e.g.*, from ref. 53 and their findings of stable angle-topology combinations in metal–organic polyhedra (they performed a detailed review of the angles in building block pairs and their eventual topology). In particular, we find overlapping stable regions for the **Tri**^**4**^**Di**^**6**^, **Tet**^**6**^**Di**^**12**^, **Tri**^**8**^**Di**^**12**^, **Tet**^**6**^**Tri**^**8**^, and **Tet**^**12**^**Di**^**24**^ topologies (all topologies shown in Fig. S1†). We note that for **Tet**^**12**^**Di**^**24**^, they also found another stable structure, but the formation of this structure involves a decrease in symmetry of the angles around the tetratopic building blocks, which is not studied in our model. Therefore, we would not expect to find that stable configuration. Similarly, we support the findings in ref. 10 for our studied topologies (**Tri**^**2**^**Di**^**3**^, **Tet**^**2**^**Di**^**4**^, **Tri**^**4**^**Di**^**6**^, **Tet**^**6**^**Di**^**12**^, **Tri**^**8**^**Di**^**12**^, **Tet**^**6**^**Tri**^**8**^). In ref. 10, they point out how concerted rotations within building blocks play a significant role, which our model will not currently capture due to its simplicity. However, the granularity of our approach begins to highlight how tuning the force field may better explain transitions between angles in our space. Additionally, there is an interesting question about the degree of deviation that is possible from existing design rules, and if we can control for or predict that leniency.

An interesting case is the **Tet**^**4**^_**2**_**Di**^**8**^ topology, termed a double-walled tetrahedron.^28,54–56^ Using a consistent energy scale, there are no stable structures in the whole angle space (see details in Fig. S33†), and further analysis shows that there is a systematic strain in the structure, which we propose to be due to the required twist in the ditopic building blocks bridging some of the tetratopic sites (this can be seen in known experimental structures of Pd(ii) cages in this topology). Therefore, the torsion constraint at 0° leads to strained structures, although the cage models look reasonable visually. While we are not aiming to develop an exhaustive model in this manuscript, this type of outcome poses interesting questions for our approach. Can we find the building block features that lead to well-defined stable regions in the angle space of a specific topology? Are the flexibility in the bonds and angles, or the torsion restriction important factors? The requirement of non-zero torsions is present for **Tet**^**8**^**Di**^**16**^ also. Young *et al.* highlight cases of torsion restrictions with different target angles,^10^ and how they result in cages with symmetric structures. This issue is also exemplified for the **Tri**^**6**^**Di**^**9**^ trigonal prism structure, which, by definition, requires a tritopic angle of 60° and 90° for an ideal trigonal prism structure. Hence, increased flexibility in the tritopic angle term would likely modify the accessible cage space for this topology.

### 3.2 Mapping accessible topologies and their selectivity

Next, we distil a large cage space into what we term “accessible topology maps”. These figures map building block parameters to their calculated accessibility for all studied cage topologies. The outcomes of self-assembly are governed by experimental conditions, thermodynamics (*i.e.*, free energy) and kinetics, which our model does not consider. However, the accessible topology maps, based on internal cage energies, are an approximation to this problem based on what topologies are permitted (or not) for given building block parameters. Under this model, if only a single species is stable at a given point in phase space, we assume that self-sorting will occur and produce only that species. Fig. 1(d) shows the different accessibility outcomes (all unstable, mixed or selected), and how that can be used to map cage space, where *A* and *B* in this work are the target tritopic/tetratopic pyramid angles and target internal ditopic angles.

**Fig. 4:**
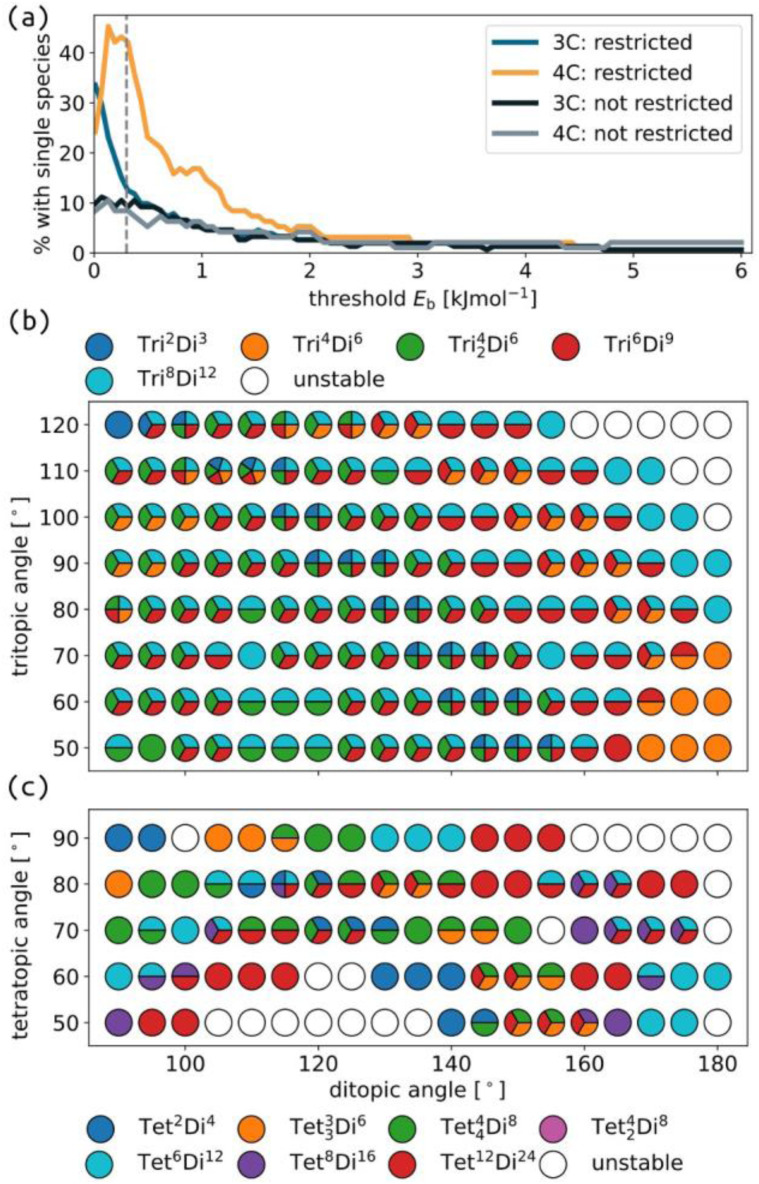
(a) Percentage of selected topologies (*i.e.*, only one topology is stable for a given building block combination) as a function of the threshold *E*_b_ for accessibility for cages formed from ditopic and either tritopic (3C) or tetratopic (4C) building blocks, with and without restricted torsions, described further in Section S3.3.† Accessible topology maps for cages formed with torsion restrictions, ditopic building blocks and (b) tritopic or (c) tetratopic building blocks. The colour map for each sub-figure is shown in the corresponding legend.

To compute accessible topology maps, we must define an energy per building block (*E*_b_) value below which a structure is deemed accessible. Because our force field does not correspond to any specific experimentally relevant system, we chose this parameter based on the threshold that provided the maximum degree of self-sorting (Fig. 4(a)); the selected value is 0.3 kJ mol^−1^. Note that this maximum occurs for 4-connected structures (4C), while the maximum is at lower *E*_b_ for 3-connected structures (3C). This model focuses on geometrical stability, ignoring many other factors (*e.g.*, dispersion interactions, sterics, computation methodology, cage properties and solvent) that ultimately affect the appropriate choice of this threshold in any computational attempt to predict self-sorting; *i.e.* 0.3 kJ mol^−1^ is entirely model dependent.

Fig. 4 clearly shows different degrees of self-sorting for topologies with 3-connected and 4-connected building blocks. This is summarised clearly in Fig. 4(a), where the percentage of selected systems is higher for 4-connected cages than 3-connected cages (under the rigid regime) and remains significant up to higher energy thresholds. It is possible that including all enumerated topologies, as in the work by Poole *et al.* may alter this behaviour.^18^ Fig. S43† shows that the number of unstable cages is higher for the 4-connected topologies than the 3-connected topologies. This outcome, from our very simple model, suggests that 3-connected topologies, at least with symmetric tritopic building blocks, will suffer from more competition during self-assembly than the 4-connected topologies. However, there are many examples where our model predicts mixing, but a single species forms experimentally (see discussion of **Tri**^**4**^**Di**^**6**^ and **Tri**^**4**^_**2**_**Di**^**6**^ above). We suggest that cage topologies could be selected based on such data to avoid issues with sensitivity to experimental conditions. We also show that the self-sorting propensity depends on the pyramidal angles of the tritopic or tetratopic building block, where planar building blocks lead to more selected isomers at higher energy thresholds (Fig. S42†). Focusing on the smallest, stable topology, Fig. S44 and S45† show regions of stability for most topologies, where the largest topologies tend to be favoured at larger internal angles. Therefore, it is possible to design for a particular cage topology if desired.

### 3.3 Preorganisation effect on topology accessibility

**Fig. 5:**
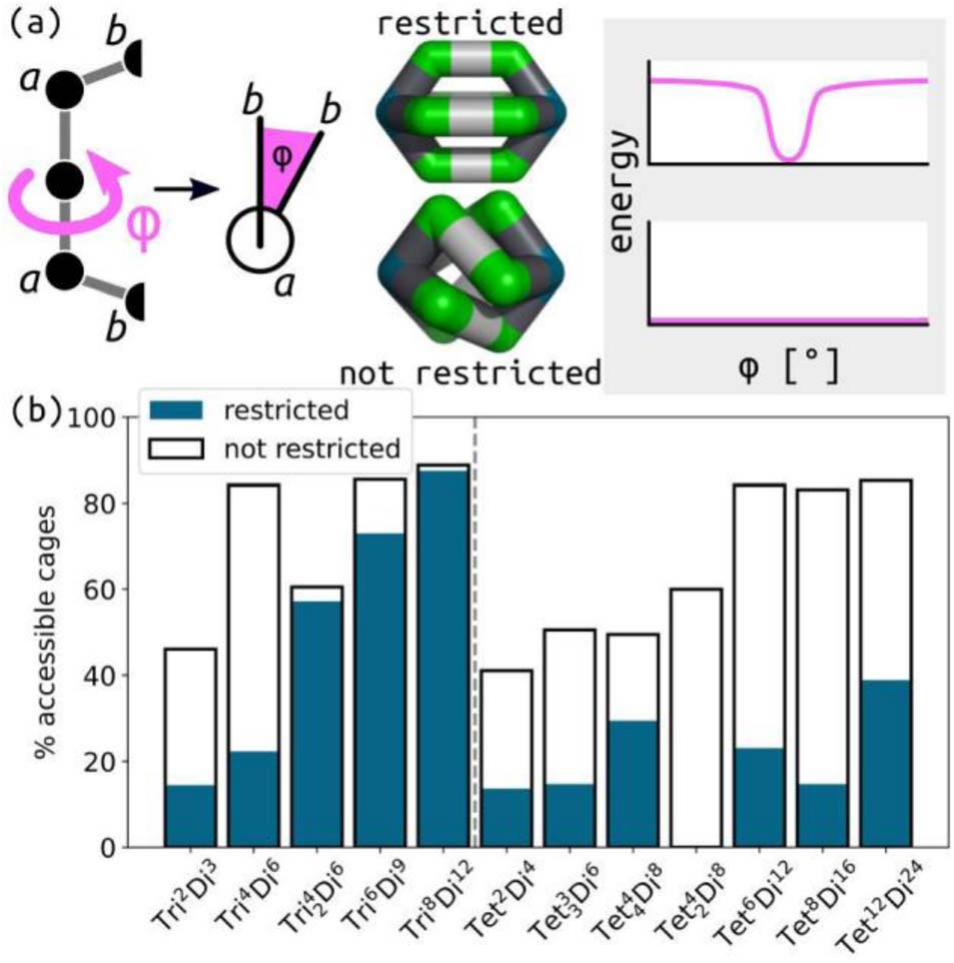
(a) Schematic of the preorganisation modified in this work, where the *baab* torsion (*φ*; Newmann projection is shown) is either restricted (top; rigid) or not restricted (bottom; flexible). Representations of **Tet**^**2**^**Di**^**4**^ structures and the torsion force field term in both cases are shown. (b) The effect of this restriction on the proportion of accessible structures for each topology. The difference in the blue and white bins represents the effect of flexibility.

So far, we have enforced preorganisation on the ditopic building blocks, where the binding beads are forced to face the same direction with a restricted torsion along the ligand backbone (see Section S2† for more information). This is one way in which these building blocks could be preorganised, where the rigid, well-defined binding site orientation facilitates self-assembly processes and allows the application of predictive geometrical rules. Chemically speaking, this corresponds to rigid building blocks with few rotatable bonds, resulting in a constant relative orientation of the reactive bonding atoms. Increased flexibility of the building blocks or the cages makes computational structure prediction more challenging for atomistic simulations/calculations due to the increased degrees of freedom and the likelihood of multiple low-energy states to consider. Importantly, small deviations from the ideal geometries of rigid building blocks can lead to changes in self-assembly outcomes.^10,57^ Therefore, we aim to use our toy model to explore the impact of building block preorganisation (at least in one form) by switching off this torsion restriction (Fig. 5(a)). This will inherently lead to more flexible cages, which we assume will always increase the accessibility of building block and topology combinations under the conditions of this model.

Regarding selection, Fig. 4(a) shows that the percentage of selected cage structures decreases significantly when preorganisation is switched off. A geometrical effect of removing this torsion restriction on the cage structures is shown in Fig. 5(a), where helicity occurs without torsion restrictions, which is akin to recent work in Pd_2_L_4_ cages.^58^ Further, we see that there would be a mixed effect of turning off torsion restrictions on pore size; many unrestricted cages are collapsed, but the same seems to be true for unstable restricted cages with torsion restrictions. Shape persistence or porosity is not directly related to flexibility, although the average pore size^52^ should be considered for flexible cages, rather than a static image, which provides a less clear image of cage porosity.

For small ditopic internal angles, removing the torsion restriction has a strong impact, resulting in many low energy cages in the angle map (Fig. S37†). This results in more mixing in the accessible topology maps (Section S10†) than in the restricted case. Focusing on the smallest accessible topology at each point, the topology maps show that the preferred topology shifts from the smallest to the largest topology as the internal angles increase with unstable regions in the top-right-hand corner. However, this is not the case when preorganisation is on. Further, different topologies are preferred (as the smallest stable structure) with and without torsion restrictions for a given pair of building block pairs. There are regions that were unstable or favoured larger topologies, that now favour smaller topologies when preorganisation is not present. As expected for such a minimal model, the effect of preorganisation is negligible at higher internal angles, where the torsion has a smaller effect.

Fig. 5(b) summarises the change in the proportion of accessible structures with and without restricted torsions. The difference between topologies with 3-connected and 4-connected building blocks is interesting; the effect of preorganisation on topology stability is much more variable for 3-connected topologies. However, this could be a feature of the studied topologies, not the connectivity. For example, the **Tet**^**4**^_**2**_**Di**^**8**^ topology ignores this trend (all restricted cages are deemed unstable in our model; see above). The change in accessibility of a given topology due to flexibility (the gap between white and blue bars in Fig. 5(b), larger gaps suggest larger effect of flexibility) may be a useful indicator of the degree of difficulty of the rational design of a certain topology with flexible components. Our model does not consider the free energy or varying flexibility within cage bonds and angles, all of which would significantly impact the self-sorting of flexible structures. Overall, the role of flexibility will diminish the effectiveness of geometry-based design principles, and our data shows that it increases the competition between different cage topologies. However, it is not immediately clear whether these results are false positives due to the model's simplicity. Regardless, simple models such as this can begin to uncover where and how to introduce flexibility in the most useful and robust way, avoiding the pitfalls of challenging synthetic outcomes and optimising materials properties.^59,60^

### 3.4 Relationship between building blocks, shape and topology

The shape of cage molecules determines the properties of their intrinsic pores and influences their packing behaviour.^34,49^ As defined,^9,10^ the topology of a cage molecule does not necessarily provide information on the eventual cage geometry. Here, we systematically mapped the relationship between topology, shape and building block parameters. We calculate a series of shape measures for a given cage structure, which are the deviation of that cage from ideal shapes, using the SHAPE v2.1 software.^61^ We calculate the shape only for some topologies (shapes with vertex numbers 3 to 8 to avoid the cost of 12-vertex calculations), and we do so on the centroids of either the tritopic/tetratopic building blocks or the ditopic building blocks in the cage structure. For example, for the **Tri**^**4**^**Di**^**6**^ topology, we calculate the shapes based on the positions of the four tritopic building blocks (compared to four-vertex shapes, *e.g.*, square and tetrahedron) and based on the positions of the six ditopic building blocks (compared to six-vertex shapes, *e.g.*, octahedron). Therefore, some topologies will have two shape measures (more detail in Section S11†). Table S3† shows the ideal shape of each topology, where the naming convention states the geometry and the number of vertices in the shape deviation measure. Importantly, these deviation measures are unitless and allow quantitative comparison between different shapes.

**Fig. 6:**
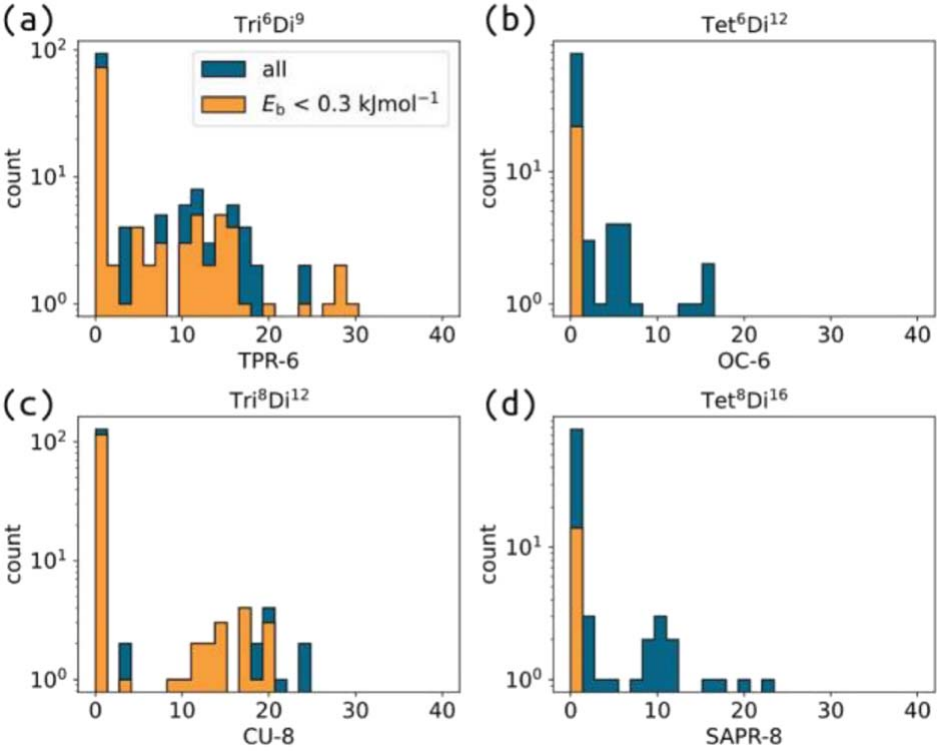
Distribution of the deviation from the ideal shapes for (a) **Tri**^**6**^**Di**^**9**^ (shape measure: TPR-6), (b) **Tet**^**6**^**Di**^**12**^ (shape measure: OC-6), (c) **Tri**^**8**^**Di**^**12**^ (shape measure: CU-8) and (d) **Tet**^**8**^**Di**^**16**^ (shape measure: SAPR-8) with torsion-restrictions for tritopic or tetratopic building blocks. Blue distributions are all cages, and orange is for stable cages. This data is on a log scale due to the high proportion of cages near zero.

Analysing the data, we see that there are nearly no deviations from shape values of zero for small topologies, indicating a symmetry in the ditopic building block positions regardless of the molecular changes seen in Fig. 5(a). The flexibility of larger topologies also allows for the stability of structures far from their ideal shape. However, we see that deviations from the ideal shape do not necessarily become more common for larger topologies. For example, the distribution of **Tet**^**6**^**Di**^**12**^ shapes in this data set are thinner than **Tri**^**6**^**Di**^**9**^ (Fig. 6 also shows the comparison of **Tri**^**8**^**Di**^**12**^ and **Tet**^**8**^**Di**^**16**^). For the flexible **Tri**^**4**^_**2**_**Di**^**6**^,^36^ we see a significant deviation in the tritopic and ditopic building block shapes for stable structures. And removing torsion restrictions allows for these structures to be low energy, which is the case for other topologies (including the more rigid, **Tri**^**4**^**Di**^**6**^). However, this result could suggest the generation of nonsensical “stable” cages with unrealistic shapes. For flexible systems, the distribution of shapes at equilibrium is likely the more important feature and something we can extract for low cost from this model.

This analysis suggests that, under the design of this model, a stable cage in the rigid regime will likely result in a shape near the expected ideal shape of that topology. And that this result becomes less robust for certain topologies or larger topologies. This may be a bias from the design of the problem. Our interest here is how available low-energy structures with controllable shapes are. For example, the shape maps for **Tet**^**6**^**Di**^**12**^ and **Tri**^**8**^**Di**^**12**^ tell very different stories about the shape diversity over their stable structures. For the application of binding/separating isotropic guests, an ideal (symmetric) shape is useful. However, precisely controlled binding environments are often asymmetric, and controlling the cage and pore's shape (and dynamics) is critical to designing enzyme-like hosts. Therefore, understanding the relationship between cage inputs to eventual shape is crucial.

### 3.5 Beyond ditopic building blocks

**Fig. 7:**
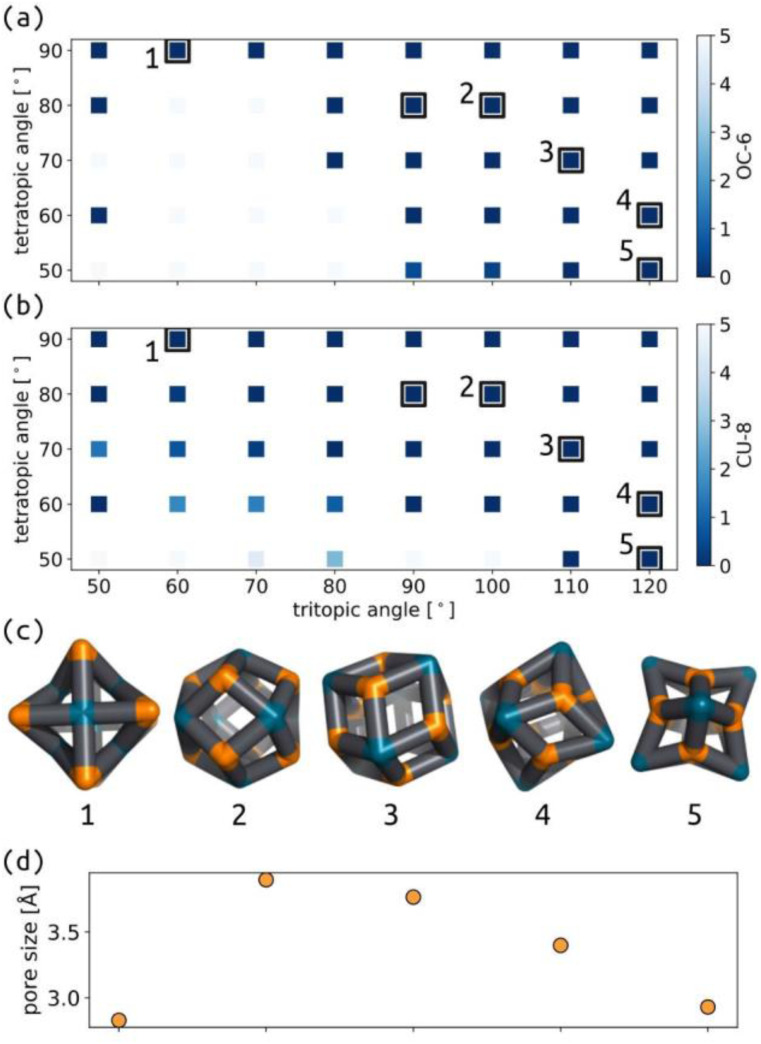
Map of (a) OC-6 shape and (b) CU-8 shape for the tetratopic and tritopic building blocks in the **Tet**^**6**^**Tri**^**8**^ topology. The colour map shows the deviation from the ideal shapes. Black squares highlight stable cage structures. (c) Low energy structure examples. (d) Approximate pore radius of structures 1–5. Orange are the *n* beads, cyan are the *m* beads, and black are the *b* beads.

We applied the same protocol as above to the common **Tet**^**6**^**Tri**^**8**^ topology, which switches between two main geometries as a function of the tritopic and tetratopic angle (Fig. 7 and S38†). These data agree with the geometrical analysis performed by Tranchemontagne *et al.* regarding this topology.^53^ Their analysis highlights the range of deviations that occur in real molecular structures, due to flexibility in bonded interactions, for example. Comparing their analysis to the width of stability shown in our model suggests that we are considering more rigid connections than the experimental systems. Low energy structures for this topology have good shape similarity to octahedral (OC-6) and cubic (CU-8) measures, depending on whether it is measured from the tetratopic or tritopic building blocks, respectively. However, Fig. 7(c) shows that the overall cage geometry changes significantly; therefore, measuring the shape of cages based on decoupled building block positions may be limited. The distinction between 80° and 90° tetratopic building blocks is stark, with regards to the stable geometries and pore size, which suggests this topology may be ripe for controlled adaptability, where small changes in building block preference (through external stimuli, for example) could result in large changes in cage configuration.

### 3.6 Application to future screening

Here, we aim to highlight the utility of this model and the next directions we can take. Immediately, it is clear that our model only provides a geometrical understanding of topology stability, and will not capture the changes in self-assembly outcomes that have been studied experimentally (*e.g.*, how metal identity may change the kinetics).^57^ In particular, our model, based only on connectivity, is expected to provide multiple solutions approximating what could be expected during the self-assembly process. However, there is an insight to gain from these solutions, especially if the experimental and model data disagree. Through this oversimplification, we can begin to pinpoint the dominant factors in many different self-assembly outcomes.

One particularly interesting use of these models, and our previous CG models of 2D covalent organic frameworks,^62^ is to provide more realistic initial structures for atomistic model construction, through software like *stk*. Recently, Kondinski *et al.*^63^ performed knowledge engineering of experimental data to develop an algorithm for rational MOC design, and efficient atomistic model generation. Their approach is akin to ours, where they build up an understanding of which building blocks will be stable in which topologies based on extracted experimental data. Better input models can help avoid costly geometry optimisations at the atomistic level by improving initial guesses and simplifying computational workflows.

The data produced in this work, accessible topology maps and images of self-sorting outcomes are provided through an open interface at https://andrewtarzia.github.io/selfsort/ (we discuss its usage in Section S13†). This allows the mapping from building block parameters to the accessible topologies for screening. As the current model does not include different ligand sizes, only so much property screening can be performed. However, we discuss the property space of our models in Section S12.†

## 4 Conclusions

In this work, we have introduced an approach to generating and analysing minimal cage-molecule models for their exploration and design. Our minimal model, which contains few parameters, offers more efficient access to new insights than traditional techniques by mapping the underlying relationships between cage design parameters. We systematically plot the accessible topology space based on these parameters with and without ditopic ligand preorganisation, where increased flexibility tends to result in a drastic increase in stable structures. Importantly, we find good agreement between our model and existing experimental data available for a series of topologies of porous organic and metal–organic cages, where we found that the reported topology is stable in our model for the same building block angles (Section 3.1). And in the cases where our model does not agree with previous work, we can highlight the structural features causing this outcome, highlighting potential new areas for design in cage molecules. Therefore, the minimal model we have introduced, which is extendable to other molecules and materials, could provide guidance in design problems and initial property predictions for high-throughput screening; this type of approach has shown promise in metal–organic framework design.^31–33,59^

Our model opens up interesting questions about the role of dynamics in controlled self-assembly; the balance between achieving stability and control over the outcome of self-assembly is not trivial. We find that increased flexibility leads to more stable cage topologies, resulting in an increased competition. However, this is an observation of this model and the role of flexibility in real systems is multifaceted and in need of detailed study. Low-cost computational tools can be used to approach the prediction and design of less trivial cases involving larger, more flexible, or unsymmetrical ligands that are near-intractable to study atomistically on large scales.^23,25,28^ We propose that this will lead to much more efficient screening and design of cage molecules as, for example, a step in approaches similar to that by Young *et al.*^10^ Our software (based on *stk* and *OpenMM*) is extendable to many other cage types and materials, and is open-sourced (https://github.com/andrewtarzia/CGExplore), which facilitates future work. We expect that our work could be integrated with automatic optimisation^64^ and enhanced sampling^36,51^ into a platform for the rational design of cages based on shape, flexibility and porosity.

We have limited this work to symmetrical building blocks of a single size, with no control for stereochemistry, no consideration of intermolecular interactions between components (which can strongly impact cage formation), and no solvation or ions. In particular, while this work includes topologies of cages formed by multi-dentate building block interactions (*e.g.*, subcomponent self-assembly), this iteration does not include the necessary detail at the metal centres to properly encode the twist and stereochemistry present in these MOCs.^65^ We do not model the actual self-assembly process, which is likely necessary to predict self-sorting outcomes generally, and our approach will likely result in false positives. Additionally, it is expected that many self-sorting outcomes can be overcome by choice of experimental conditions. The effect and degree of flexibility in cage structures are likely to depend on the solvent and environment. Further, the nature of kinetic traps and stable intermediates in cage formation is not trivial to understand. Including higher resolution CG models (*e.g.*, the Martini force field^66^) may help overcome inconsistencies in the phase space, especially for larger molecules. Additionally, the existing models using the Martini force field for solvent molecules, for example, will help to introduce the effect of solvent in cage formation. Indeed, to study interesting phenomena such as stability cliffs in the cage phase space, we require higher CG resolution. Understanding these sharp changes in stability or properties over a phase space, and whether they are topological effects, could be useful in controlling self-assembly in complex cage structures.

## Data availability

The code for structure generation and analysis can be found at https://github.com/andrewtarzia/CGExplore and on Zenodo at https://doi.org/10.5281/zenodo.8184816. Data for this paper, including structure files and properties, are available at Zenodo at https://doi.org/10.5281/zenodo.8136345. Topology maps and structure images are available at https://andrewtarzia.github.io/selfsort/.

## Author contributions

A. T.: conceptualisation, data curation, formal analysis, funding acquisition, methodology, software, visualisation, writing – original draft, reviewing and editing. E. H. W.: conceptualisation, writing – reviewing and editing, formal analysis. K. E. J.: writing – reviewing and editing, funding acquisition, supervision. G. M. P.: conceptualisation, writing – reviewing and editing, funding acquisition, supervision.

## Conflicts of interest

There are no conflicts of interest to declare.

## Supplementary Material

SC-014-D3SC03991A-s001
