# Supplementary material for: Systematic exploration of accessible topologies of cage molecules via minimalistic models
Source: Chem Sci. 2023 Oct 11;14(44):12506–17. doi: 10.1039/d3sc03991a (PMC10646940; doi:10.1039/d3sc03991a)
Supplement: SC-014-D3SC03991A-s001 [file SC-014-D3SC03991A-s001.pdf]

Electronic Supplementary Material (ESI) for Chemical Science.
This journal is © The Royal Society of Chemistry 2023

Supplementary Information for

Systematic exploration of accessible topologies of cage molecules via minimalistic models

Andrew Tarzia [a], **Emma H. Wolpert** [b], **Kim E. Jelfs** [b], **and Giovanni M. Pavan** [a,c]

* *E-Mail: andrew.tarzia@polito.it, giovanni.pavan@polito.it*

S1. Topology definitions

Throughout this work, we use the nomenclature from Ref. S1, where a topology has a general form $\mathbf{X}_p^m\mathbf{Y}^n$. We define the topologies used in Table S1. **X** and **Y** are two distinct building blocks in the cage, defined by their connectivity as either **Di**, **Tri** or **Tet** for ditopic, tritopic and tetratopic, respectively. **X** should always be the higher connectivity building block of the two. *m* and *n* denote how many of each building block there is in the topology. If an **X** building block is connected to another **X** building block through only one **Y** building block, then *p* is not shown. Otherwise, *p* is the number of **Y** building blocks connecting two **X** building blocks.

Table S1: Definition of topologies studied in this work and stoichiometry (in terms of the number of constituent building blocks). * suggests a modification on the named *stk* class was performed or that the topology was defined from scratch for this paper; these definitions are in the code provided with this paper.

topology	*stk* [S2] class	num. building blocks
$\mathbf{Tri^{2}Di^{3}}$	TwoPlusThree	5
$\mathbf{Tri^{4}Di^{6}}$	FourPlusSix	10
$\mathbf{Tri_{2}^{4}Di^{6}}$	FourPlusSix2	10
$\mathbf{Tri^{6}Di^{9}}$	SixPlusNine	15
$\mathbf{Tri^{8}Di^{12}}$	EightPlusTwelve	20
$\mathbf{Tet^{2}Di^{4}}$	M2L4Lantern	6
$\mathbf{Tet_{3}^{3}Di^{6}}$	M3L6	9
$\mathbf{Tet_{4}^{4}Di^{8}}$	M4L8*	12
$\mathbf{Tet_{2}^{4}Di^{8}}$	-*	12
$\mathbf{Tet^{6}Di^{12}}$	M6L12Cube	18
$\mathbf{Tet^{8}Di^{16}}$	EightPlusSixteen	24
$\mathbf{Tet^{12}Di^{24}}$	M12L24*	36
$\mathbf{Tet^{6}Tri^{8}}$	SixPlusEight	14

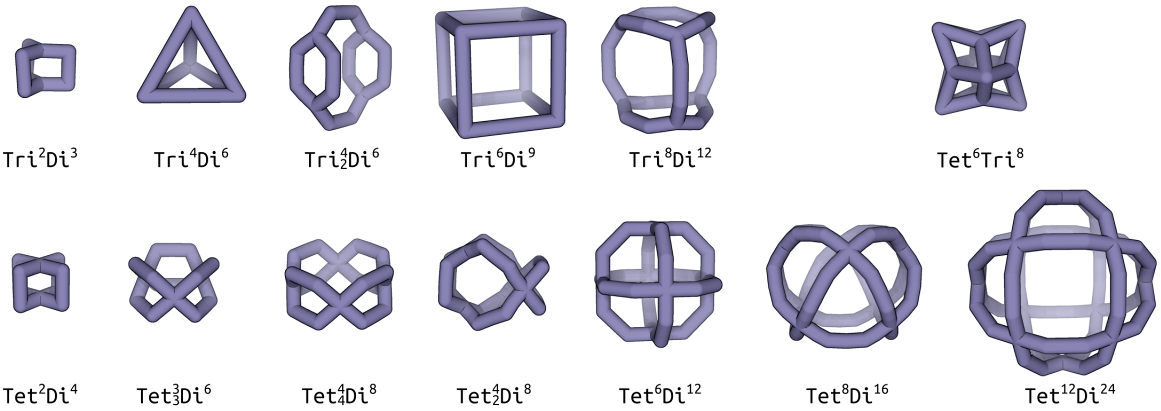

Fig. S1: Visual representation of each topology.

[a]Department of Applied Science and Technology, Politecnico di Torino, Corso Duca degli Abruzzi 24, 10129 Torino, Italy
[b]Department of Chemistry, Molecular Sciences Research Hub, Imperial College London, White City Campus, Wood Lane, London, W12 0BZ, UK
[c]Department of Innovative Technologies, University of Applied Sciences and Arts of Southern Switzerland, Lugano-Viganello 6962, Switzerland

S2. Bead and force field definitions

Table S2: The connectivity and parameter ranges of beads used in this work.

bead class (symbol)	connectivity	r_i range [Å]	θ_0 range [degrees]
2-connected cores (c)	2	2	180
arms (a)	2	1	90 to 180 in increments of 5
binders (b)	2	1	180
3-connected cores (n)	3	2	50 to 120 in increments of 10
4-connected cores (m)	4	2	50 to 90 in increments of 10

The role of the connectivity parameter is to handle the appropriate definition of angular terms in the four-connected nodes, where target angles between neighbouring beads in the pyramid are θ_0, but the target angle between beads on the opposing side of the central atom, $\theta_{\mathrm{opposite}}$, is defined as

$$\theta_{\mathrm{opposite}} = 2\arcsin\left(\frac{\sin\theta_0}{4\sqrt{2}}\right). \quad \text{[S1]}$$

This definition removes the possibility of rearrangements of the bonding pattern on these building blocks.

In this work, we implement an artificial torsion (technically defined over five atoms) that mimics a form of rigidity relevant to cage molecules; all other torsions are ignored. The torsion of interest is between $baab$ beads (where the connectivity is $bacab$, *i.e.*, the c bead is ignored), which defines the alignment of the binding atoms of the ditopic linkers. This torsion is set to 0° (using a phase offset, $\phi_0 = 180°$), or it is off, allowing free rotation.

S3. Optimisation sequence

The optimisation sequence is as follows

1. Starting from geometry-optimised building block structures, we construct the cage with *stk*.
2. A constrained geometry optimisation is performed for 20 steps, where all bond and angle potential terms are softened by a factor of 10, all torsions are switched off, and constraints are applied to all atoms not part of bonds formed by *stk*.
3. A geometry optimisation is performed with the full force field applied and no constraints.
4. A series of model conformers are generated, where all instances of a single bead type are displaced away from the model's centroid by 1 Å, 2 Å, 3 Å or 4 Å. Each generated conformer is then geometry optimised. This is repeated for each distinct bead type.
5. Lowest energy structures of models with adjacent input parameters (*e.g.*, the previous target ditopic internal angle) are collated and geometry optimised with the new force field parameters.
6. A MD simulation is performed starting from the lowest energy conformer accessed so far, where the bond and angle terms are softened by a factor of 10 and all torsions are switched off, allowing for conformer-space exploration. The simulation is performed in the NVT ensemble with Langevin dynamics for 20000 steps (collecting a conformer every 500 steps, 40 in total) with a friction coefficient of 1 ps^{-1} and a time step of 0.5 fs at 300 K. All bead masses were set to 10 a m u , which are small for the average building block used in realistic cage systems. Every extracted conformer is then geometry optimised with the full force field.
7. The lowest energy conformer accessed in this process is saved as the final conformer of the model.

The sequence above was developed through trial and error to maximise how consistently the lowest energy conformer was found over our cage space. We believe that the *ad-hoc* force field applied in this work has led to a difficult-to-traverse potential energy surface, which we have overcome through multiple approaches (*e.g.*, applying bead translations, the soft MD step). A more robust global optimisation sequence is our goal for future work.

We show in Fig. S2 that the energies extracted from two distinct runs of this workflow for three topologies (**Tri^{4}Di^{6}**, **Tri$^{4}_{2}$Di^{6}** and **Tri^{8}Di^{12}**) are mostly equivalent. However, there are some examples of changes in energy larger than 0.01 kJ mol^{-1} (highlighted). Most of these examples are for the larger **Tri^{8}Di^{12}** topology, suggesting that this sequence is less reliable as more degrees of freedom are present. There are few examples where cage stability (determined by a threshold of $E_{\mathrm{b}} = 0.3$ kJ mol^{-1}) changes depending on the run.

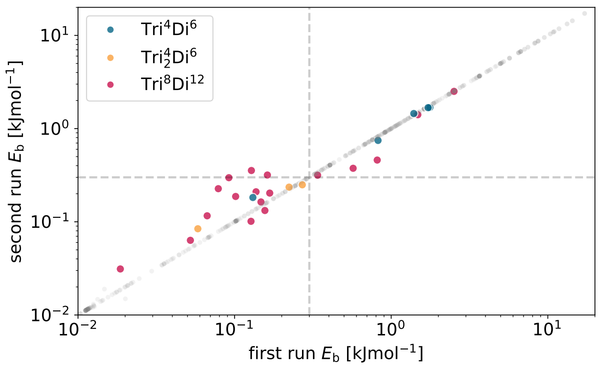

Fig. S2: Parity of E_b values for a selection of cage topologies from two distinct runs (run on the same computer). Only cages with $E_\mathrm{b} > 0.01\,\mathrm{kJ\,mol^{-1}}$ for both runs are shown, and only cages that have a change in $E_\mathrm{b} > 0.01\,\mathrm{kJ\,mol^{-1}}$ between the two runs are shown in colour. Both axes are on a log scale. The horizontal and vertical grey lines show the energy threshold for stability of $E_\mathrm{b} = 0.3\,\mathrm{kJ\,mol^{-1}}$.

S4. Geometry validation and comparing model input to output

In this section, we compare the input and output geometrical properties of our models. The differences between observed and target bond lengths are much smaller than those for angles. Fig. S7 shows that the angles in the tetratopic building blocks tend to be larger than their targets, while the angles in the tritopic building blocks show a mixture of larger and smaller than their targets. Additionally, Fig. S3, Fig. S4 and Fig. S7 show that larger topologies will allow for smaller deviations in the cage angles, where the strain can spread throughout the structure.

Fig. S9 clearly shows the effect of the torsion restriction, where the restriction leads to higher energy structures in most cases (Fig. S10). Although there is still a preference for torsions near 180° when the torsion restriction is off. Fig. S11 shows the observed versus target angles for the bac angle in the ditopic ligands. This angle corresponds to one side of the ditopic ligand, which should be symmetrical if the structure is unstrained; the target bite-angle of the ditopic ligand is $2(\theta_{bac} - 90)$, which is not well-defined if the $baab$ torsion is far from 0°. This data shows that the width of the deviations decreases without the torsion restriction, implying improved structural relaxation.

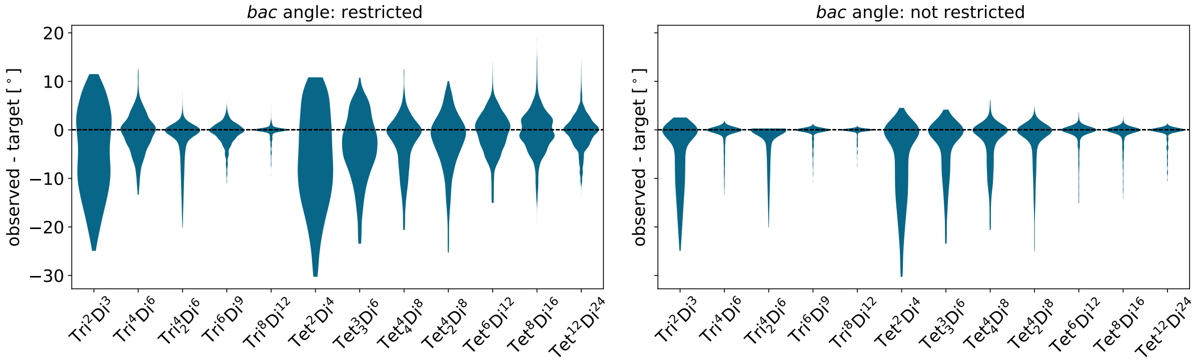

Fig. S3: Distributions of the difference between observed and target bac angles for all topologies, except **Tet⁶Tri⁸**, (left) with and (right) without torsion restrictions.

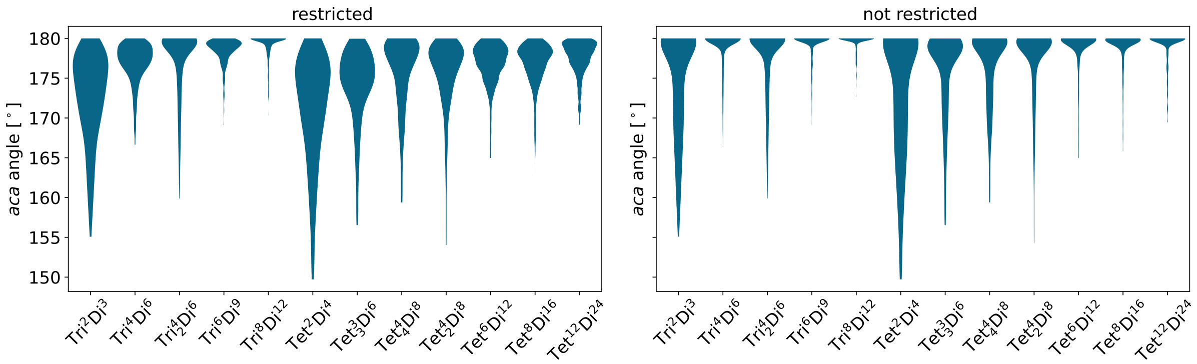

Fig. S4: Distributions of the observed aca angles for all topologies, except **$\mathbf{Tet^6Tri^8}$**, (left) with and (right) without torsion restrictions. The target value is 180°.

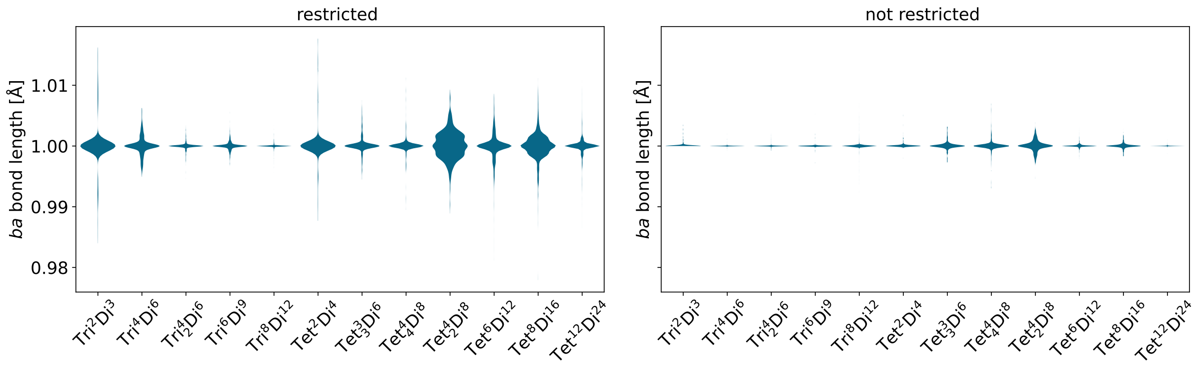

Fig. S5: Distributions of the observed ba bond lengths for all topologies, except **Tet^{6}Tri^8**, (left) with and (right) without torsion restrictions. The target value is $1\,\text{Å}$.

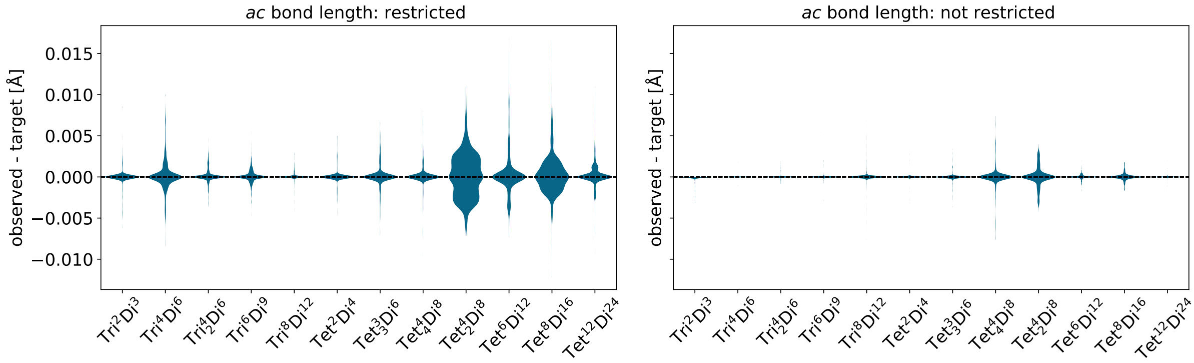

Fig. S6: Distributions of the difference between observed and target ac bond lengths in the ditopic building blocks for all topologies, except **Tet^{6}Tri^8**, (left) with and (right) without torsion restrictions.

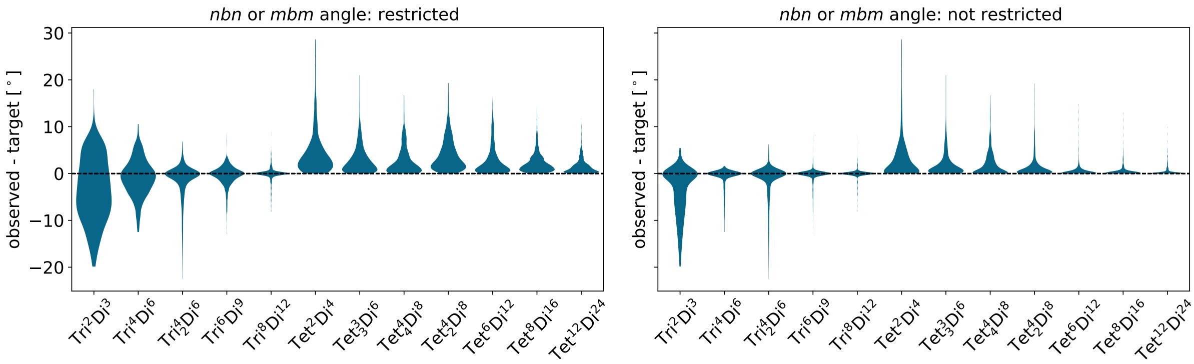

Fig. S7: Distributions of the difference between observed and target nbn (tritopic) or mbm (tetratopic) angles for all topologies, except **Tet^{6}Tri^8**, (left) with and (right) without torsion restrictions.

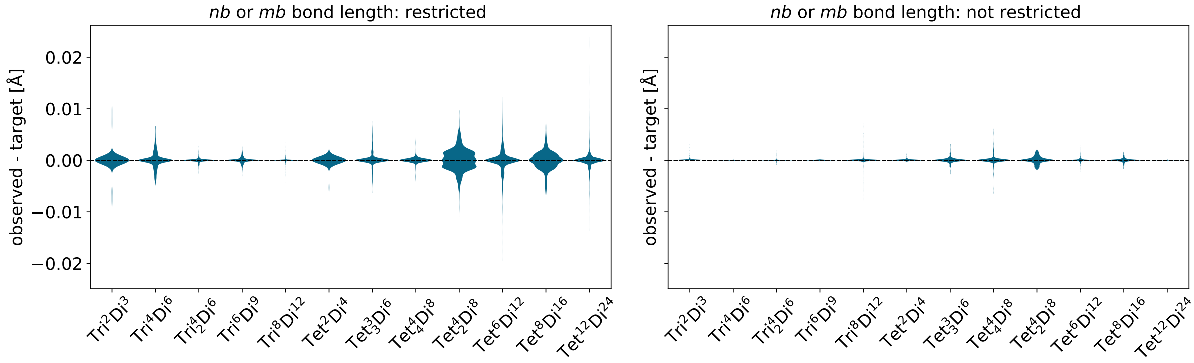

Fig. S8: Distributions of the difference between observed and target nb (tritopic) or mb (tetratopic) bond lengths for all topologies, except **Tet^{6}Tri^8**, (left) with and (right) without torsion restrictions.

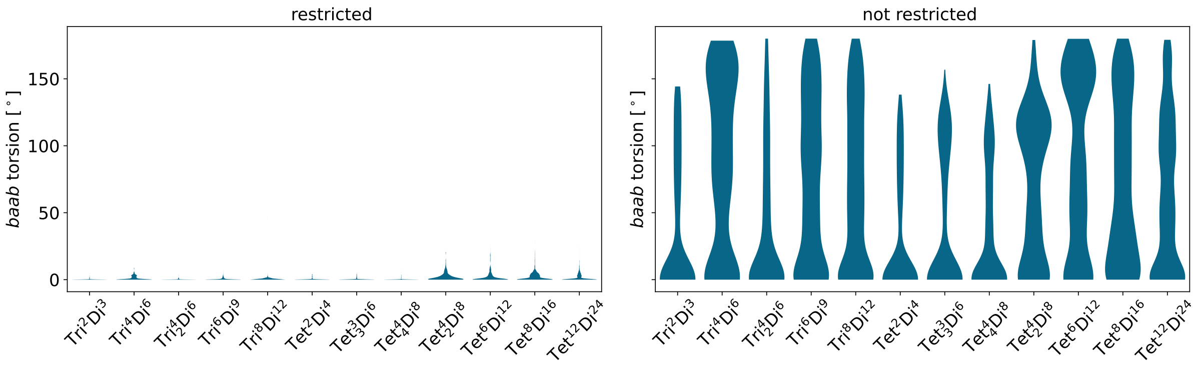

Fig. S9: Distributions of the observed $baab$ torsion angles for all topologies, except **Tet^{6}Tri^8**, (left) with and (right) without torsion restrictions. The target, when applied, is 0°.

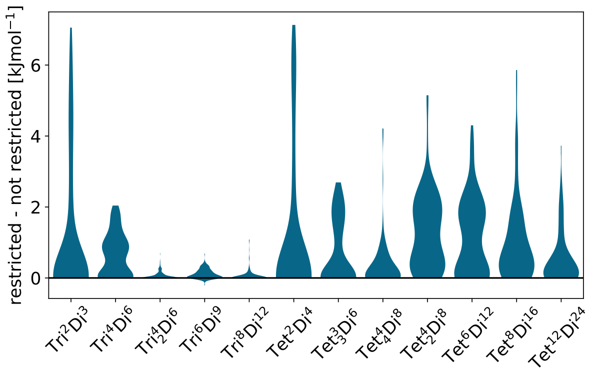

Fig. S10: Distributions of the differences in E_{b} with and without the restricted torsion for all topologies, except **Tet^{6}Tri^8**.

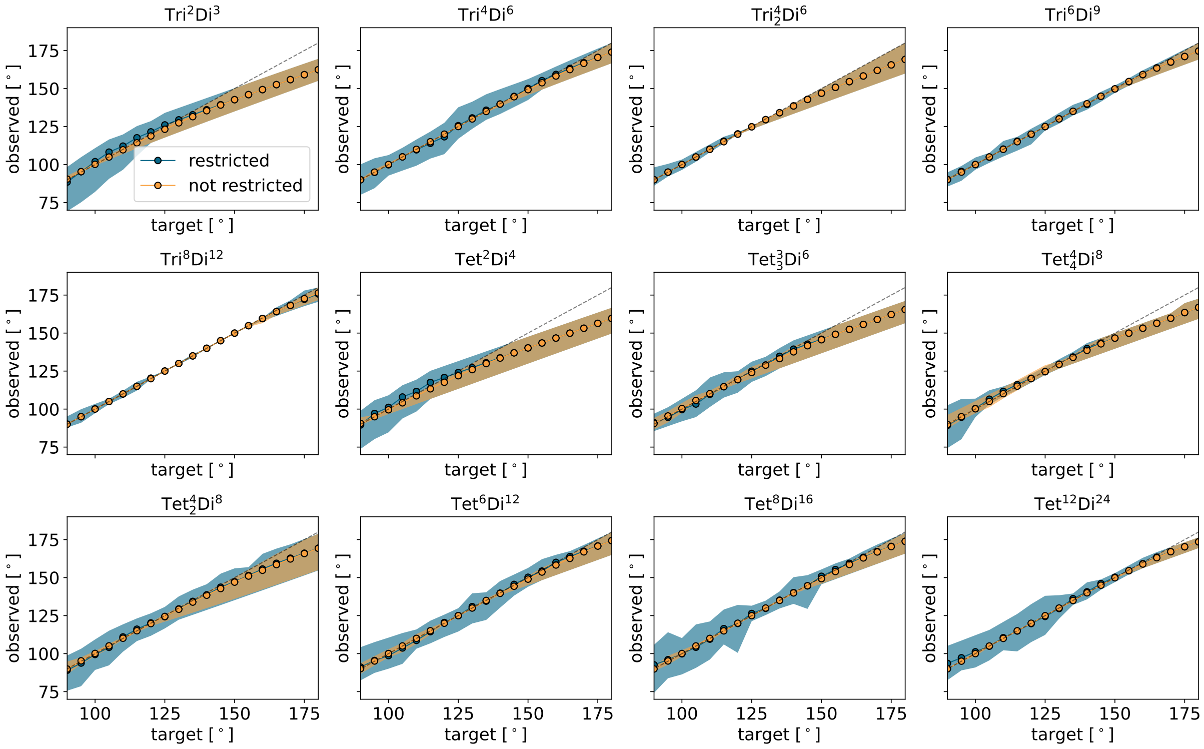

Fig. S11: Parities of mean observed versus target bac angles beads for all topologies, except **Tet^{6}Tri^8**, with and without torsion restrictions. Shaded regions show the maximum and minimum observed angle at each point.

S5. Limitations

In this section, we go through potential limitations to our approach and how those relate to application to real systems. Firstly, we acknowledge that structures in our model may not have any experimental translation, *e.g.*, the inside-out structures, like in Fig. 3(b). Similarly, our model often produces collapsed, interlocked structures, likely one of many unstable conformations.

Ultimately, this model's simplicity results in a likelihood of producing nonsensical structures when there is a lot of strain in the system. This could also result in false positives (regarding stability). We find that there is one case where the torsion-restricted case is lower energy than the unrestricted case, which goes against our expectations because the models can alleviate strain easier without restriction. Looking further into this, Fig. S12 shows the above example and other selected examples from test runs of the generation algorithm (for comparison) where the unrestricted case is higher energy than the restricted case. We propose that the issue is mostly that the geometry optimisation procedure has failed to find the lowest energy minimum in the unrestricted case. This is evident for the $\mathbf{Tet^{12}Di^{24}}$ case, where protrusions are shown (arrow in Fig. S12).

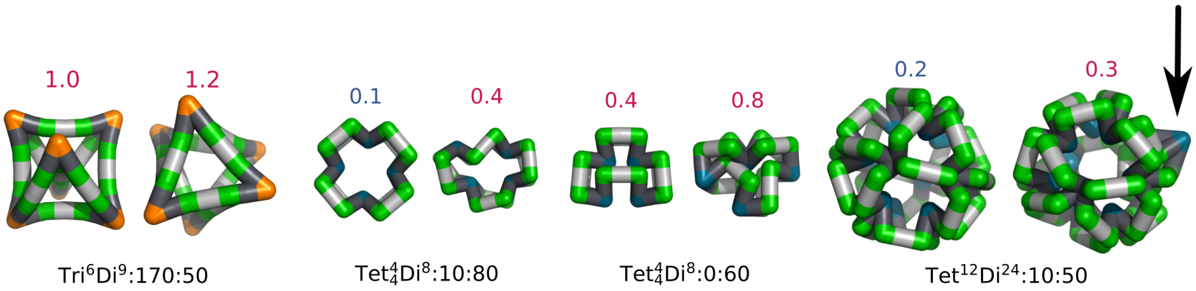

Fig. S12: Pairs of (left) torsion-restricted and (right) unrestricted cage models for cases where the unrestricted case has higher energy. E_b is given above each cage structure, blue if stable, pink if unstable. Topology, target bite angle and pyramid angles are given under each pair. The first pair ($\mathbf{Tri^6Di^9}$:170:50) are from this production run, while the other three are from unshown test runs. The arrow highlights protrusion in a high-energy state. Green are the a beads, orange are the n beads, cyan are the m beads, black are the b beads, and grey are the c beads.

While we have aimed to be as general as possible, we must consider the bias in our model that stems from what is present in the literature. For example, the input, output and design of *stk* depends on what exists in the literature. Hence, the initial structures of topology graphs will match the geometries of building blocks similar to what has been studied before, starting lower in energy than those that deviate from the known cases. This could be overcome by using random coordinates or a distance-geometry method when applying *stk* topology graphs, which we aimed to recreate through the soft-potential MD step. Because of the low-cost nature of minimalistic models, it is possible to generate many, possibly spurious, structures and explore the configuration space around them. These models could also improve the initial guesses within *stk*, by modifying the ideal guesses toward one matching the building block geometries. Ultimately, this model allows us to explore beyond our relatively narrow explored chemical space.

One important limitation in this work is the resolution between changes in bond and angle parameters. This raises the question of whether the resolution we have implemented here (*e.g.*, changing the bac angle in increments of 5°) is sufficient to find a continuous phase space, or if there will be stability cliffs. Further to this, if sharp changes in stability are present in the phase space, are they topological effects that can be taken advantage of in design? These are questions we aim to explore with more focused studies.

S6. Ditopic internal angle relationships.

This section details the ditopic internal angle relationships for each topology. The structures on the right in the following figures were selected to highlight interesting relationships. The circle markers in the left-hand figure map to the structures on the right. In summary, we see:

- Without torsions, stable structures are found with a wide range of internal angles due to the ability to twist the ditopic ligand and form, for example, helical structures.
- The angles in the tritopic/tetratopic building block can greatly modify the shape of the pore in stable structures.
- Many stable structures without torsion restriction are visually collapsed or have internal facing building blocks.
- Stable structures without torsion restrictions can be visually more dynamic (Fig. S17).
- For some topologies, the effect of torsion restriction on the geometry of the stable structure is less significant (Fig. S22).

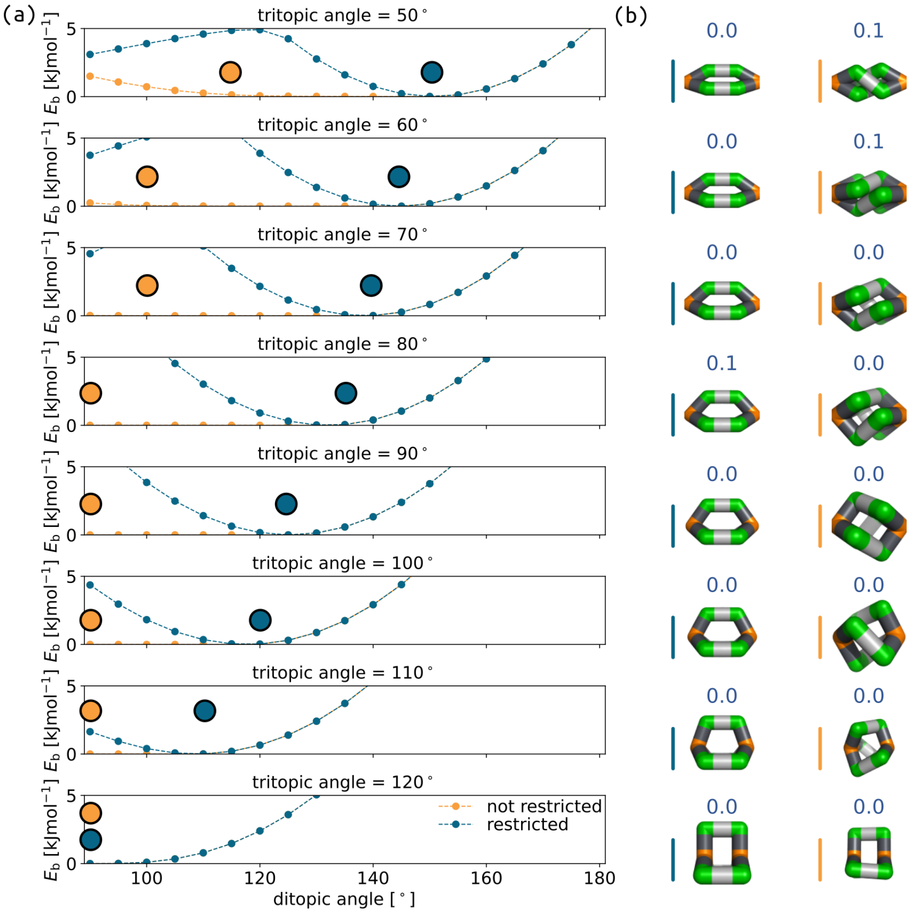

Fig. S13: Internal angle relationship for **Tri[2]Di[3]** topology. (a) Lowest energy configuration E_b versus the target ditopic angle for each pyramid angle (increasing from top-to-bottom), with torsion restrictions (blue) and no restrictions (yellow). (b) Selected structures for each row are shown, matching the points with coloured circles above them in (a). Green are the a beads, orange are the n beads, cyan are the m beads, black are the b beads, and grey are the c beads.

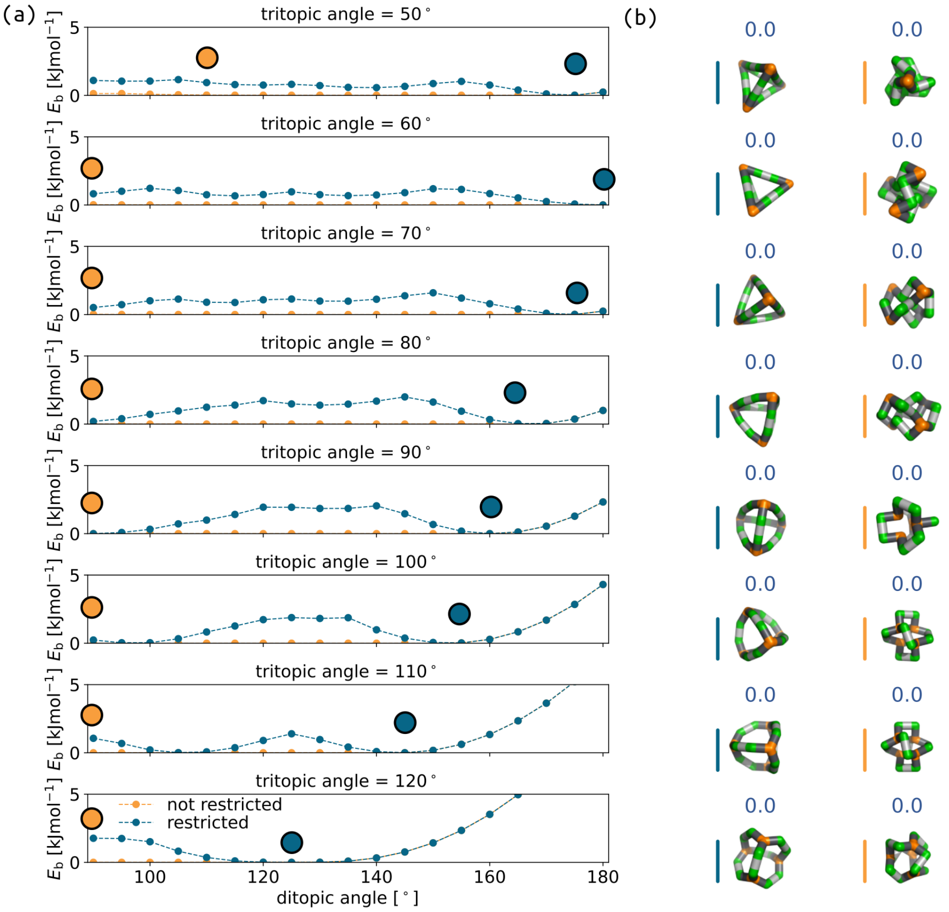

Fig. S14: Internal angle relationship for **Tri^{4}Di^6** topology. (a) Lowest energy configuration E_b versus the target ditopic angle for each pyramid angle (increasing from top-to-bottom), with torsion restrictions (blue) and no restrictions (yellow). (b) Selected structures for each row are shown, matching the points with coloured circles above them in (a). Green are the a beads, orange are the n beads, cyan are the m beads, black are the b beads, and grey are the c beads.

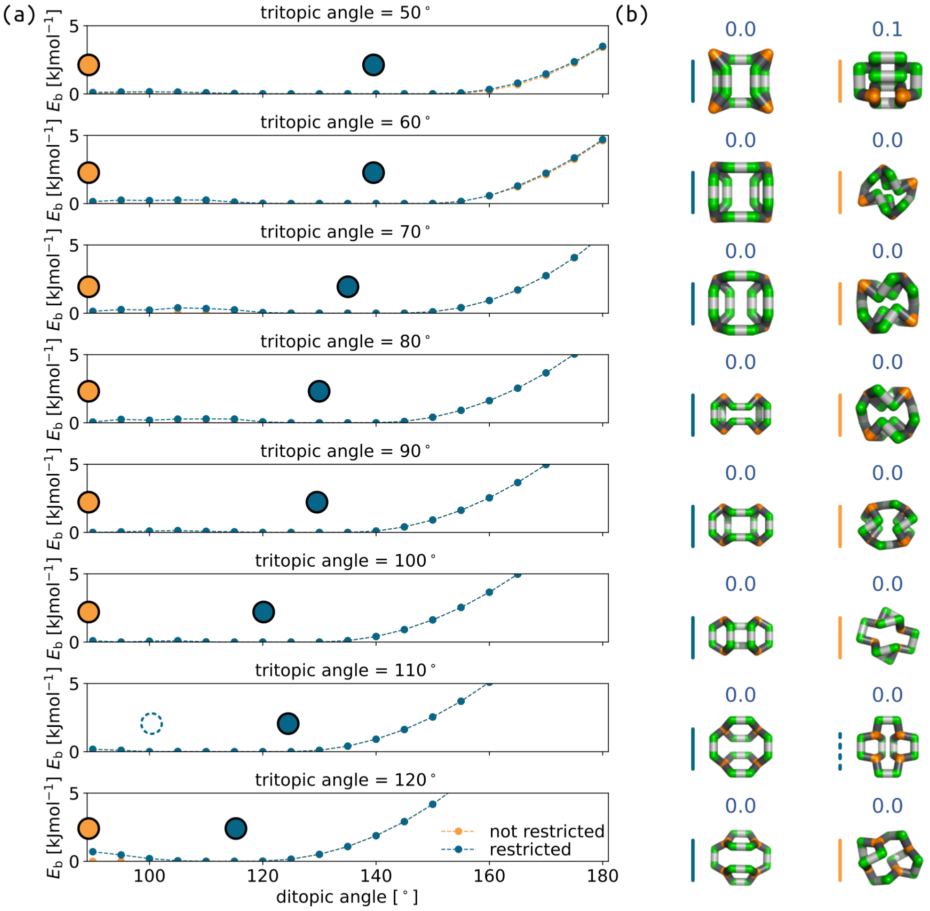

Fig. S15: Internal angle relationship for **Tri$_2^4$Di^6** topology. (a) Lowest energy configuration E_b versus the target ditopic angle for each pyramid angle (increasing from top-to-bottom), with torsion restrictions (blue) and no restrictions (yellow). (b) Selected structures for each row are shown, matching the points with coloured circles above them in (a). Green are the a beads, orange are the n beads, cyan are the m beads, black are the b beads, and grey are the c beads.

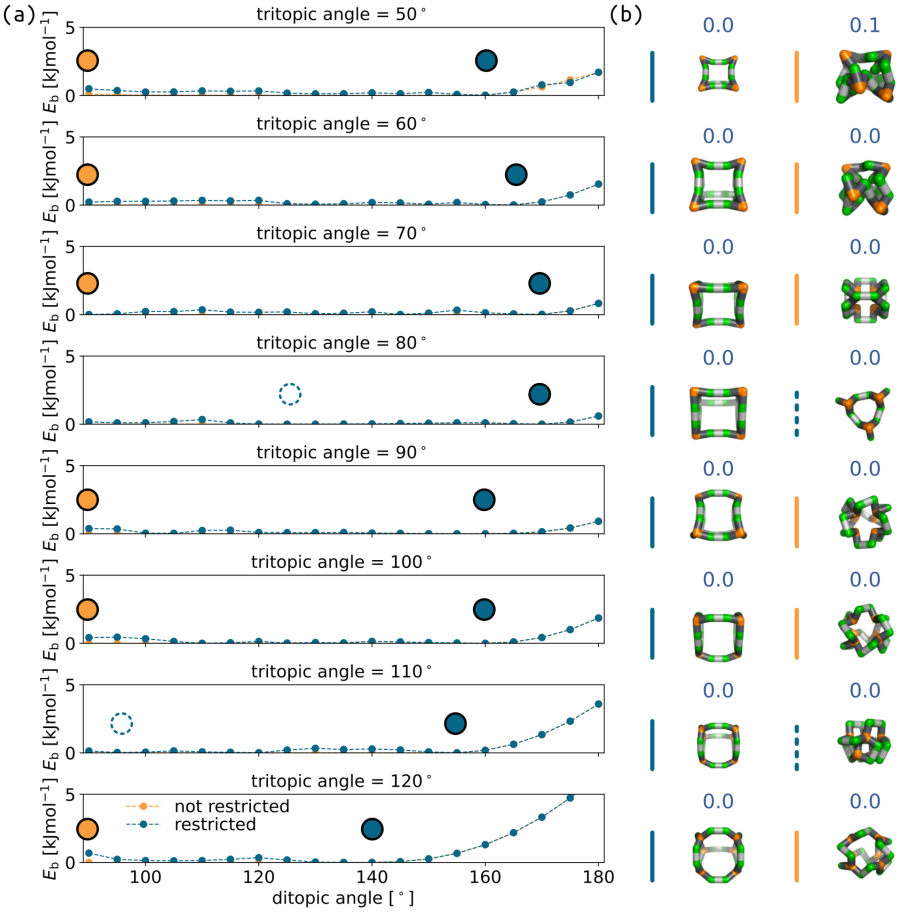

Fig. S16: Internal angle relationship for **Tri^{6}Di^9** topology. (a) Lowest energy configuration E_b versus the target ditopic angle for each pyramid angle (increasing from top-to-bottom), with torsion restrictions (blue) and no restrictions (yellow). (b) Selected structures for each row are shown, matching the points with coloured circles above them in (a). Green are the a beads, orange are the n beads, cyan are the m beads, black are the b beads, and grey are the c beads.

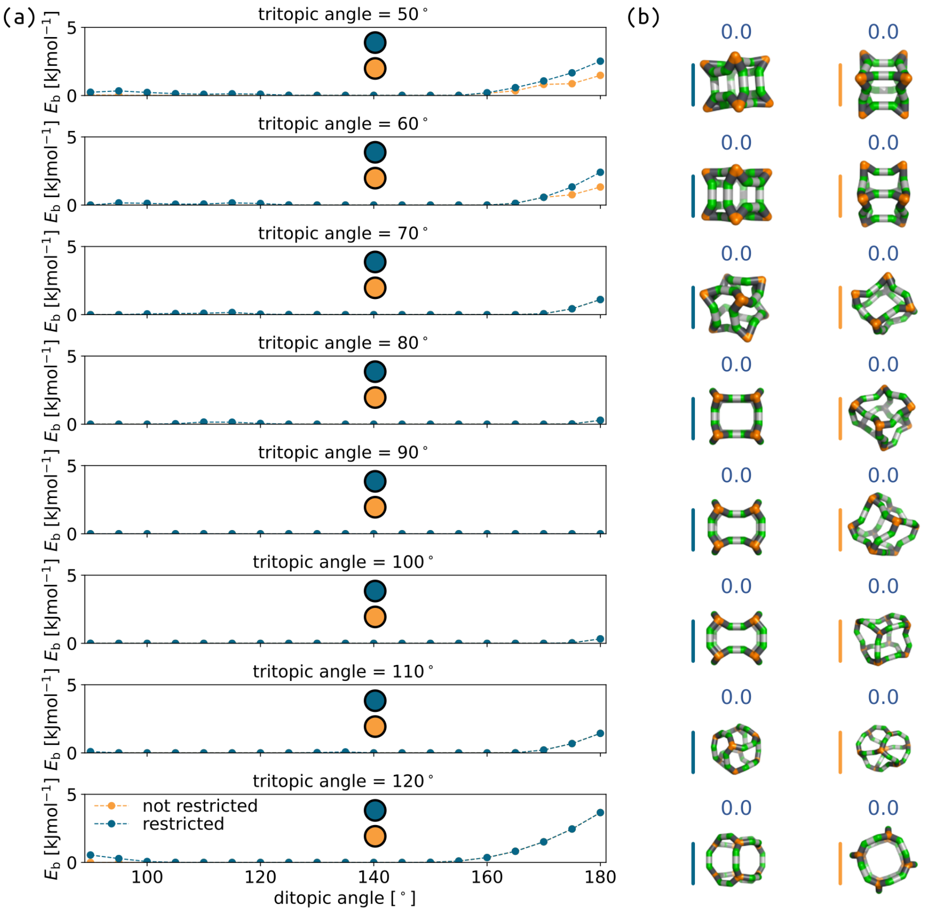

Fig. S17: Internal angle relationship for **Tri^{8}Di^{12}** topology. (a) Lowest energy configuration E_b versus the target ditopic angle for each pyramid angle (increasing from top-to-bottom), with torsion restrictions (blue) and no restrictions (yellow). (b) Selected structures for each row are shown, matching the points with coloured circles above them in (a). Green are the a beads, orange are the n beads, cyan are the m beads, black are the b beads, and grey are the c beads.

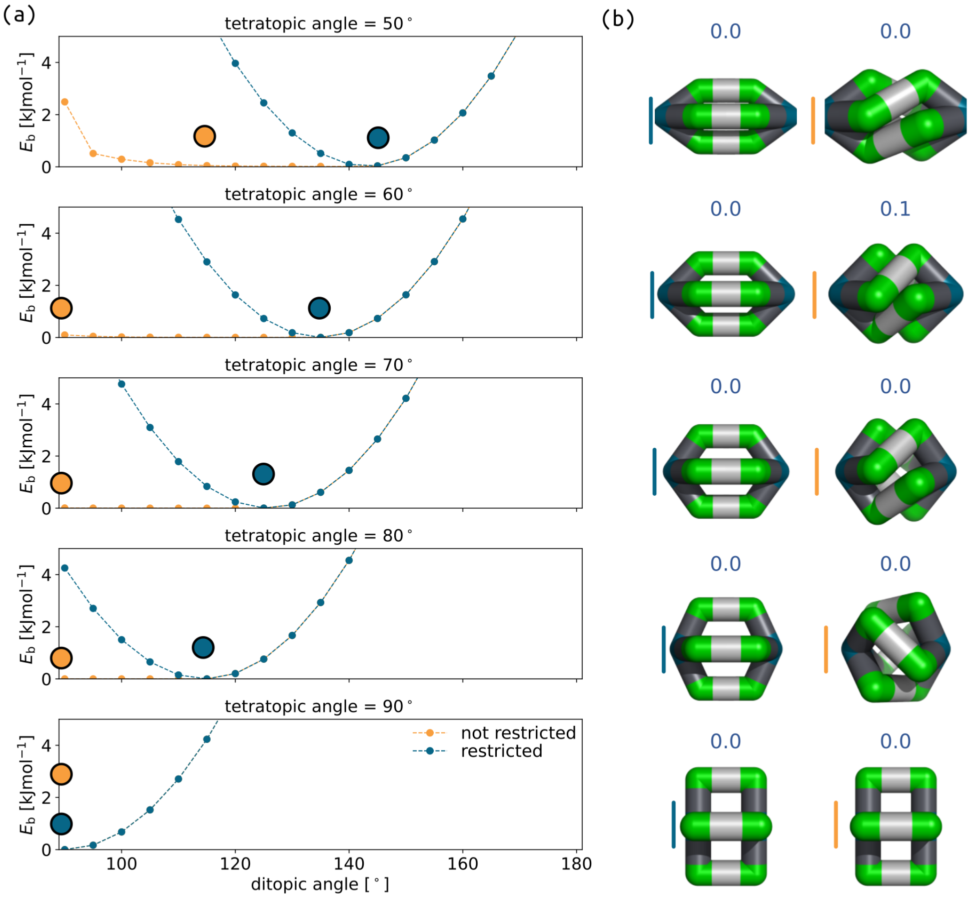

Fig. S18: Internal angle relationship for **Tet^{2}Di^4** topology. (a) Lowest energy configuration E_b versus the target ditopic angle for each pyramid angle (increasing from top-to-bottom), with torsion restrictions (blue) and no restrictions (yellow). (b) Selected structures for each row are shown, matching the points with coloured circles above them in (a). Green are the a beads, orange are the n beads, cyan are the m beads, black are the b beads, and grey are the c beads.

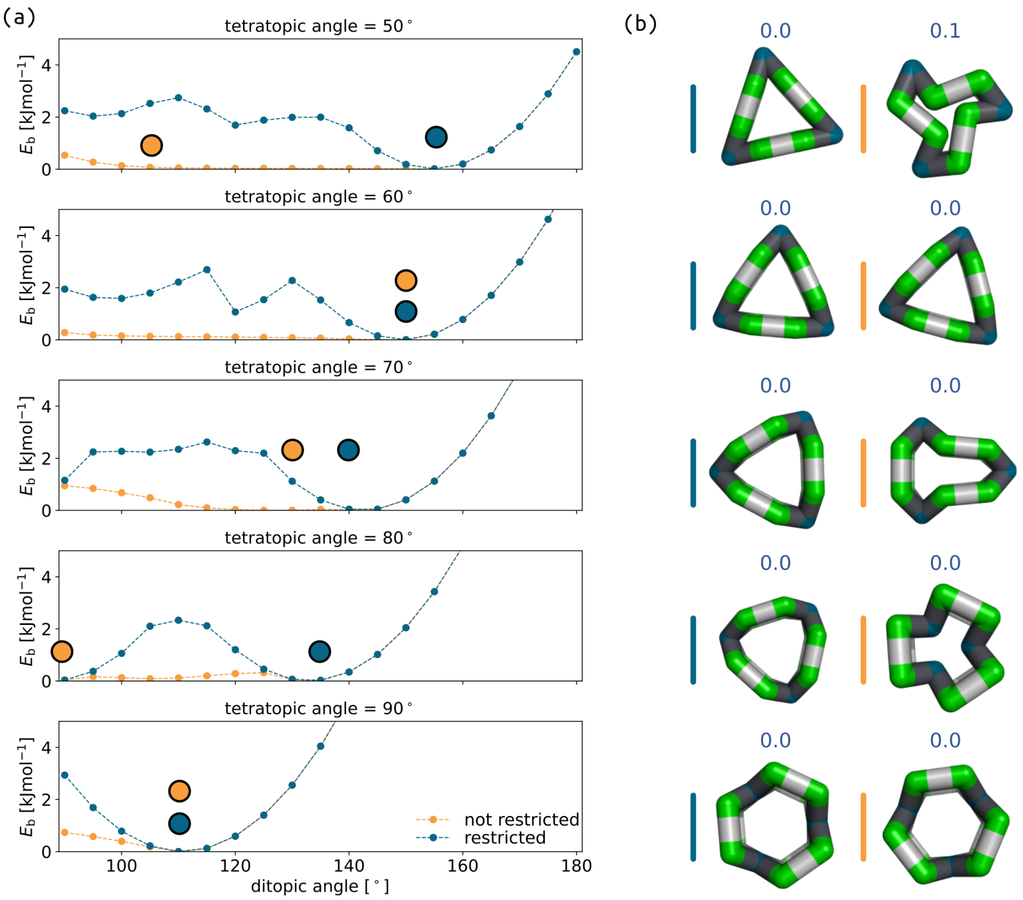

Fig. S19: Internal angle relationship for $\mathbf{Tet}_3^3\mathbf{Di}^6$ topology. (a) Lowest energy configuration E_b versus the target ditopic angle for each pyramid angle (increasing from top-to-bottom), with torsion restrictions (blue) and no restrictions (yellow). (b) Selected structures for each row are shown, matching the points with coloured circles above them in (a). Green are the a beads, orange are the n beads, cyan are the m beads, black are the b beads, and grey are the c beads.

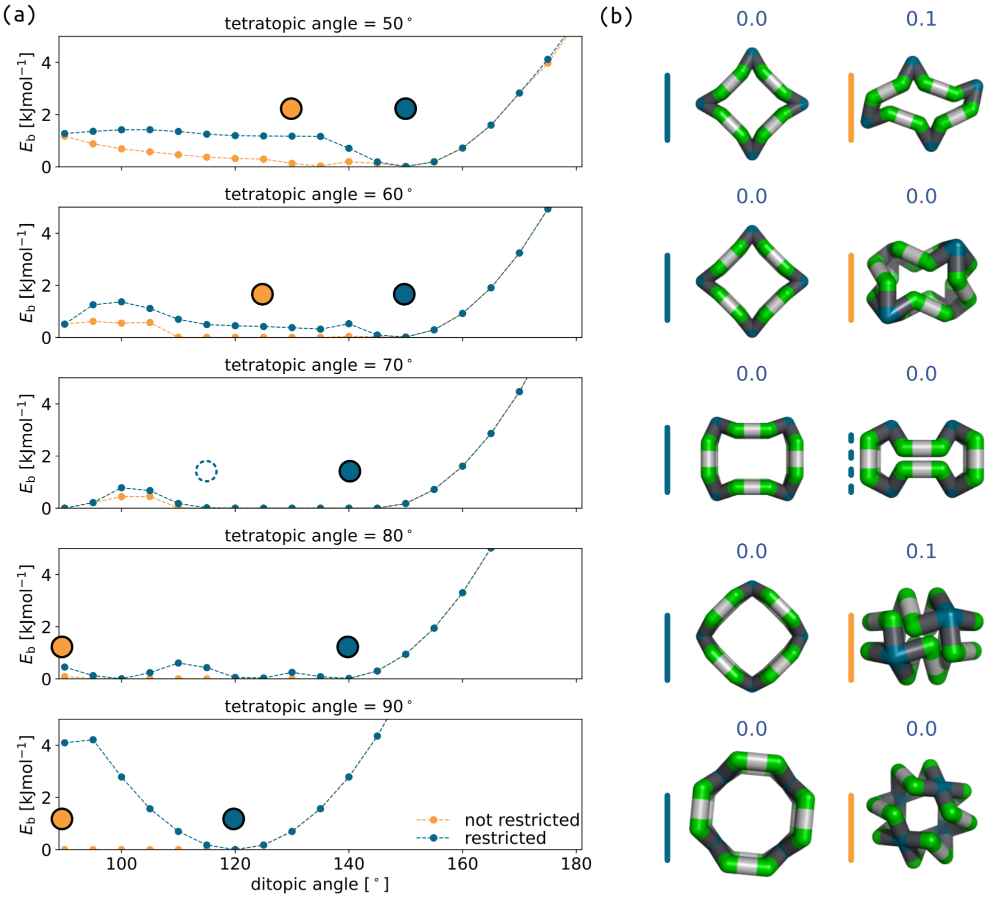

Fig. S20: Internal angle relationship for $\mathbf{Tet_4^4Di^8}$ topology. (a) Lowest energy configuration E_{b} versus the target ditopic angle for each pyramid angle (increasing from top-to-bottom), with torsion restrictions (blue) and no restrictions (yellow). (b) Selected structures for each row are shown, matching the points with coloured circles above them in (a). Green are the a beads, orange are the n beads, cyan are the m beads, black are the b beads, and grey are the c beads.

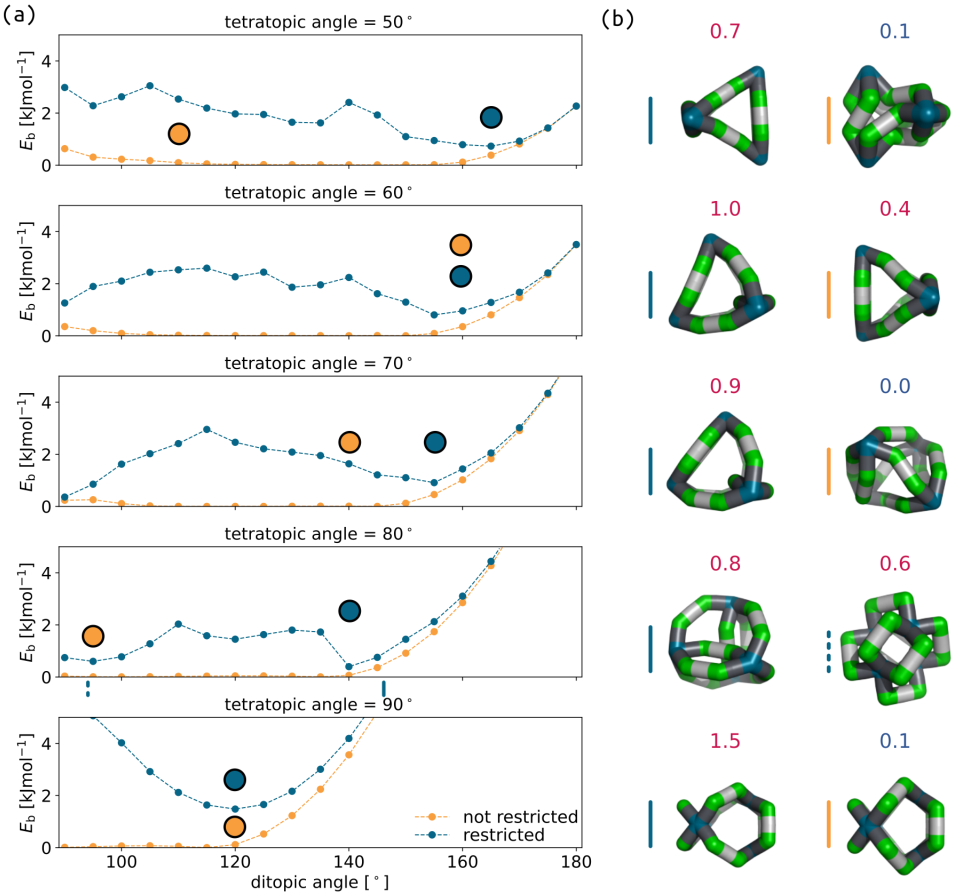

Fig. S21: Internal angle relationship for $\mathbf{Tet^4_2Di^8}$ topology. (a) Lowest energy configuration E_b versus the target ditopic angle for each pyramid angle (increasing from top-to-bottom), with torsion restrictions (blue) and no restrictions (yellow). (b) Selected structures for each row are shown, matching the points with coloured circles above them in (a). Green are the a beads, orange are the n beads, cyan are the m beads, black are the b beads, and grey are the c beads.

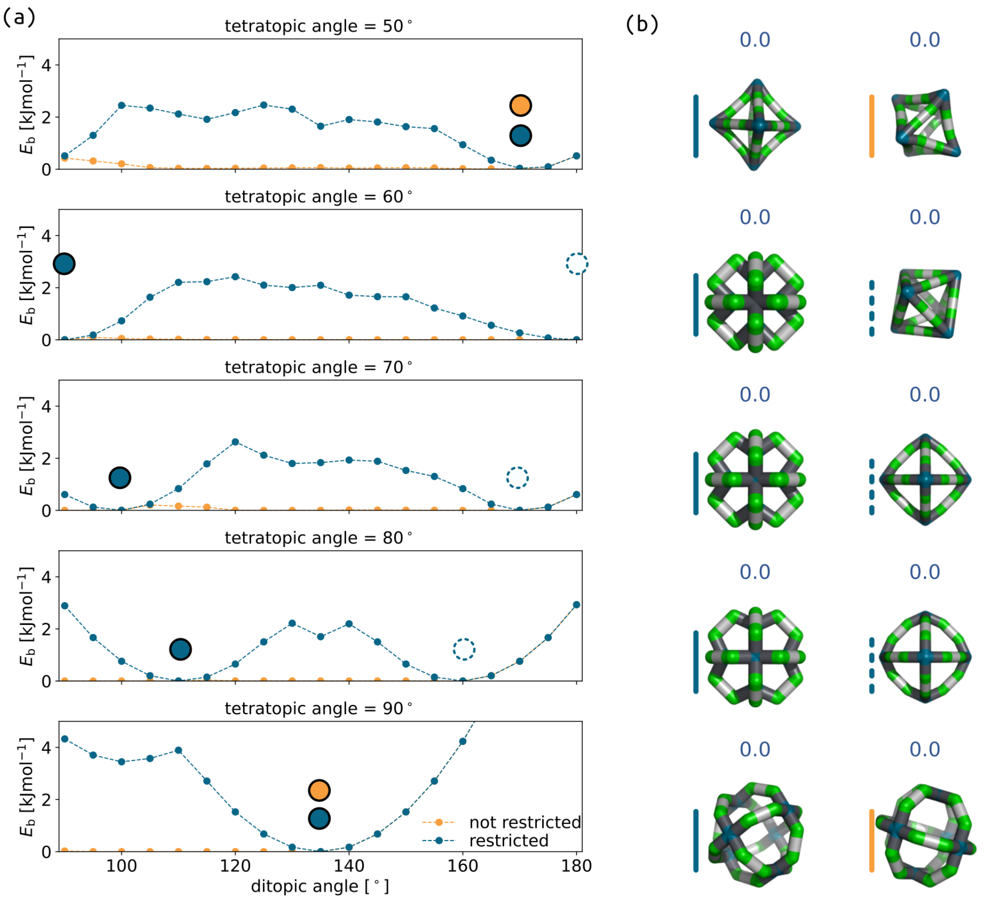

Fig. S22: Internal angle relationship for **Tet^{6}Di^{12}** topology. (a) Lowest energy configuration E_b versus the target ditopic angle for each pyramid angle (increasing from top-to-bottom), with torsion restrictions (blue) and no restrictions (yellow). (b) Selected structures for each row are shown, matching the points with coloured circles above them in (a). Green are the a beads, orange are the n beads, cyan are the m beads, black are the b beads, and grey are the c beads.

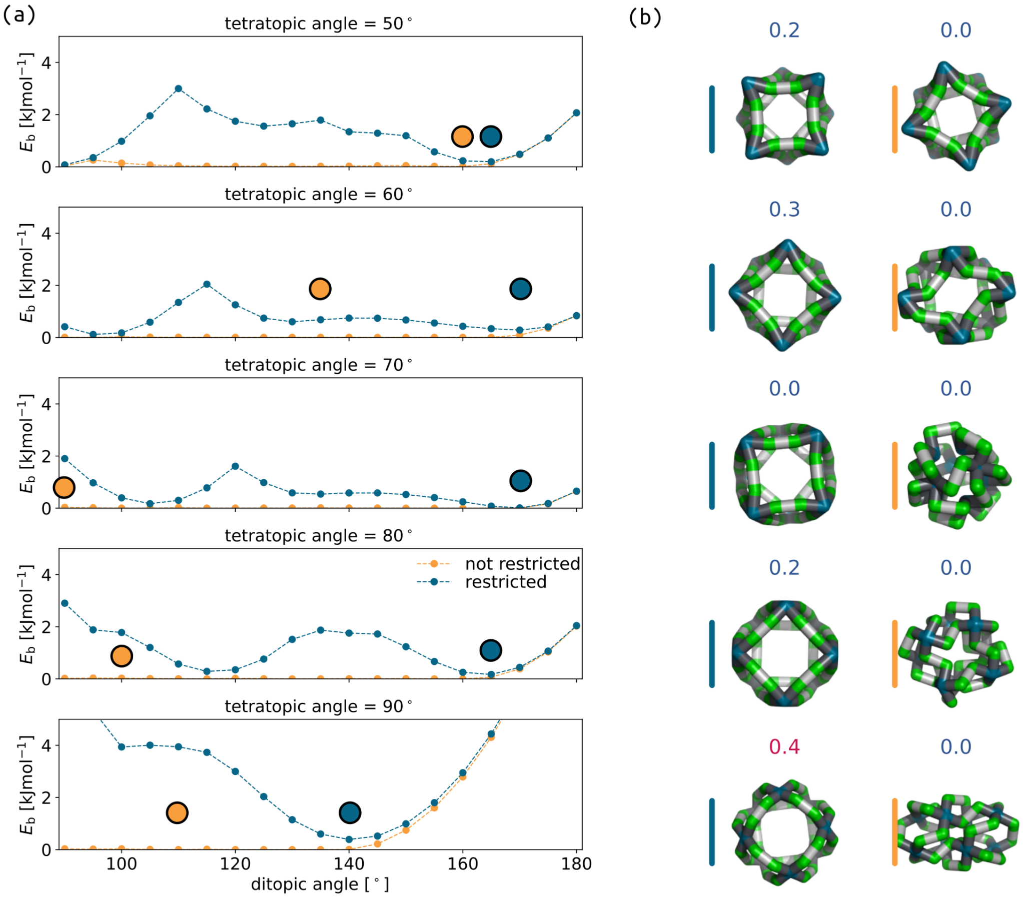

Fig. S23: Internal angle relationship for **Tet^{8}Di^{16}** topology. (a) Lowest energy configuration E_b versus the target ditopic angle for each pyramid angle (increasing from top-to-bottom), with torsion restrictions (blue) and no restrictions (yellow). (b) Selected structures for each row are shown, matching the points with coloured circles above them in (a). Green are the a beads, orange are the n beads, cyan are the m beads, black are the b beads, and grey are the c beads.

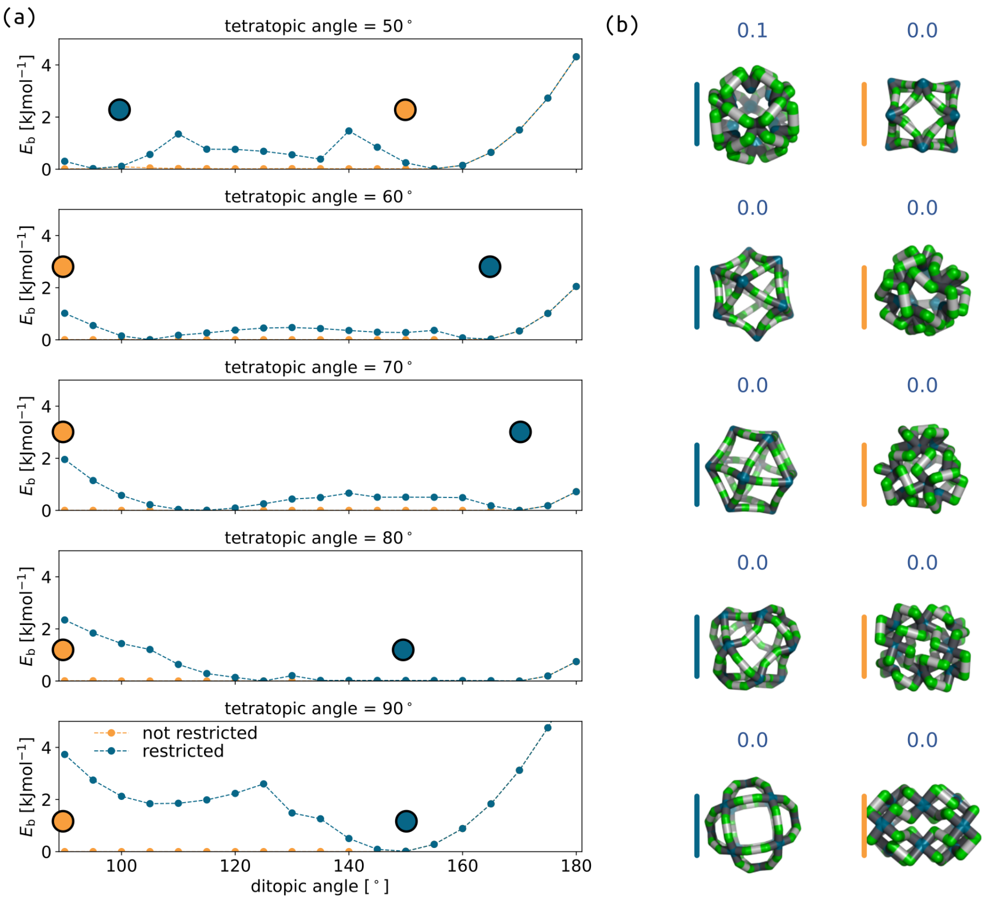

Fig. S24: Internal angle relationship for **Tet^{12}Di^{24}** topology. (a) Lowest energy configuration E_b versus the target ditopic angle for each pyramid angle (increasing from top-to-bottom), with torsion restrictions (blue) and no restrictions (yellow). (b) Selected structures for each row are shown, matching the points with coloured circles above them in (a). Green are the a beads, orange are the n beads, cyan are the m beads, black are the b beads, and grey are the c beads.

S7. Angle maps

The following series of plots show a map of stability over the target angles for each topology, with and without torsion restrictions. While some topologies show different qualitative relationships at low to middle target ditopic internal angles, all topologies have a "drop-off" in stability at high ditopic internal angles, which depends on the pyramid angle and topology. For example, the top right-hand corner (high ditopic internal angle, near-planar pyramid angle) of all topologies is unstable. However, the bottom right-hand corner (high ditopic internal angle, far-from-planar pyramid angle) is stable for some topologies. Fig. S33 shows the interesting lack of stability for the **Tet$_2^4$Di^8** topology and that if the energy scale is increased, there is some structure to the torsion restricted cases. This data is summarised in Fig. S37.

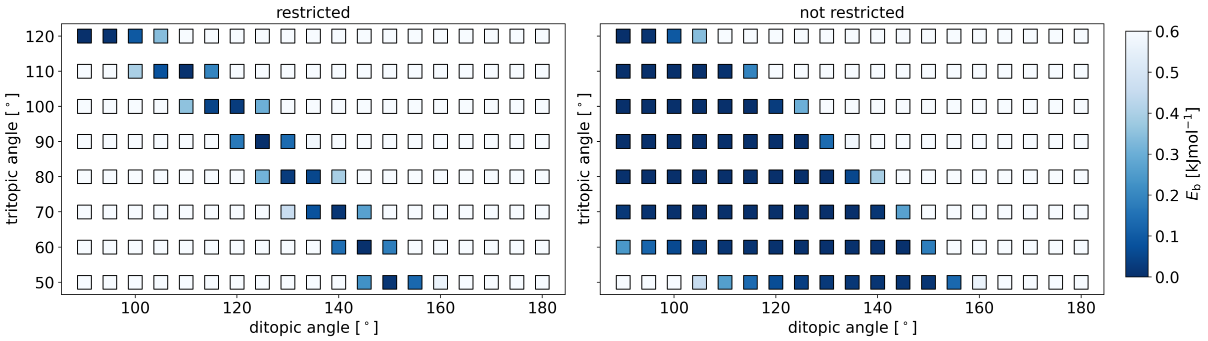

Fig. S25: Angle map for the **Tri^{2}Di^3** topology with and without torsion restriction.

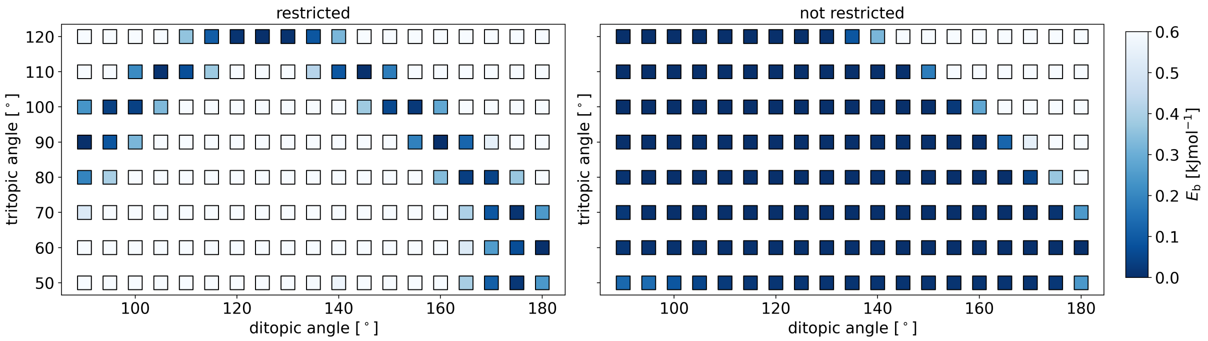

Fig. S26: Angle map for the **Tri^{4}Di^6** topology with and without torsion restriction.

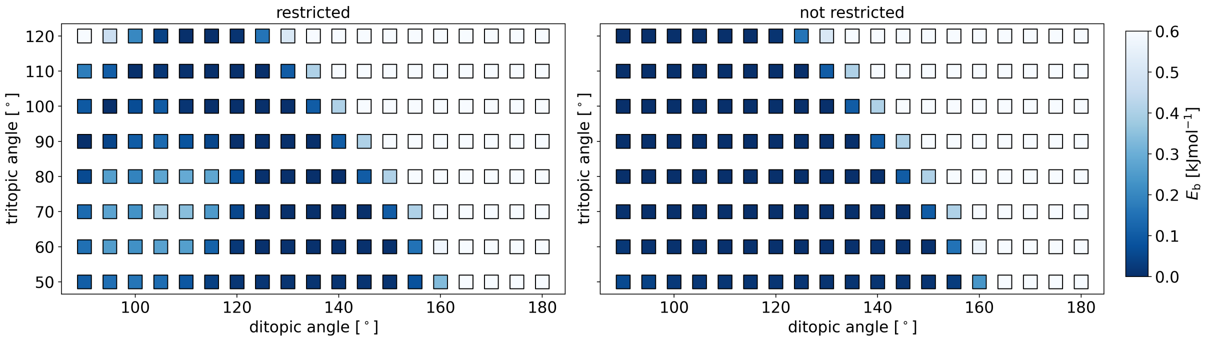

Fig. S27: Angle map for the **Tri$^4_2$Di^6** topology with and without torsion restriction.

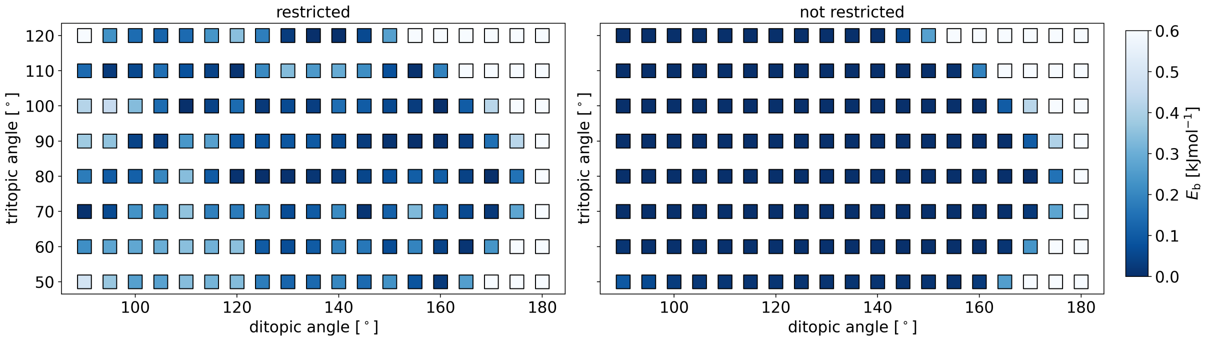

Fig. S28: Angle map for the **Tri^{6}Di^9** topology with and without torsion restriction.

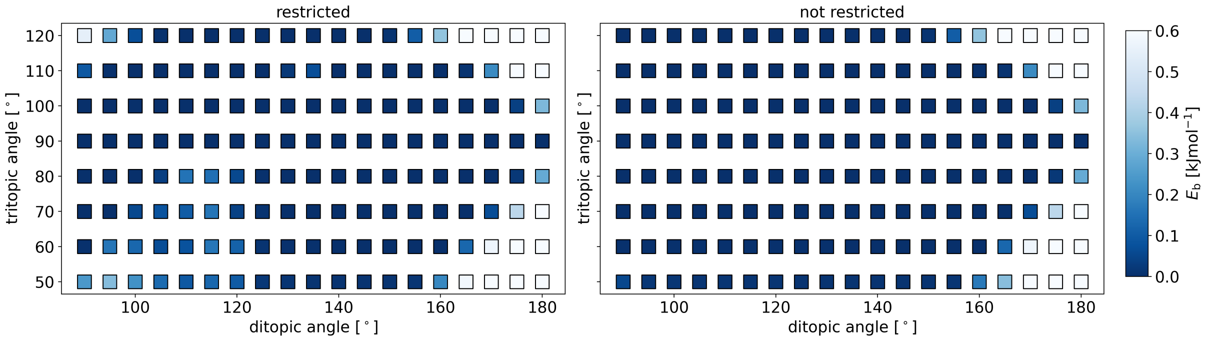

Fig. S29: Angle map for the **Tri^{8}Di^{12}** topology with and without torsion restriction.

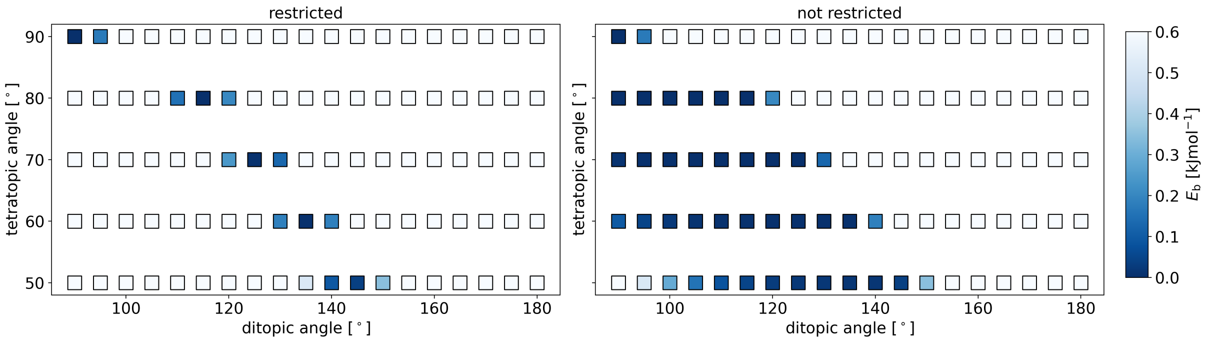

Fig. S30: Angle map for the **Tet^{2}Di^4** topology with and without torsion restriction.

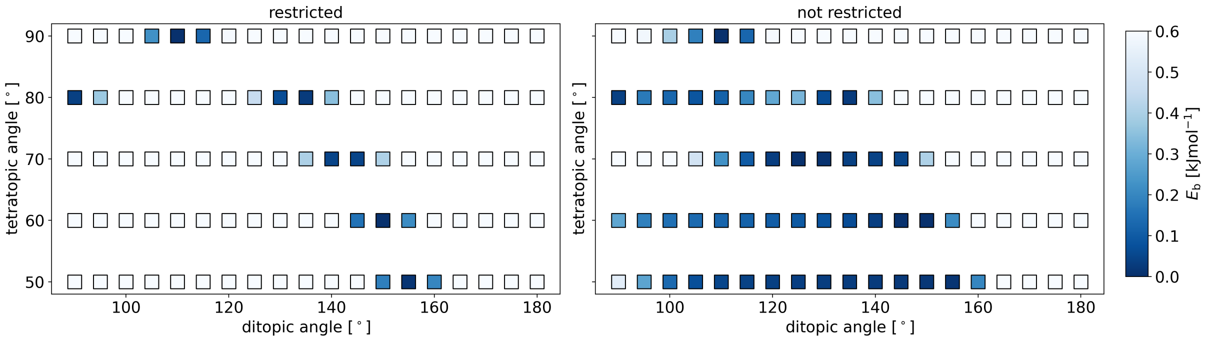

Fig. S31: Angle map for the **Tet$^3_3$Di^6** topology with and without torsion restriction.

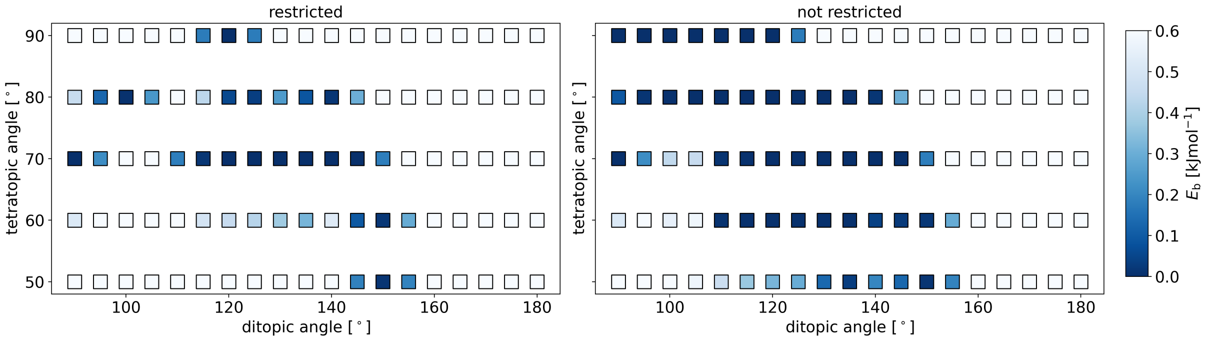

Fig. S32: Angle map for the **Tet$^4_4$Di^8** topology with and without torsion restriction.

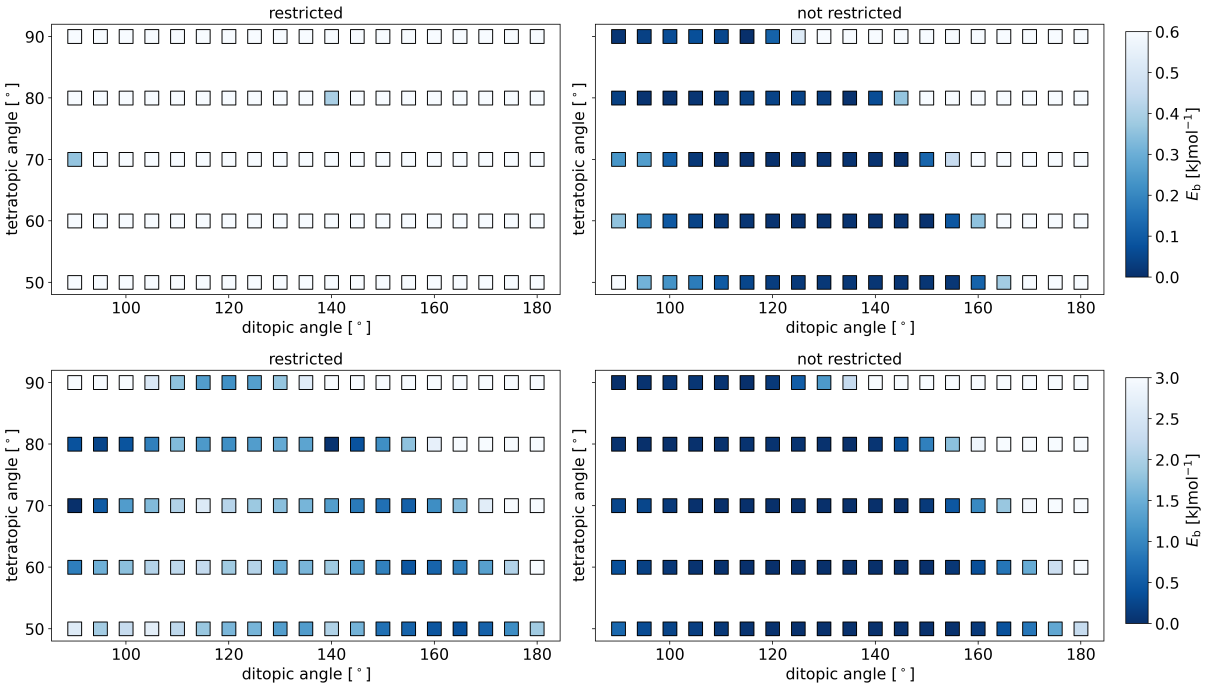

Fig. S33: Angle map for the **Tet$^4_2$Di^8** topology with and without torsion restriction (top) using the same colour scale as other angle maps and (bottom) with an increased scale to visualise higher energy structures.

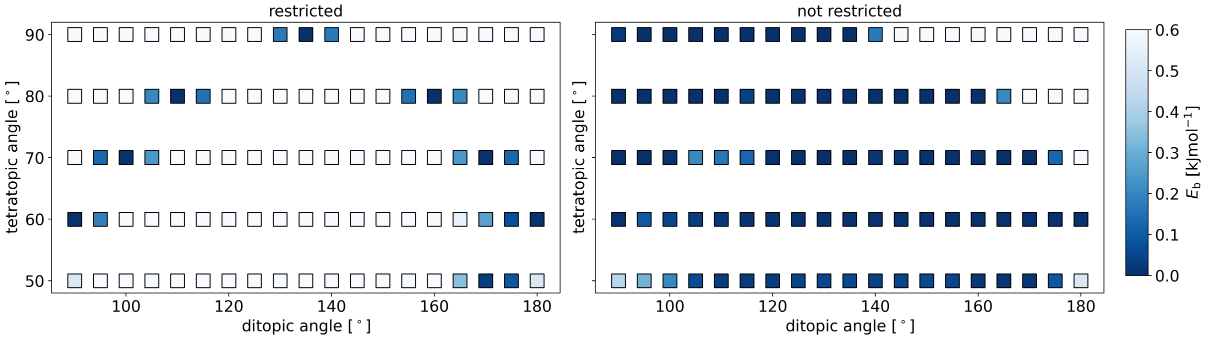

Fig. S34: Angle map for the **Tet^{6}Di^{12}** topology with and without torsion restriction.

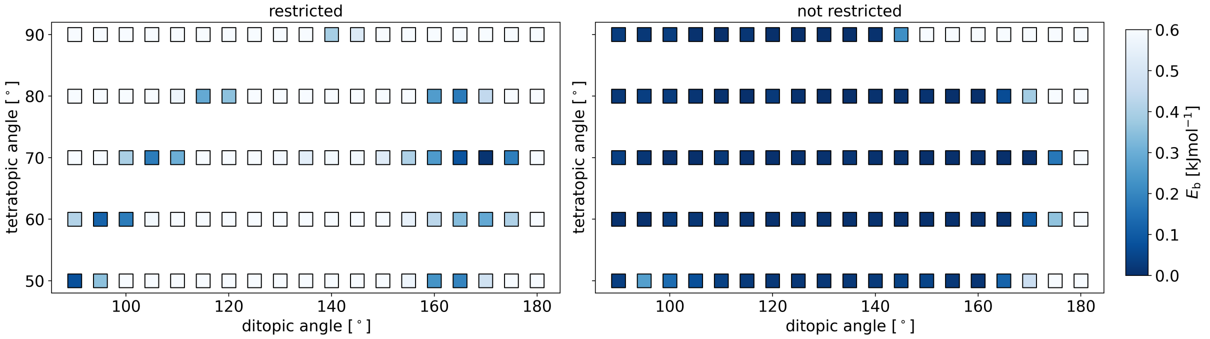

Fig. S35: Angle map for the **Tet^{8}Di^{16}** topology with and without torsion restriction.

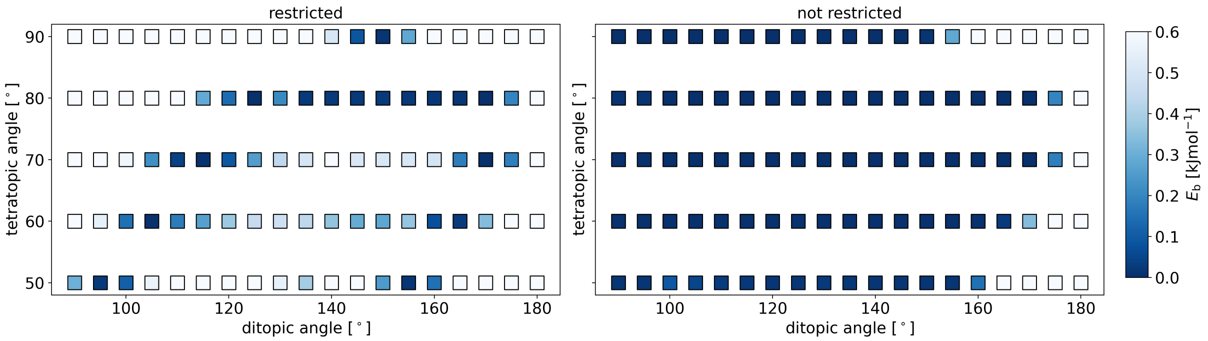

Fig. S36: Angle map for the **Tet^{12}Di^{24}** topology with and without torsion restriction.

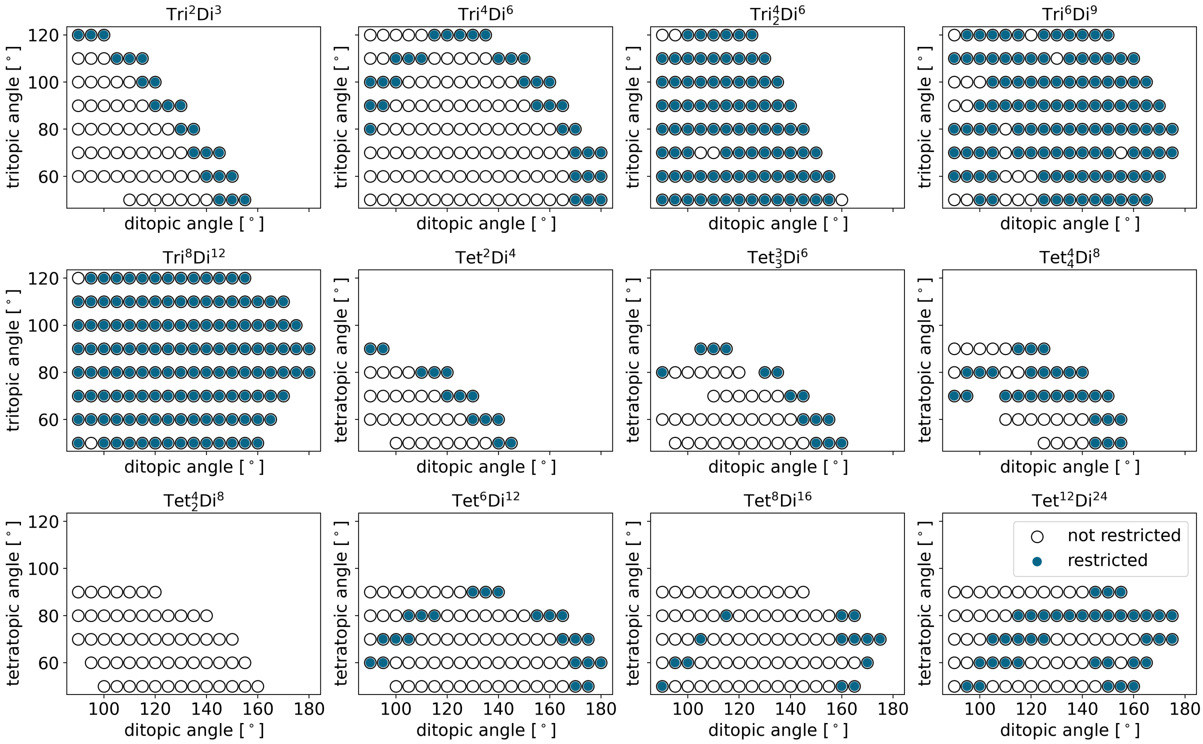

Fig. S37: Summary of the effect of flexibility in angle maps studied in this work for each topology, except **Tet^{6}Tri^8**. Stable structures have $E_\mathrm{b} < 0.3\,\mathrm{kJ\,mol^{-1}}$. Blue points are stable structures with torsion restrictions, while white points with a black border are stable structures without torsion restrictions.

S8. Tet^{6}Tri^8 system

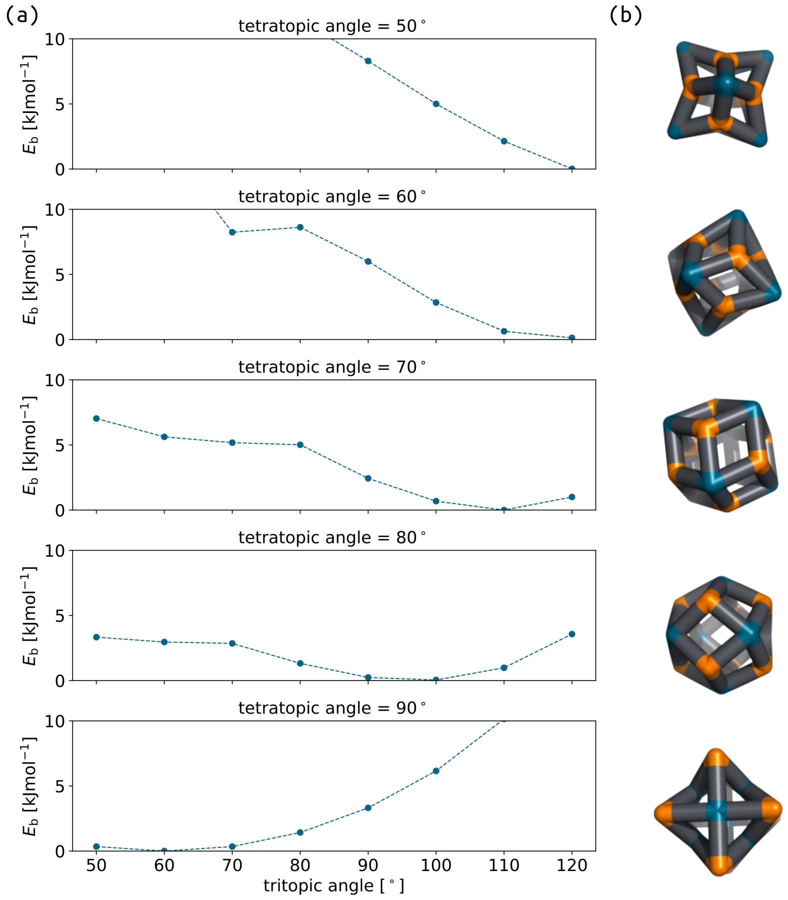

Fig. S38: (a) Energy of structures over tritopic angle range for each tetratopic angle. (b) Low energy structure examples for each row. Orange are the n beads, cyan are the m beads, and black are the b beads.

S9. Distributions of cage energetics

Here we collate information about the energy distributions in our cage data set. Firstly, Fig. S39 shows the structures of the lowest (or one of the lowest) and highest E_b structures for each topology. This figure highlights that there is not a dramatic change in structure for most topologies, but simply the presence of extreme strain between beads. This is further supported by the dominant nature of the angular terms in the component energy distributions (Fig. S41). Fig. S40 shows the distribution of energies for each topology, and shows that strain can spread out in larger structures, where the distribution of energies tends to be lower.

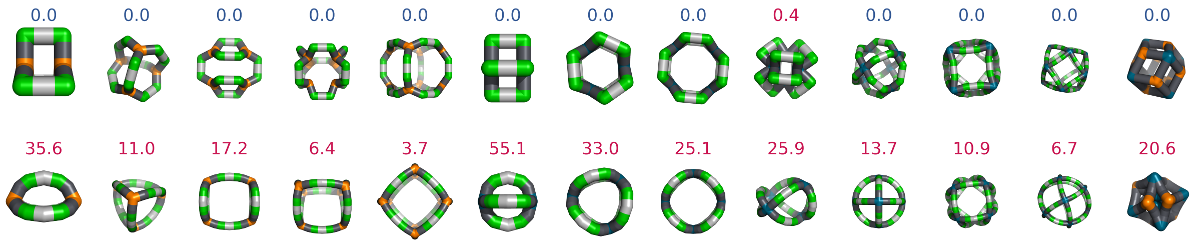

Fig. S39: Structures of the (top) minimum and (bottom) maximum E_b cage for each topology (with torsion restrictions on, if applicable). E_b is shown above each structure in kJ mol^{-1}. Green are the a beads, orange are the n beads, cyan are the m beads, black are the b beads, and grey are the c beads.

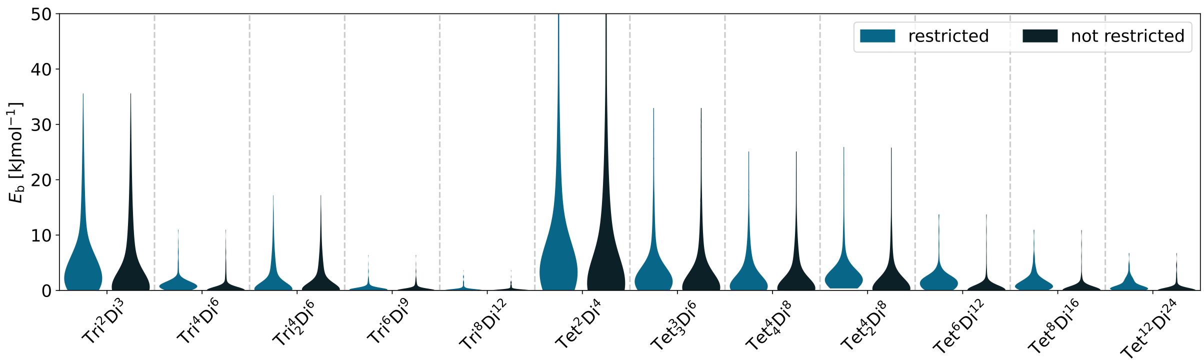

Fig. S40: Distribution of E_b for all topologies (excluding **Tet^{6}Tri^8**), with and without torsion restriction.

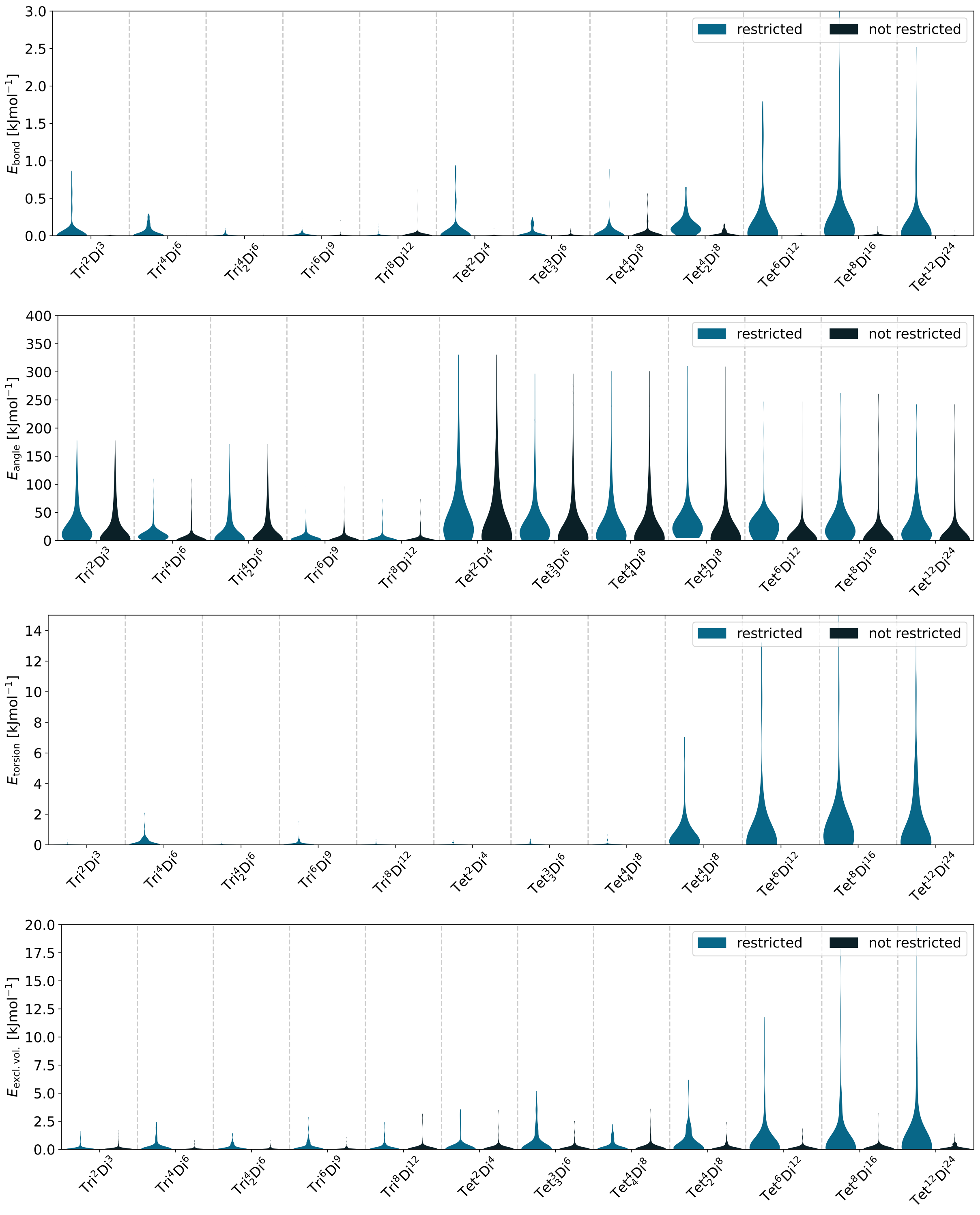

Fig. S41: From top-to-bottom, distribution of energy components of bond, angle, torsion and nonbonded terms for all topologies (excluding **Tet^{6}Tri^8**), with and without torsion restriction. Note the changes in the y-axis scales.

S10. Self-sorting behaviour and accessible topology maps

Self-sorting, as we define in this work, is the thermodynamic selection of a single species over all others that can possibly form during the self-assembly pathway of a set of building blocks. This corresponds to the experimental goal of performing self-assembly and realising the clean formation of a single species after characterisation. Here, we look, very approximately, at the topological effects on self-sorting behaviour. We approximate three outcomes: i) "selected" with only one stable topology, ii) "mixed" with more than one stable topology, and iii) "unstable" with no stable topologies for a given point in the accessible topology maps.

Firstly, Fig. S42 shows that the percentage of selected topologies as a function of threshold energy depends on the tritopic or tetratopic pyramid angle. There seems to be less selectivity with intermediate pyramid angles, than the extremes, which may be an outcome of the types of topologies studied. This can be related to the angle maps (Section S7), where for some topologies, smaller pyramid angles allow for larger ditopic internal angles to fit in the topology before the stability "drop" that

occurs at larger ditopic angles. It is possible that this result leads to increased competition, hence decreased selectivity, for those angle combinations. The balance between achieving stability and self-sorting is not trivial. Hence, predictive tools can be useful. Interestingly, the square planar (90°, tetratopic) systems show much higher percentages of selectivity than the trigonal planar (120°, tritopic) systems. Fig. S43 shows that the tetratopic-containing systems consistently have less "mixed" and more "unstable" isomers than the tritopic-containing isomers.

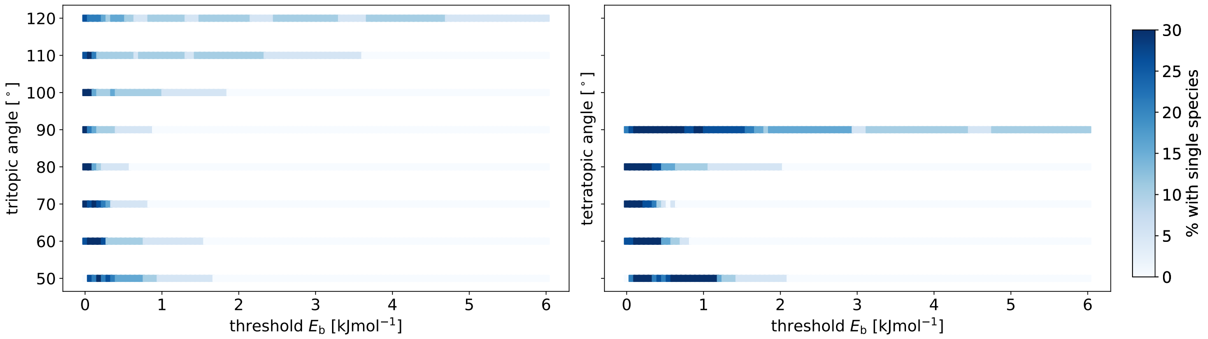

Fig. S42: The percentage of selected topologies for all building block pairs (with torsion restrictions on) separated by the angle in the tritopic or tetratopic building block as a function of the threshold for stability. This analysis is only applied to $\mathbf{Tri_p^mDi^n}$ and $\mathbf{Tet_p^mDi^n}$ topologies.

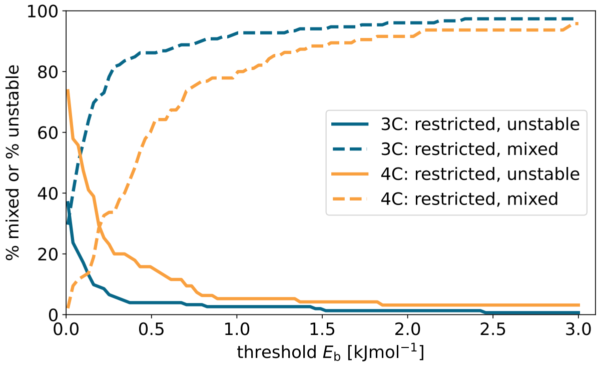

Fig. S43: The percentage of mixed (dashed lines) and unstable (solid lines) topologies for all building block pairs with restrictions as a function of the threshold for stability. This analysis is only applied to $\mathbf{Tri_p^mDi^n}$ and $\mathbf{Tet_p^mDi^n}$ topologies.

Fig. S44 and Fig. S45 show the accessible topology maps with and without torsion restrictions for tritopic and tetratopic containing structures, respectively. We see a general area of instability at large ditopic angles and large pyramid angles (top right-hand corner of plots), matching the results in Section S7 and a shift to larger topologies from left-to-right. However, this shift depends on the pyramid angles and topology/cage flexibility. For example, there are also unstable points in the bottom left-hand corner when torsion restrictions are on, and in the tetratopic-containing maps (because the tritopic-containing maps include a very flexible topology, $\mathbf{Tri_2^4Di^6}$, which is stable in most of this region). We also show the same data but only highlight the smallest stable topology at each point. It is possible that experimental conditions could be chosen to target or avoid this outcome.

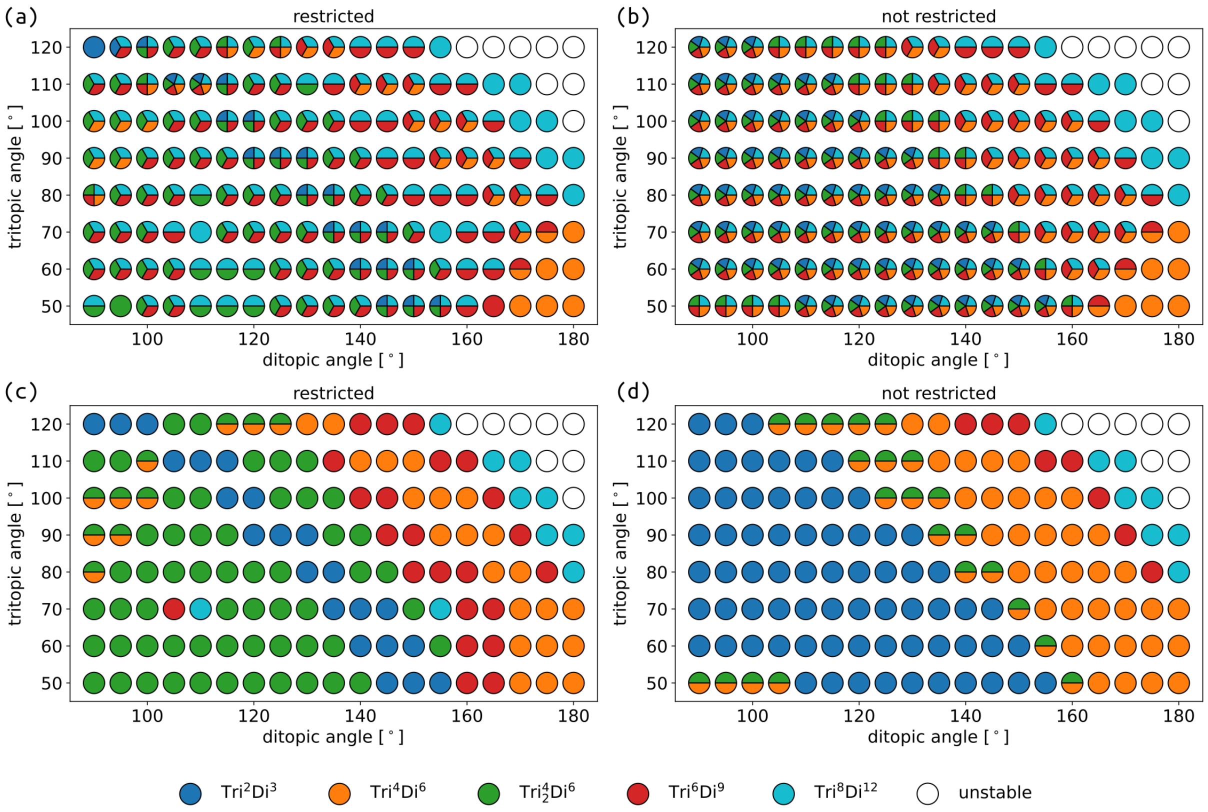

Fig. S44: Accessible topology maps for topologies with tritopic and ditopic building blocks and torsion restrictions (left) on and (right) off. The top shows mixtures, bottom shows only the smallest accessible topology. The mapping of colour to topologies is given in the legend.

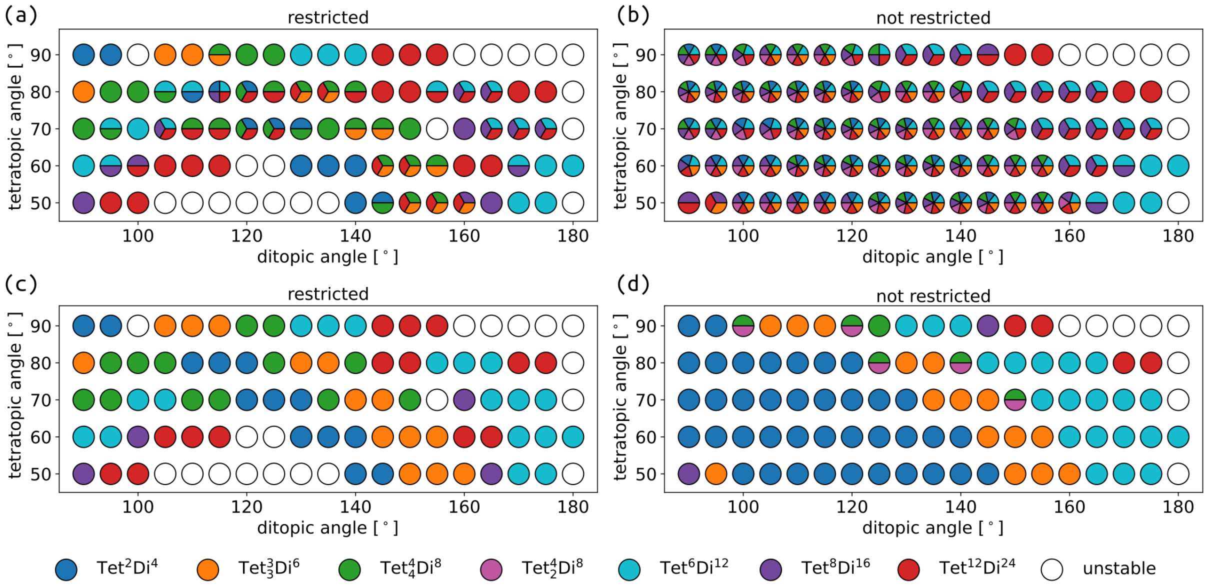

Fig. S45: Accessible topology maps for topologies with tetratopic and ditopic building blocks and torsion restrictions (left) on and (right) off. The top shows mixtures, bottom shows only the smallest accessible topology. The mapping of colour to topologies is given in the legend.

S11. Shape analysis

We calculate shape measures for each cage structure based on the deviation from in-built ideal shapes in *SHAPE* v2.1 software[S3] with vertices 3, 4, 6 and 8.[S4–S7] The tritopic, tetratopic or ditopic building block central beads (n, m, c, respectively) are extracted from a cage structure and parsed into the SHAPE software. Default settings and no central atom are applied in the SHAPE calculation. The ideal shapes for each topology were chosen as that with the highest percentage of cages with shape measure, s, less than 2 with the torsion restrictions (Fig. S46 and Fig. S47). For **Tri$^{4}_{2}$Di^{6}** ditopic, we chose the same shape as **Tri^{4}Di^{6}** because all s are high. The value of $s < 2$ is arbitrary but small enough such that we are focusing on the near-ideal cases. Table S3 shows the selected reference shapes. The shape naming convention is defined in the *SHAPE* software, where the first acronym describes a specific shape and the ending number is the number of vertices in a shape. For example, TP-3 is

the trigonal planar shape with three vertices.

Fig. S48, Fig. S49, Fig. S50 and Fig. S51 show the distribution of shape measures over all cages for each topology. Fig. S52, Fig. S53, Fig. S54 and Fig. S55 show the map of building block angles to stability and ideal shape difference.

Table S3: Definition of topologies studied in this work and their associated reference, ideal shapes.

topology	tri/tetratopic shape	ditopic shape
$\mathbf{Tri^2Di^3}$	-	TP-3
$\mathbf{Tri^4Di^6}$	T-4	OC-6
$\mathbf{Tri^4_2Di^6}$	SP-4	OC-6
$\mathbf{Tri^6Di^9}$	TPR-6	-
$\mathbf{Tri^8Di^{12}}$	CU-8	-
$\mathbf{Tet^2Di^4}$	-	SP-4
$\mathbf{Tet^3_3Di^6}$	TP-3	-
$\mathbf{Tet^4_4Di^8}$	SP-4	-
$\mathbf{Tet^4_2Di^8}$	T-4	-
$\mathbf{Tet^6Di^{12}}$	OC-6	-
$\mathbf{Tet^8Di^{16}}$	SAPR-8	-
$\mathbf{Tet^{12}Di^{24}}$	-	-
$\mathbf{Tet^6Tri^8}$	OC-6/CU-8	-

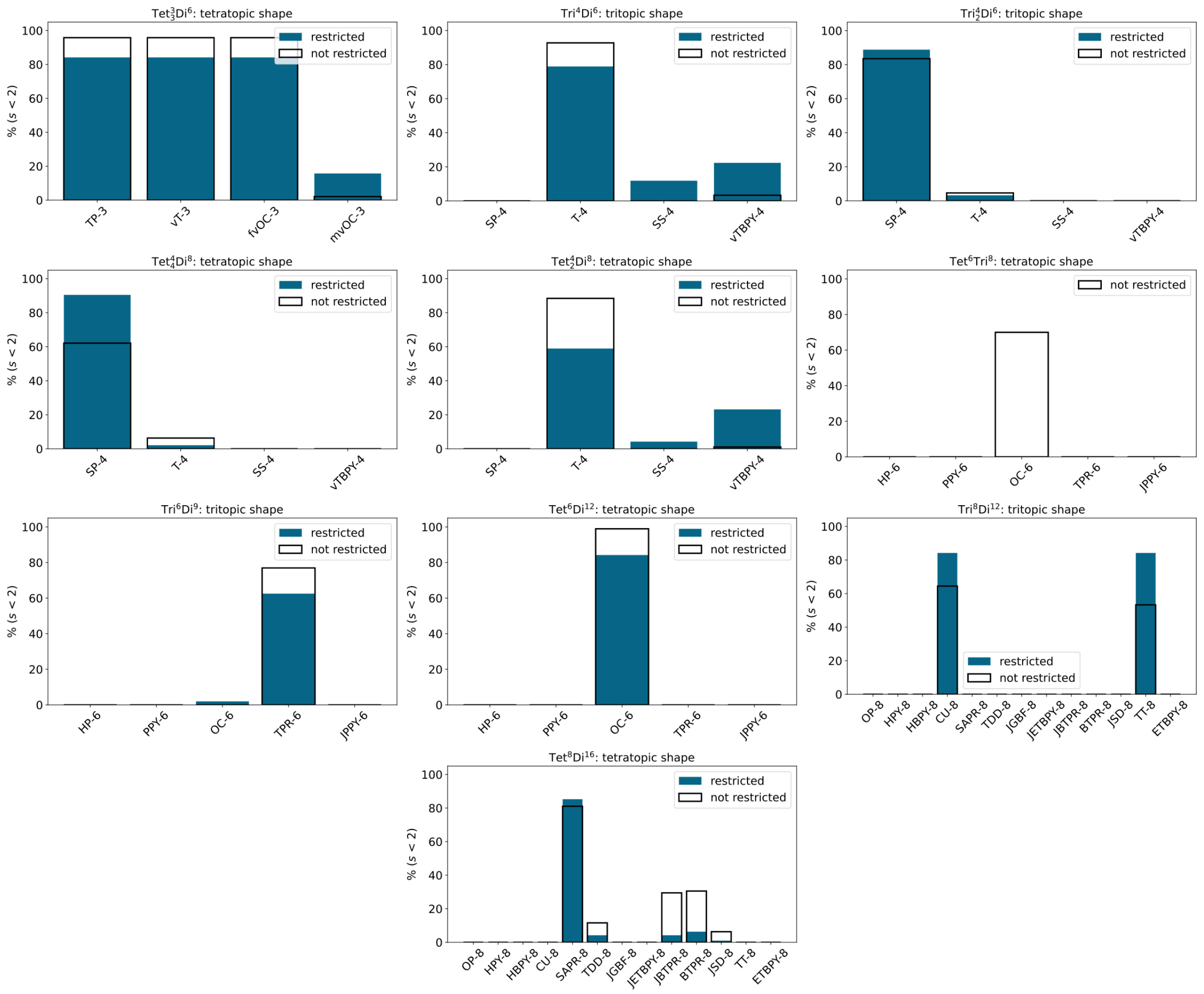

Fig. S46: Percentage of cages with $s < 2$ of all cages for topologies where shape was measured, with and without restricted torsion. Plots show either the tritopic or tetratopic building block shape measures (tetratopic for $\mathbf{Tet^6Tri^8}$ topology).

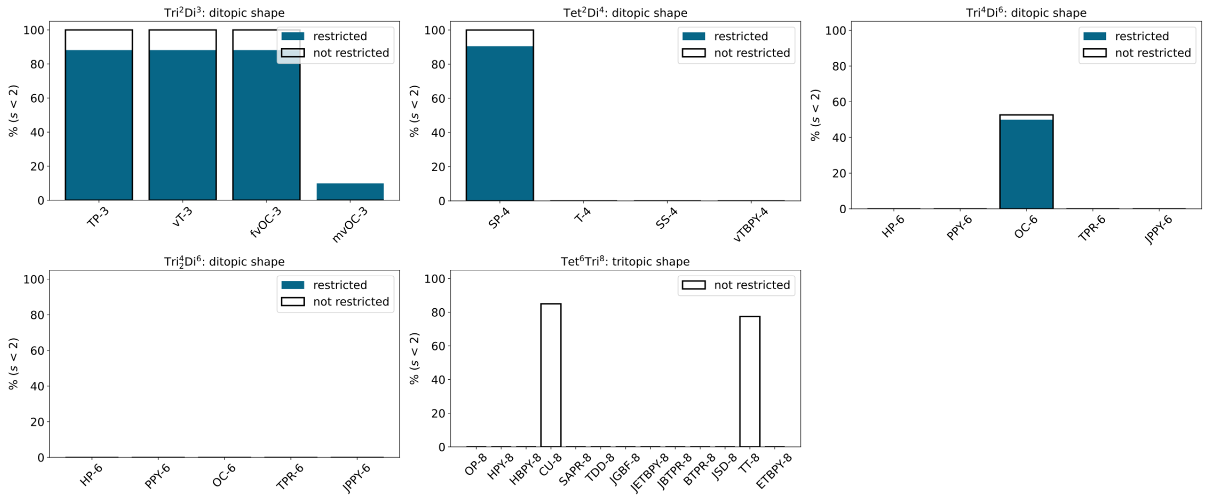

Fig. S47: Percentage of cages with $s < 2$ of all cages for topologies where shape was measured, with and without restricted torsion. Plots show the ditopic building block shape measures (tritopic for **Tet^{6}Tri^8** topology).

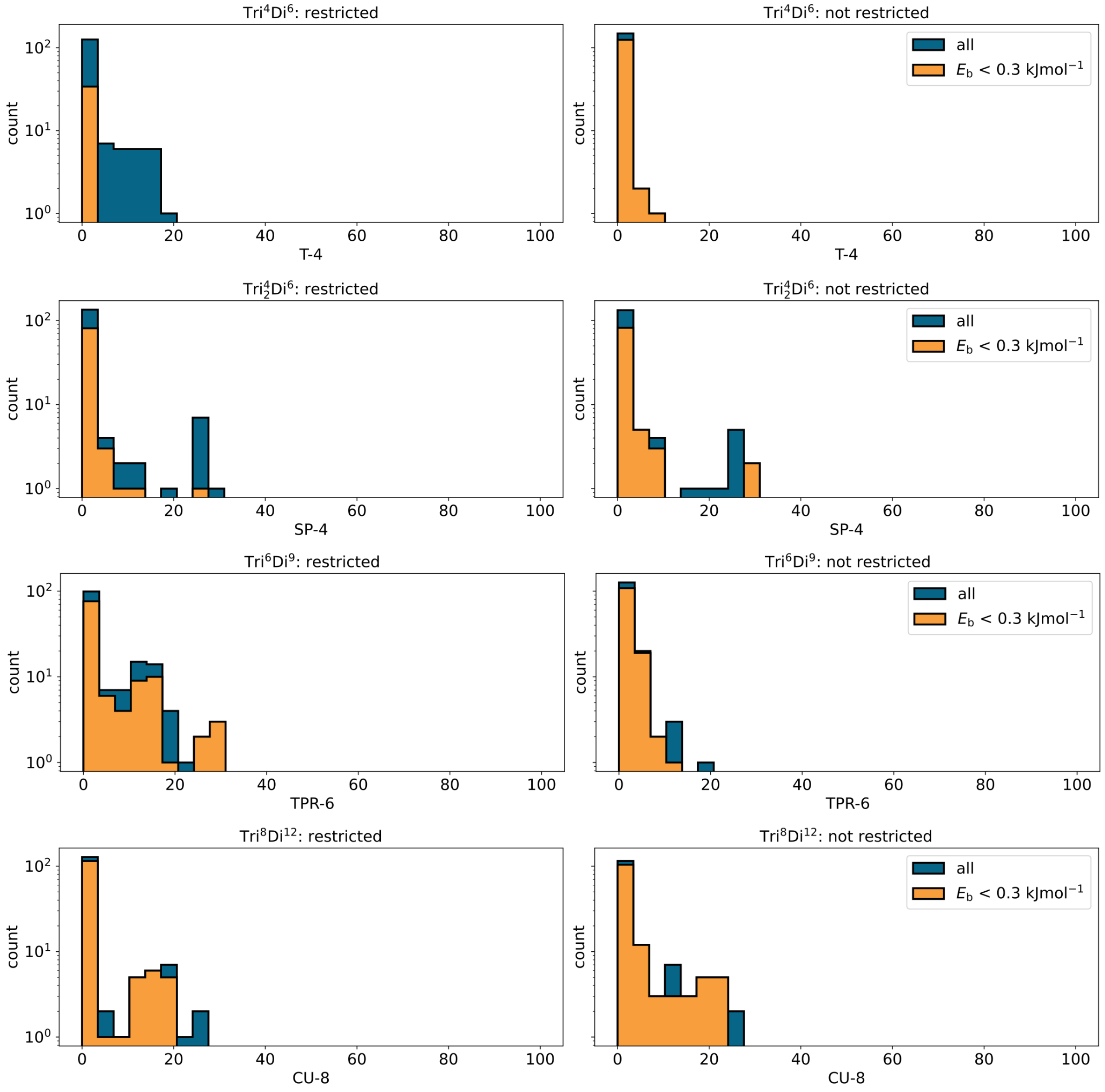

Fig. S48: Distribution of (left) torsion-restricted and (right) torsion-unrestricted deviation from ideal shapes for tritopic building blocks for **Tri^{4}Di^6**, **Tri$^4_2$Di^6**, **Tri^{6}Di^9** and **Tri^{8}Di^{12}** topologies. Blue distributions are all cages, and orange is for stable cages. This data is on a log scale due to the high proportion of cages near zero.

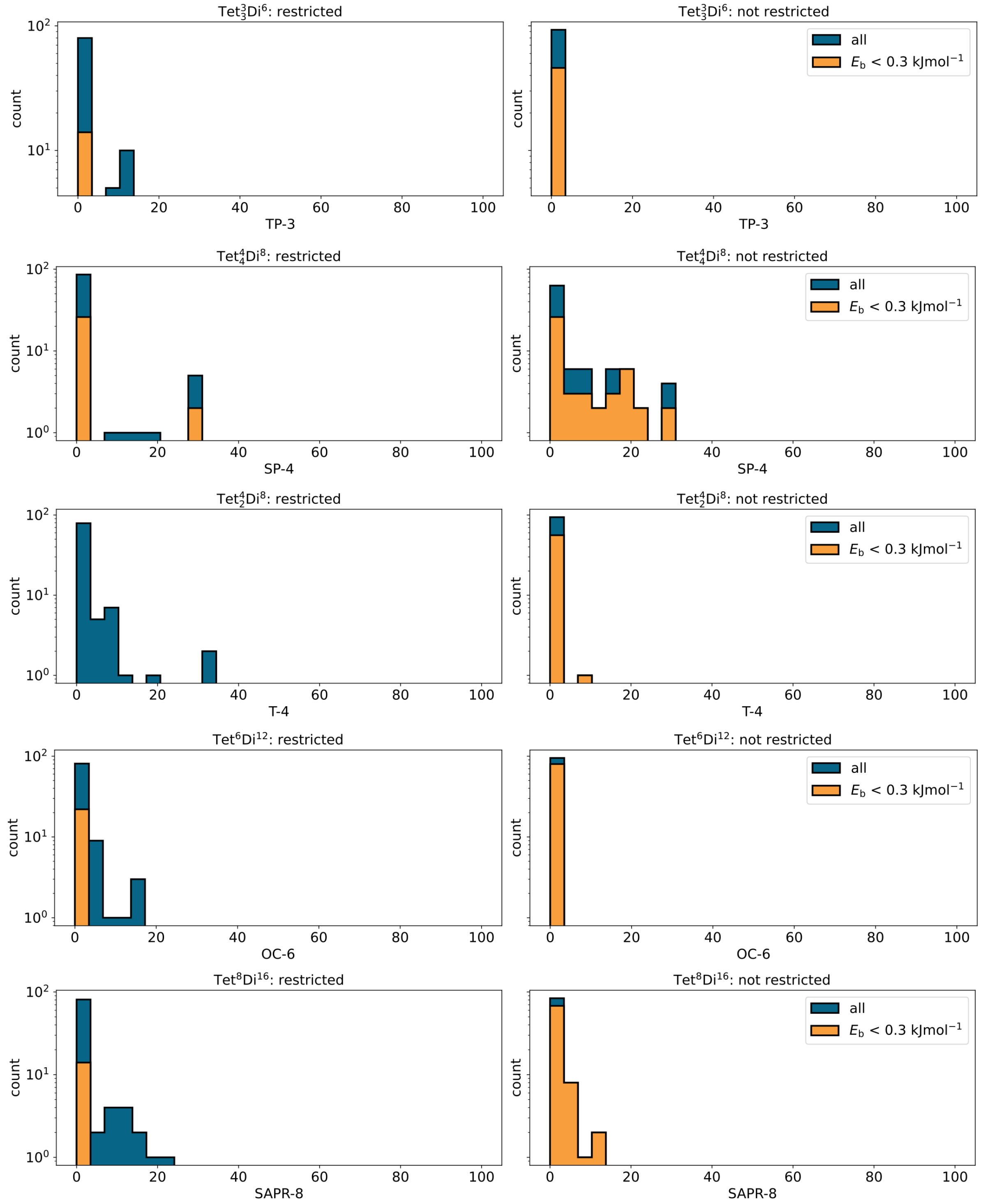

Fig. S49: Distribution of (left) torsion-restricted and (right) torsion-unrestricted deviation from ideal shapes for tetratopic building blocks for **Tet$^3_3$Di^6**, **Tet$^4_4$Di^8**, **Tet$^4_2$Di^8**, **Tet^{6}Di^{12}** and **Tet^{8}Di^{16}** topologies. Blue distributions are all cages, and orange is for stable cages. This data is on a log scale due to the high proportion of cages near zero.

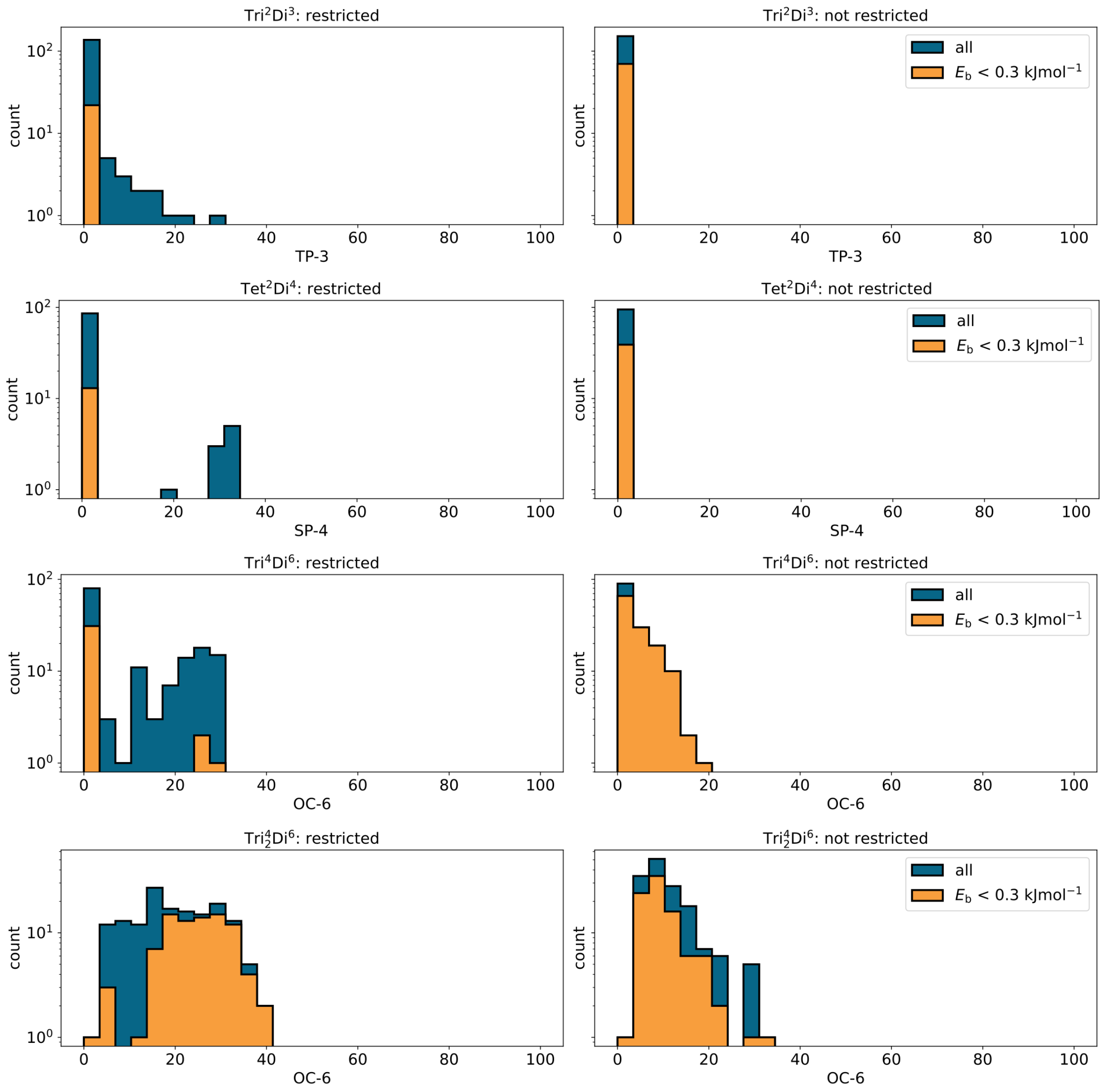

Fig. S50: Distribution of (left) torsion-restricted and (right) torsion-unrestricted deviation from ideal shapes for ditopic building blocks for **Tri^{2}Di^3**, **Tet^{2}Di^4**, **Tri^{4}Di^6** and **Tri$_2^4$Di^6** topologies. Blue distributions are all cages, and orange is for stable cages. This data is on a log scale due to the high proportion of cages near zero.

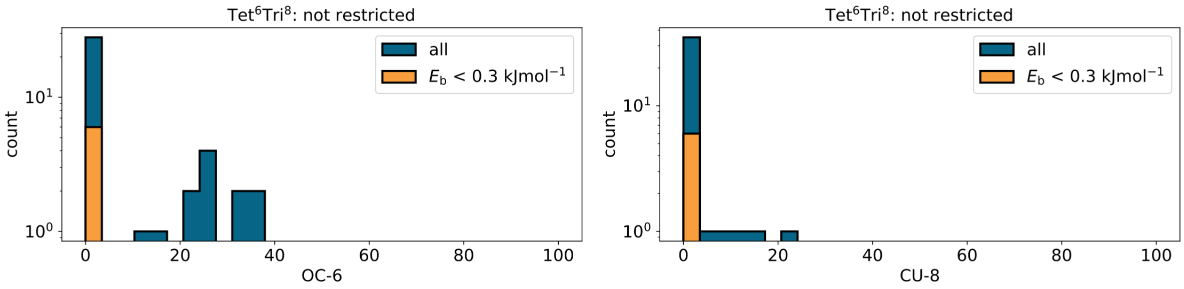

Fig. S51: Distribution of (left) tetratopic and (right) tritopic building block deviation from ideal shapes for the **Tet^{6}Tri^8** topology. Blue distributions are all cages, and orange is for stable cages. This data is on a log scale due to the high proportion of cages near zero.

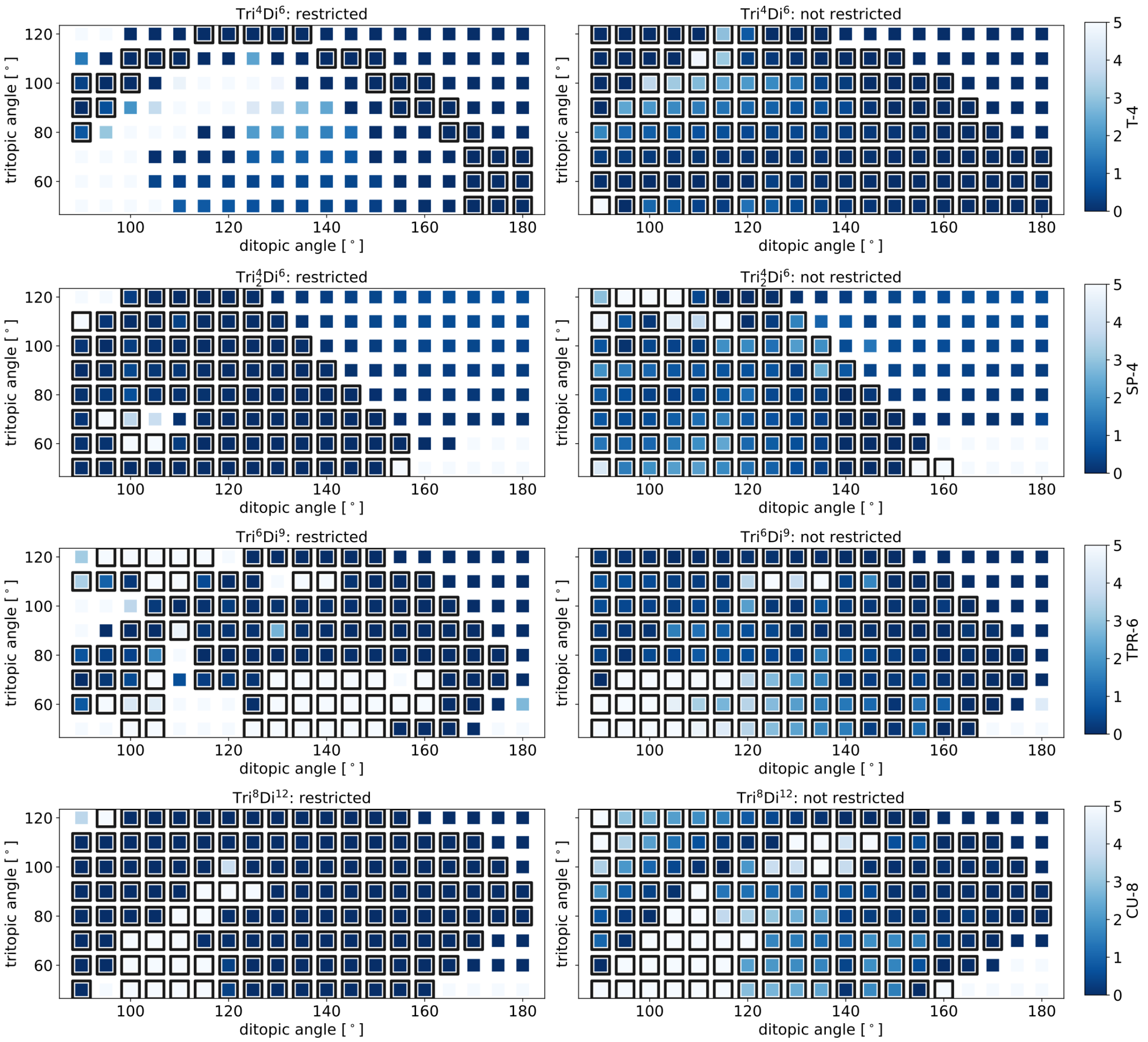

Fig. S52: Map of shape and angle relationship of (left) torsion-restricted and (right) torsion-unrestricted shapes for tritopic building blocks in **Tri^{4}Di^6**, **Tri$^4_2$Di^6**, **Tri^{6}Di^9** and **Tri^{8}Di^{12}** topologies. Black squares highlight stable cages (E_b < 0.3 kJ mol^{-1}). The colour map and target shape are given on the right-hand side.

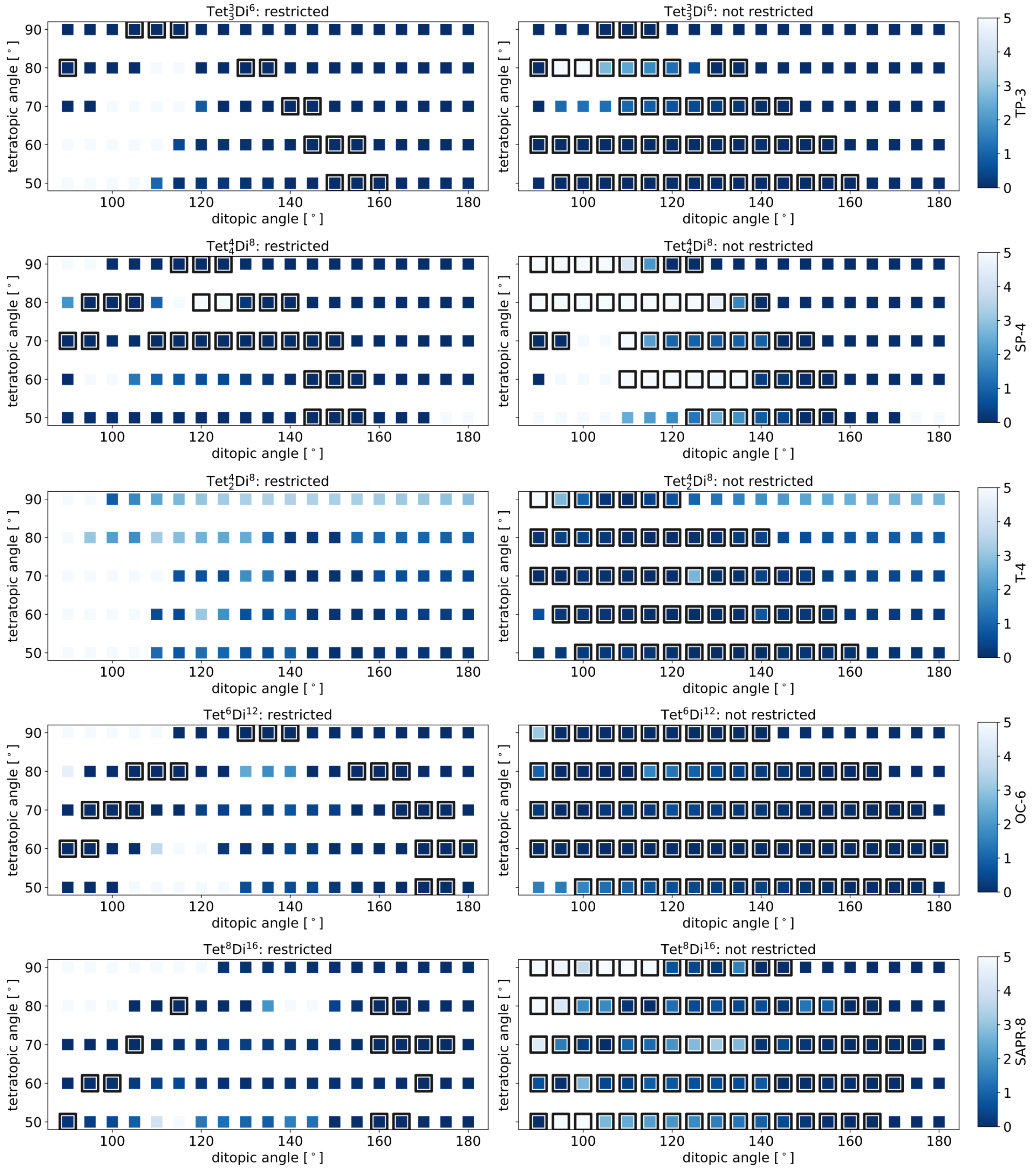

Fig. S53: Map of shape and angle relationship of (left) torsion-restricted and (right) torsion-unrestricted shapes for tetratopic building blocks in **Tet$^3_3$Di^6**, **Tet$^4_4$Di^8**, **Tet$^4_2$Di^8**, **Tet^{6}Di^{12}** and **Tet^{8}Di^{16}** topologies. Black squares highlight stable cages (E_b < 0.3 kJ mol^{-1}). The colour map and target shape are given on the right-hand side.

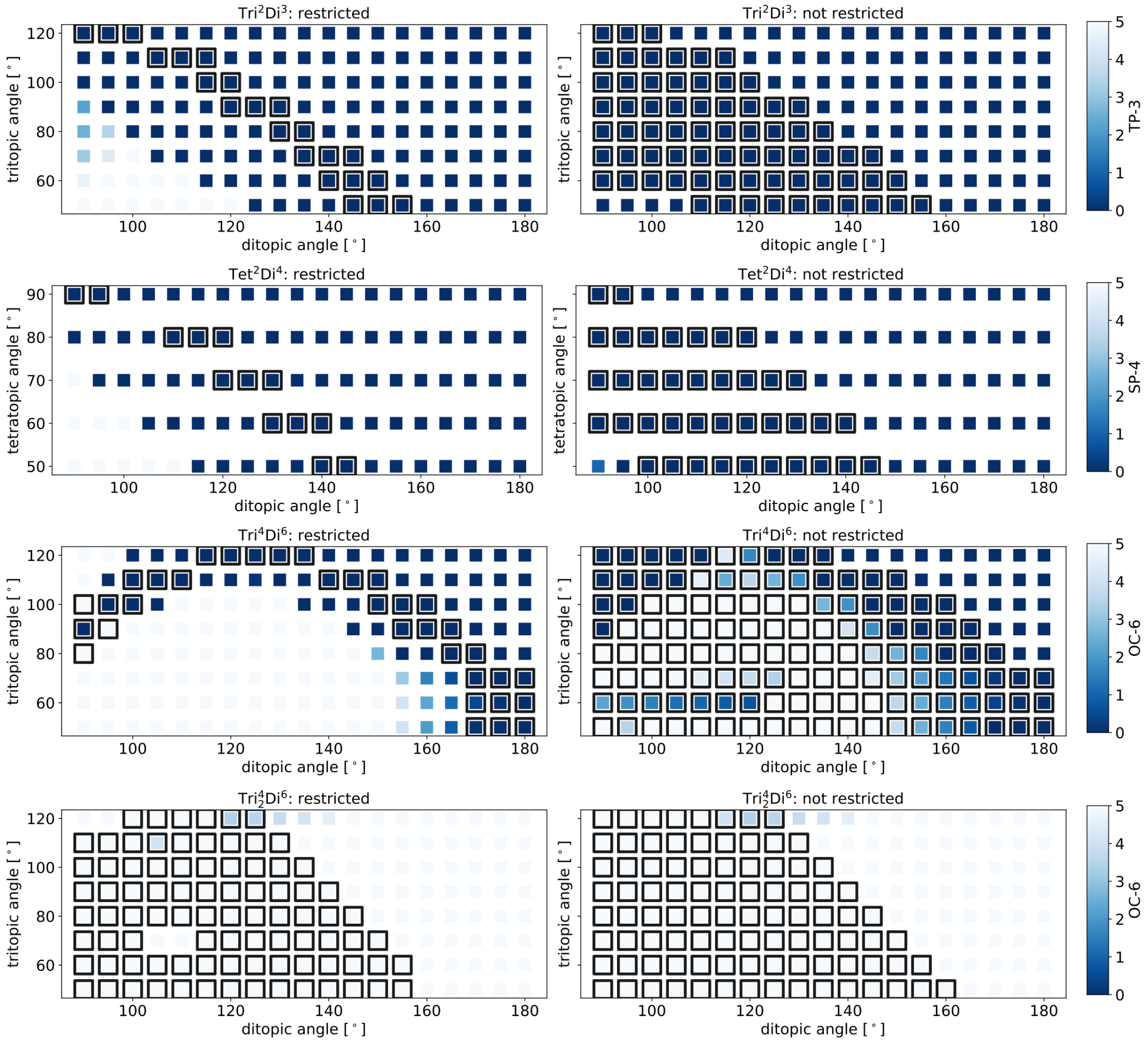

Fig. S54: Map of shape and angle relationship of (left) torsion-restricted and (right) torsion-unrestricted shapes for ditopic building blocks in **Tri^{2}Di^3**, **Tet^{2}Di^4**, **Tri^{4}Di^6** and **Tri$^4_2$Di^6** topologies. Black squares highlight stable cages ($E_{\mathrm{b}} < 0.3\,\mathrm{kJ\,mol^{-1}}$). The colour map and target shape are given on the right-hand side.

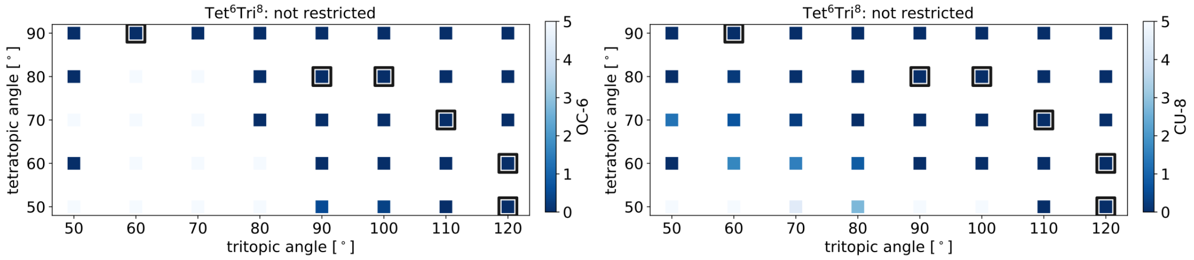

Fig. S55: Map of shape and angle relationship of (left) tetratopic and (right) tritopic building block shapes for the **Tet^{6}Tri^8** topology. Black squares highlight stable cages ($E_{\mathrm{b}} < 0.3\,\mathrm{kJ\,mol^{-1}}$). The colour map and target shape are given on the right-hand side.

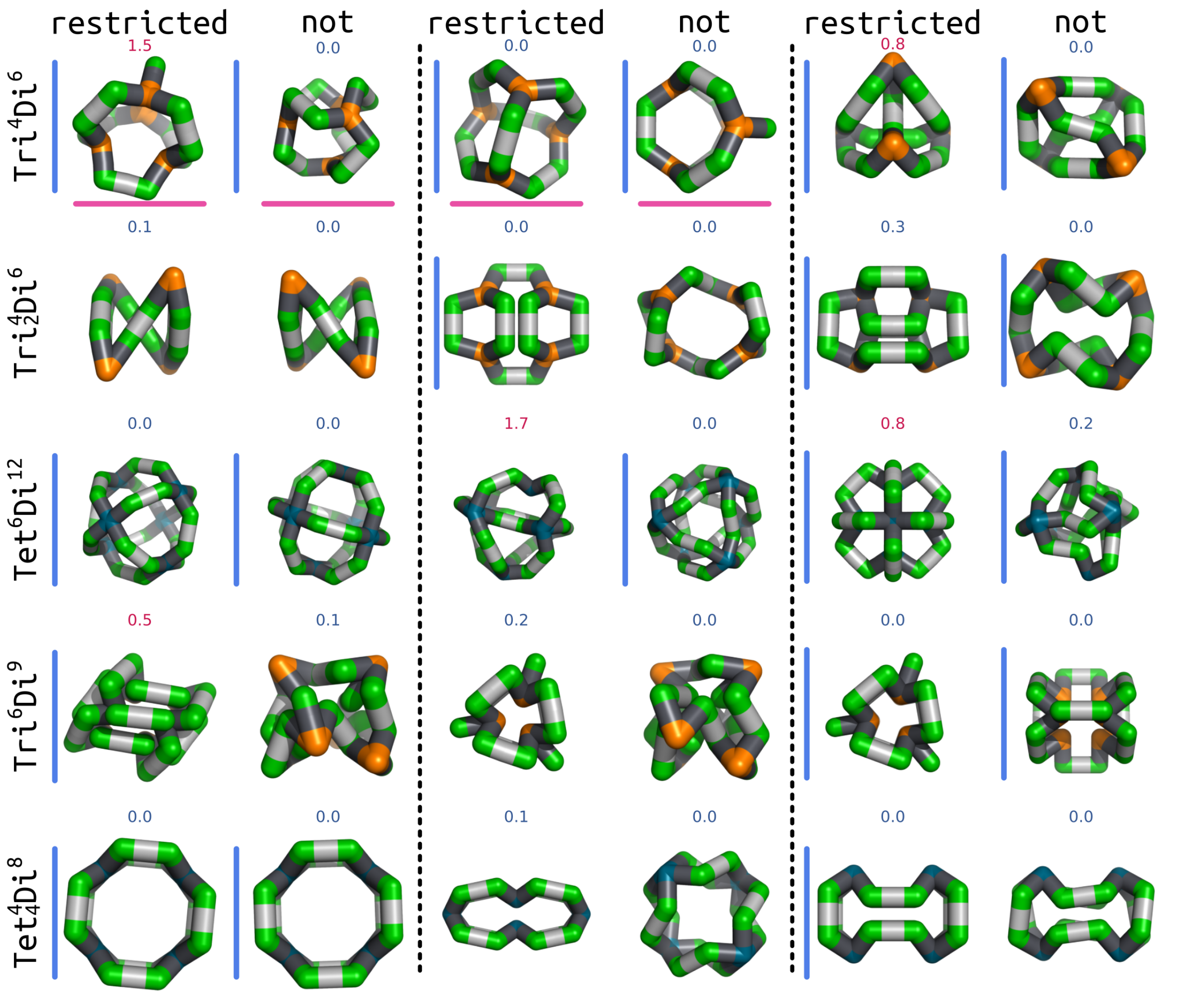

Fig. S56: Example pairs of structures with and without torsion restrictions. The topology for each row is on the left-hand side. Blue lines indicate the structure has an ideal shape deviation near zero for their tritopic or tetratopic building blocks, while pink lines indicate the same for ditopic building blocks. The numbers are the E_{b} values for each structure.

S12. Property space of cage models

Given a cage structure, we calculate three structural properties: the radius of gyration, R_{g}, the maximum cage-centroid-to-bead distance (or maximum diameter), D, and the pore size. Regarding the pore size, because our beads do not represent the dimensions of actual atoms or building blocks, we simply approximate pore size as the minimum cage-centroid-to-bead distance in a model. While simple, this is similar to how software, like *pyWindow*,[S8] measure pore size.

Here, we look at the relationships between pore size and other input or structural parameters to see if there are any useful design rules. Fig. S57 shows the distributions of cage properties, highlighting that all size measures follow the size of the cage topologies. However, some larger structures (with a larger maximum diameter, D, or R_{g}) must exist with small or negligible pore sizes, indicating collapse. Fig. S58 clarifies this by showing the distributions of the ratio of these properties, where R_{g}/D is almost constant but large distributions are present for pore size comparisons.

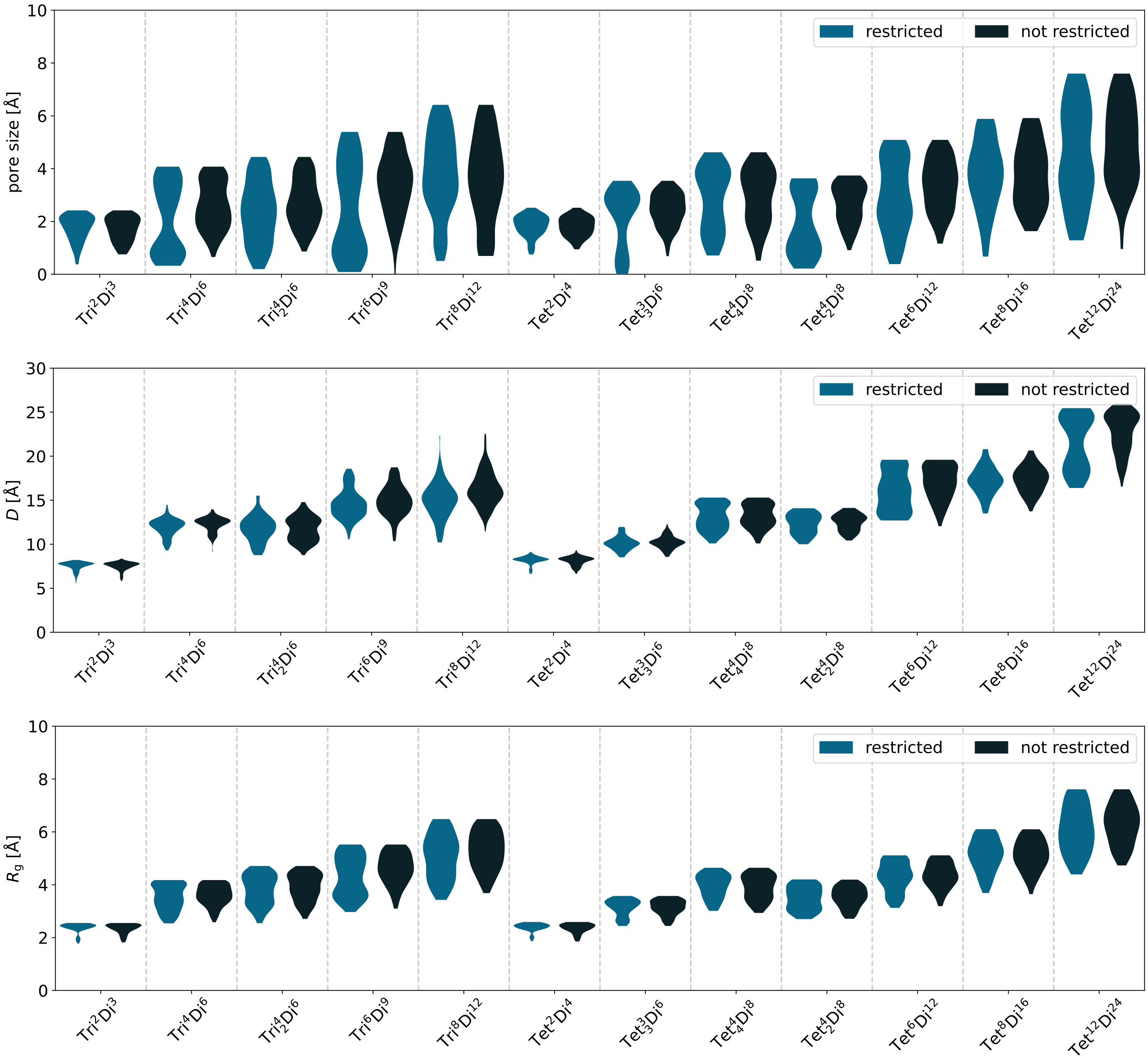

Fig. S57: Distributions of properties for all topologies (excluding $\mathbf{Tet^6Tri^8}$) with and without torsion restrictions. In order from top-to-bottom: pore size, maximum diameter D and R_{g}.

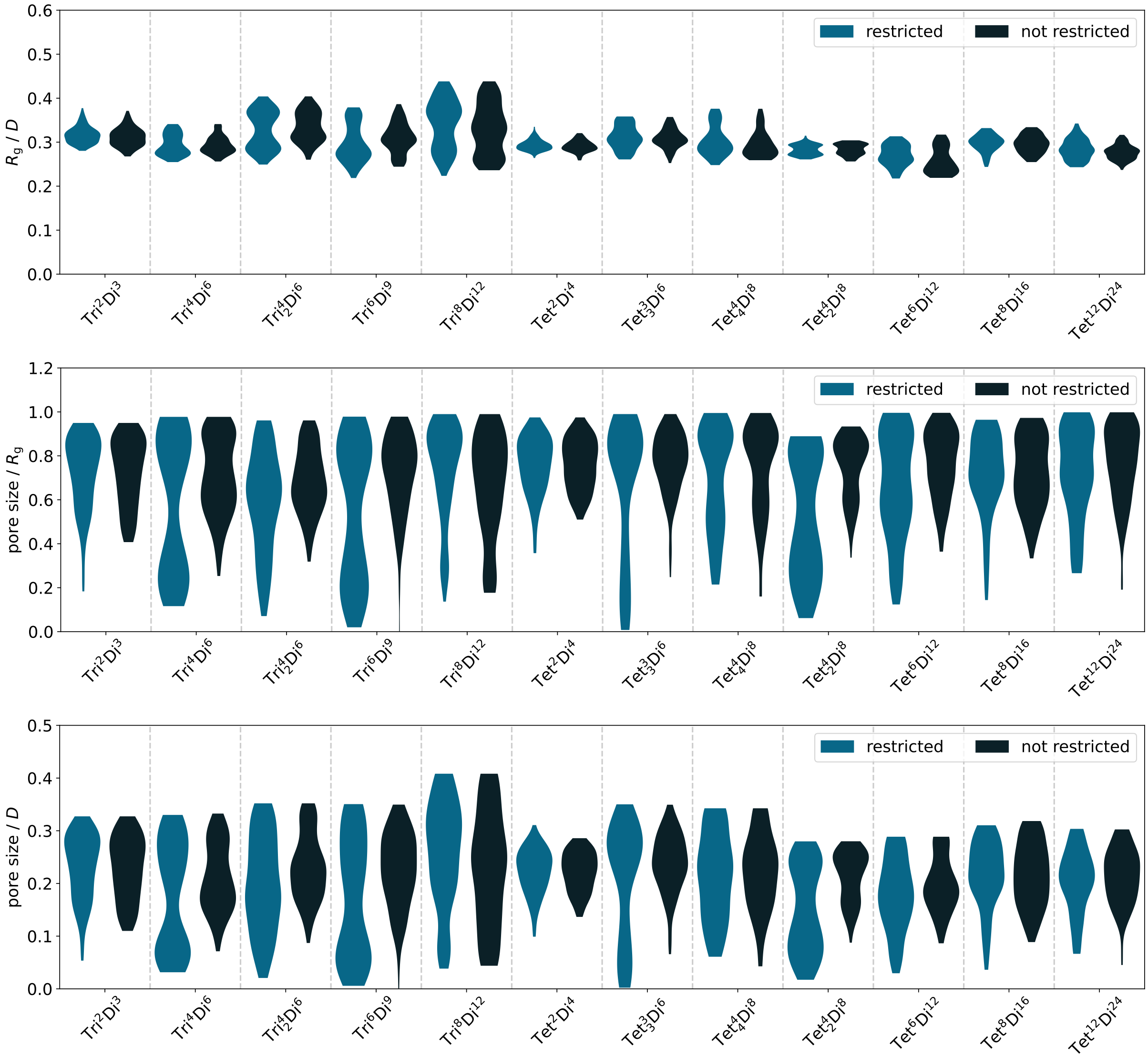

Fig. S58: Distributions of properties for all topologies (excluding **Tet^{6}Tri^8**) with and without torsion restrictions. In order from top-to-bottom: R_g/D, pore size/R_g, and pore size/D.

Fig. S59 shows the pore sizes as a function cage size, building block properties and cage structural properties after optimisation with and without restricted torsions. The structures shown are per building block pair, where the smallest stable structure is shown if it is not expected to form a mixture. Fig. S59(a) and (d) show the relationship between pore size and cage stoichiometry (topology) and size, respectively. Interestingly, some topologies show a far wider range of pore sizes in their stable structures. For example, with ten building blocks, the **Tri^{4}Di^6** and **Tri$_2^4$Di^6** topologies show a wide range of pore sizes (similarly for the **Tri^{6}Di^9** topology). This suggests the chance for more tunability in properties but also may suggest more difficulties in their synthesis due to configurational flexibility. Fig. S59(b) shows that the connectivity of the building blocks has a small impact on the distribution of pore sizes, where having four-connected nodes increases the median pore size. However, it is not clear if this is a result of accessible configurations or the topologies available (tetratopic-containing topologies include the **Tet^{12}Di^{24}** topology (36 building blocks) while tritopic-containing topologies stop at **Tri^{8}Di^{12}** (20 building blocks)).

Fig. S59(e) shows that cages with restricted torsions (black) deviate from the correlation between cage size (R_g) and pore size, which is observed to be stronger for the unrestricted case (pink). Most interestingly is that Fig. S59(c) and (f) show that the angle in the building block with the largest coordination number has a less strong relationship with pore size than the target ditopic internal angle, where target internal angles closer to 180° lead to larger pore sizes. Indeed there seems to be a switch between two regimes around 140° in (f). This follows the finding that larger internal angles tend to favour larger topologies.

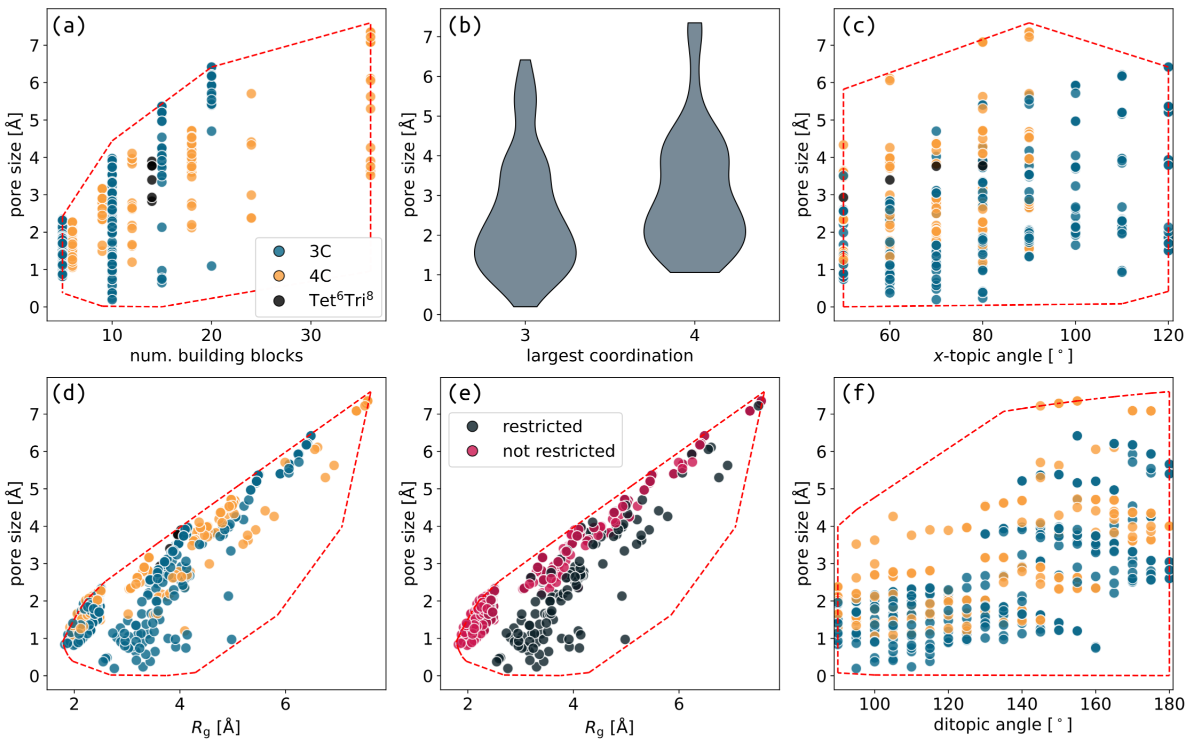

Fig. S59: Property spaces of the smallest stable, sorted cages for each building block pair in this work. Plots (a), (c), (d) and (f) show the pore size, coloured by the largest coordination number, as a function of the number of building blocks in each cage, the angle in the largest coordination number building block (tritopic or tetratopic), the cage radius of gyration, and the target ditopic angle, respectively. (b) shows the distribution of the pore size for cages with the largest coordination number of 3 or 4. (e) shows the cage pore sizes as a function of the cage radius of gyration coloured by torsion restriction. The convex hull of the full cage space (not just stable structures) is shown as red dashed lines.

S13. Using the data generated in this work

The data generated by our toy models may be useful in understanding or predicting self-assembly outcomes. To this end, we have made them accessible through an easy-to-use database (https://andrewtarzia.github.io/selfsort/), which we run through now. In the future, we intend on updating this database, and making a more responsive web-app.

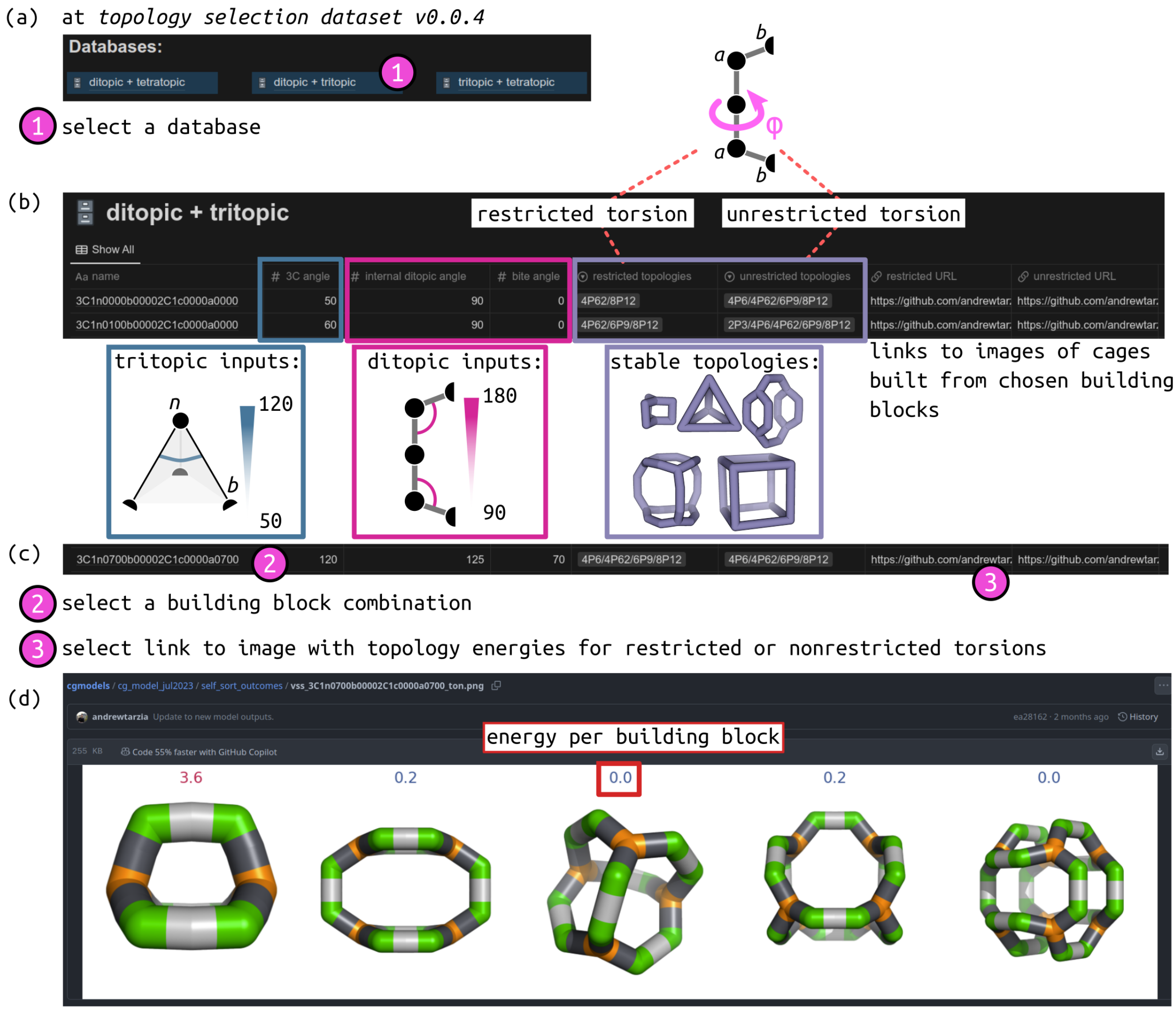

Fig. S60: Using the database. (a) Following the link for topology selection database, the user will see a database instruction list and three databases to choose from. Here, we select the ditopic + tritopic building block database (1). (b) In the database, each building block combination is a row. The column '3C angle' states the tritopic building block input of pyramid angle. The columns 'internal ditopic angle' and 'bite angle' state the ditopic building block input of internal angles, which correspond to bite angle in some cases. The next four columns have the stable topologies and link to images for either the torsion restricted or unrestricted case. (c) Step (2) is to find the desired building block combination (based on the input angle values) and then the user can select (step (3)) a link to a GitHub database with images of the structures and E_{b} of that building block combination in all topologies (d).

References

(S1) Santolini, V.; Miklitz, M.; Berardo, E.; and E. Jelfs, K. "Topological Landscapes of Porous Organic Cages." *Nanoscale*, 2017. **9**(16):5280–5298. doi:10.1039/C7NR00703E

(S2) Turcani, L.; Tarzia, A.; Szczypiński, F.; and Jelfs, K.E. "Stk: An Extendable Python Framework for Automated Molecular and Supramolecular Structure Assembly and Discovery." *J Chem Phys*, 2021. **154**(21):214102. doi:10.1063/5.0049708

(S3) Pinsky, M. and Avnir, D. "Continuous Symmetry Measures. 5. The Classical Polyhedra." *Inorg Chem*, 1998. **37**(21):5575–5582. doi:10.1021/ic9804925

(S4) Ruiz-Martínez, A.; Casanova, D.; and Alvarez, S. "Ligand Association/Dissociation Paths and Ill-Defined Coordination Numbers." *Chem Eur J*, 2010. **16**(22):6567–6581. doi:10.1002/chem.200902996

(S5) Cirera, J.; Alemany, P.; and Alvarez, S. "Mapping the Stereochemistry and Symmetry of Tetracoordinate Transition-Metal Complexes." *Chem Eur J*, 2004. **10**(1):190–207. doi:10.1002/chem.200305074

(S6) Alvarez, S.; Avnir, D.; Llunell, M.; and Pinsky, M. "Continuous Symmetry Maps and Shape Classification. The Case of Six-Coordinated Metal Compounds." *New J Chem*, 2002. **26**(8):996–1009. doi:10.1039/B200641N

(S7) Casanova, D.; Llunell, M.; Alemany, P.; and Alvarez, S. "The Rich Stereochemistry of Eight-Vertex Polyhedra: A Continuous Shape Measures Study." *Chem Eur J*, 2005. **11**(5):1479–1494. doi:10.1002/chem.200400799

(S8) Miklitz, M. and Jelfs, K.E. "Pywindow: Automated Structural Analysis of Molecular Pores." *J Chem Inf Model*, 2018. **58**(12):2387–2391. doi:10.1021/acs.jcim.8b00490
